# Contingency management to promote smoking cessation in people experiencing homelessness: Leveraging the electronic health record in a pilot, pragmatic randomized controlled trial

**DOI:** 10.1371/journal.pone.0278870

**Published:** 2022-12-16

**Authors:** Melanie F. Molina, Sharon M. Hall, Maxine Stitzer, Margot Kushel, Deepalika Chakravarty, Maya Vijayaraghavan

**Affiliations:** 1 Department of Emergency Medicine, University of California, San Francisco, San Francisco, CA, United States of America; 2 Department of Psychiatry and Weill Institute of Neurosciences, University of California, San Francisco, San Francisco, CA, United States of America; 3 Friends Research Institute, Baltimore, MD, United States of America; 4 Division of Vulnerable Populations, University of California, San Francisco, San Francisco, CA, United States of America; 5 Center for Aids Prevention Studies, University of California, San Francisco, San Francisco, CA, United States of America; 6 Division of General Internal Medicine, University of California, San Francisco, San Francisco, CA, United States of America; GERMANY

## Abstract

**Background:**

Cigarette smoking is disproportionately high among people experiencing homelessness (PEH). Contingency management (CM) is a strategy that has shown considerable efficacy for smoking cessation and has been used in short-term studies of smoking abstinence in PEH. We describe a pilot, pragmatic randomized controlled trial protocol, which leverages an electronic health record (EHR) infrastructure to assess the feasibility and acceptability of an extended CM intervention to improve long-term abstinence in PEH.

**Methods:**

We will conduct the study at three safety-net clinics in San Francisco among 90 adults experiencing homelessness who smoke cigarettes currently and have a desire to quit. We will encourage all participants to receive smoking cessation services that include behavioral counseling and pharmacotherapy through their clinics. We will randomly assign participants to an extended CM intervention group with escalating incentives contingent on abstinence or to a control group with fixed incentives for attending study visits. We will use the EHR to recruit participants, track receipt of counseling and pharmacotherapy during clinical care, and communicate with providers on participants’ progress. CM participants will get escalating incentives for demonstration of carbon monoxide-verified abstinence over 6 months, with a total possible earnings of $475. Control participants will receive a fixed incentive of $5 for attending study visits, totaling $125. We will conduct the carbon-monoxide verified abstinence assessments—which will determine CM incentive amounts—daily during week 1, bi-weekly through week 4, weekly through week 13, and monthly through week 24. Measures of feasibility and acceptability, both quantitative and qualitative, will include assessments of screening and recruitment, adherence to study visits, engagement in smoking cessation clinical care, retention, and participant satisfaction. One of the primary clinical outcomes will be biochemically verified 7-day point prevalence abstinence at 6 months. We will measure secondary outcomes, which will include 7-day point prevalence abstinence at 2 weeks, 3 and 12 months.

**Discussion:**

This trial will allow us to assess the feasibility and acceptability of a CM cessation intervention among PEH. The protocol’s clinical setting and use of EHRs gives it significant potential for scalability. If found to be feasible, acceptable, and subsequently efficacious in a larger trial, the intervention could reduce tobacco-related health disparities by increasing long-term smoking abstinence among this vulnerable population.

**Trial registration:**

ClinicalTrials.gov NCT04982952. Registered on July 29, 2021.

## Introduction

Tobacco use is disproportionately high among people experiencing homelessness (PEH), with a prevalence of 70% compared to 13.7% in the general population [1, 2]. Tobacco-related cardiovascular disease and cancers are among the leading causes of morbidity and mortality in adults experiencing homelessness [3–7]. Mental illness and substance use disorders are common among PEH and represent risk factors for both smoking and poor smoking-related health outcomes [8]. PEH make quit attempts as frequently as the general population, but quit successfully less often [9–11]. Clinical trials of behavioral counseling and pharmacotherapy—the standard of care for smoking cessation—have increased short-term quit attempts but have not demonstrated long-term smoking abstinence among PEH [12–16]. Long-term abstinence, defined as abstinence for 6 months or more, is a strong predictor of successful cessation [17].

Contingency management (CM) is an efficacious behavior change strategy that reinforces positive health behaviors with incentives (e.g., cash) and has been shown in clinical trials to reduce tobacco and substance use in the general population [18–21]. Recent research, including uncontrolled pilot studies [22, 23] and pilot randomized controlled trials (RCTs), [24, 25] have demonstrated the feasibility of short-term (i.e., 8 weeks or less) CM to increase quit attempts among PEH. However, these studies did not explore ways to promote long-term abstinence. Evidence suggests that CM implemented for 12 weeks or longer can be efficacious in promoting long-term abstinence beyond 6 months [18, 26–31]. Currently, there are no feasibility trials evaluating the ability of extended CM to promote long-term abstinence among PEH.

Impactful cessation interventions not only need to be efficacious, but also feasible to implement in community or clinical practice settings [32, 33]. Given that the majority of PEH in the United States of America (USA) seek medical care in safety-net health settings [34, 35], a pragmatic clinical trial—designed to evaluate the effectiveness of an intervention in one of these practice settings [36]—could be ideally suited for this population and help bring smoking cessation interventions to scale. Furthermore, specially tailored techniques may be needed to facilitate participation success of PEH in clinical trials.

Electronic health records (EHRs) are increasingly being used to facilitate large-scale, pragmatic RCTs [37, 38] and may be especially useful for PEH. Although most commonly used to assess decision-support interventions, EHRs can also be used for patient recruitment, service utilization, and outcome assessment in RCTs with interventions performed outside of the EHR [38, 39]. Maintaining sustained contact with PEH is a challenge. However, our group and others have developed recruitment and retention techniques to minimize attrition in clinical trials [40–49].

### Study objectives

We will conduct a parallel-comparison, pilot pragmatic RCT of an extended (6-month) CM intervention for smoking cessation among PEH who are engaged in care at three safety-net clinics. The goal is to increase long-term (6 months or longer) cigarette smoking abstinence. We will leverage an existing EHR infrastructure to recruit patients, create a registry of enrollees to obtain information on receipt of counseling and pharmacotherapy during clinical care, and communicate with providers on participants’ trial progress. We will thus provide a model of EHR-facilitated study implementation that can be replicated in other safety-net clinics and healthcare organizations. The main objectives of this pilot pragmatic RCT will be to assess the feasibility and acceptability of the CM intervention. In addition, we will assess the feasibility of utilizing the EHR as part of the study protocol implementation as well as the collection of abstinence outcomes for up to 12 months (including 6 months post-intervention) in PEH.

## Materials and methods

### Study setting

We will conduct the study in three safety-net clinics in San Francisco, California. The clinics are part of the San Francisco Health Network—a network of primary care, behavioral health, and acute care clinics. One of the clinics serves a large proportion of PEH (smoking prevalence 44%). The second is an academic primary care clinic located in a safety-net hospital (smoking prevalence 15.2%). The third is an academic primary care clinic located in a safety-net hospital and serves people living with HIV (smoking prevalence 35.4%). We chose these sites based on a needs assessment demonstrating a higher prevalence of tobacco use than the state prevalence of 10% [50] and the presence of an existing clinical infrastructure to deliver guideline-recommended cessation care, including behavioral counseling and pharmacotherapy [51, 52]. These clinics have already incorporated a tobacco registry within the Epic EHR to improve delivery of tobacco smoking cessation services by enabling tracking of interventions and missed opportunities in providing cessation care [53].

### Study population

We will offer study participation to all patients at the three study sites who are current smokers with an intention to quit smoking within the next 6 months. Eligibility criteria will include 1) having a primary care provider and receiving smoking cessation care at the clinic, including behavioral counseling and pharmacotherapy, 2) meeting criteria for homelessness as defined by the Homeless Emergency Assistance and Rapid Transition to Housing (HEARTH) Act or living in single room occupancy hotels temporarily over the past 2 years [54], 3) being a current smoker (at least 100 cigarettes in lifetime, daily smoking in the past 7 days and at least 5 cigarettes per day, verified by expired carbon monoxide (CO) ≥8 parts per million [ppm]) [55, 56] with an intention to quit smoking within the next 6 months, 4) being English proficient, and 5) being able to provide informed consent. Participants will be encouraged to attend usual smoking cessation care, which is available to all patients who receive clinical services at the three study sites. Exclusion criteria include any reason that precludes the use of nicotine replacement therapy (NRT) (e.g., pregnancy, myocardial infarction within the preceding 2 weeks).

### Recruitment and informed consent procedures

Health Insurance Portability and Accountability Act (HIPAA)- trained study staff will generate lists of patients within the Epic EHR who meet eligibility criteria based on housing and smoking status. We will ask providers in each of the clinics to review these lists of potential participants and provide permission for study staff to contact the individuals for eligibility screening. Separately, providers will also perform “warm hand-offs” or referrals of potential participants to study staff during in-person recruitment at the clinics. We will also incentivize participants to refer other individuals who meet study criteria for potential participation.

Study staff will screen potential participants for current smoking/housing status and assess whether they have contraindications to receiving medications for cessation. They will contact primary care providers (PCPs) for individuals who meet eligibility criteria but who may have contraindications to cessation medications to assess whether they can enroll in the study. We will invite those who meet preliminary eligibility criteria without contraindications to meet study staff at the recruitment site for the assessment of smoking status using CO testing. Participants whose expired CO is ≥8 ppm will be invited to enroll in the study. Study staff will describe study procedures and obtain written informed consent using the teach-to-goal method at the time of eligibility, which has been shown to increase comprehension of consent documents in vulnerable patients [57]. To do so, the study staff will give the participant a copy of the consent form, read the informed consent documents and encourage the participant to read along if they desire. The study staff will pause after each section of the consent to allow the participant to ask questions about that section. After reading the entire consent, the interviewer will ask the participants questions related to the content of the consent. Questions will address the procedures for the study, the risks of participation, the voluntary nature of research, the right of the participants to end participation at any point without consequence, the reimbursement for participation, and situations when study staff is legally bound to report participant disclosures (e.g., suicidality, homicidality). These questions will be used by the study staff to assess comprehension. For participants demonstrating a lack of understanding about one or more aspects of the research, the study staff will provide targeted education about misunderstood points. The questioning and targeted education process will be repeated until the participant demonstrates comprehension. If a given individual is unable to demonstrate comprehension after several rounds of questioning and targeted education, we will not enroll him/her/them into the study. Study staff will assess decisional capacity using the Macarthur Competence Assessment Tool for Clinical Research [58].

To mitigate the risks of transmission from COVID-19, all study procedures will take place outdoors in a public space in close proximity to the study clinics. The University of California, San Francisco (UCSF) Institutional Review Board (IRB) and the San Francisco Department of Public Health most recently approved the current version of the study protocol (Study #20–33166), including its consent procedures, on August 24, 2022.

### Randomization

Before initiating the study, we will randomly pre-assign sequential identification (ID) numbers to study arms in blocks of random size. We will stratify randomization based on time-to-first-cigarette after waking and the interviewer, so that each group will be matched on level of nicotine dependence and the interviewer who conducted the enrollment visit. At the time of enrollment, we will notify participants whether they were randomized to the intervention or the control group. We will randomize 45 participants to the intervention arm and 45 to the control arm.

### Cessation care for all participants

Cessation care at all our study clinics includes behavioral counseling and pharmacotherapy provided by the medical team as part of routine clinical care [24, 25]. If patients have insurance-related barriers to receiving nicotine replacement therapy (NRT), the study will provide a 12-week supply of NRT in the form of the transdermal patch and/or gum. HIPAA-trained study staff will access the EHR monthly to verify participants’ receipt of counseling and prescriptions for cessation pharmacotherapy. We anticipate that some participants may be co-prescribed varenicline or bupropion with NRT; we will document receipt of non-NRT medications by self-report at each visit and verify this information through the EHR. This approach has been used successfully in a prior study of contingency management for smoking cessation among PEH [24]. We will extract data from the EHR patient registry of enrolled patients monthly to identify referrals for clinic-provided counseling and pharmacotherapy. If a patient is not receiving treatment, study staff will message their PCP from within the EHR to remind them to provide smoking cessation treatment.

### CM intervention

We will ask all participants to choose a quit date within 7 days of providing consent [22, 24, 25, 59, 60] and meet study staff at the study site on the day of the quit attempt. In addition to usual cessation care, intervention participants with CO-verified abstinence will receive a contingency management incentive payment via gift cards redeemable in national retail chains according to a pre-defined schedule. Gift card amounts and assessment frequencies are based on prior studies among individuals experiencing homelessness or substance use disorders [22, 24, 25, 30, 59] and are expected to be effective in promoting smoking cessation. The schedule of abstinence incentive payments is shown in Table 1. Gift card amounts will begin at $13.00 and increase by $0.50 for each negative CO specimen throughout the first 6 months of the program (maximum of $25.00 per specimen), for a total of 25 specimens collected through week 24 (Table 1). Given that visit frequency decreases over time, an incentive payment schedule that increases with each visit over time is especially important to maintain motivation. The total amount that participants can earn under the contingent incentive program through week 24 is $475, an amount comparable to what has been used in prior studies [22–25]. Test results indicative of smoking (i.e., CO >5 ppm) or missed visits will lead to resetting the gift card amount back to the initial value (i.e., $13.00). However, two consecutive negative tests will restore the value back to the highest level previously achieved [22, 24, 25, 59, 60].

**Table 1:** Escalating contingency management schedule for intervention group*.

	Visit frequency	# of visits	Intervention group potential earnings	
			Total for time period	Average per visit
**Week 1**	Daily	7 ($13.00–16.00)	$101.50	$14.50
**Weeks 2–4**	Twice weekly	6 ($16.50–19.00)	$106.50	$17.75
**Weeks 5–13**	Weekly	9 ($19.50–23.50)	$193.50	$21.50
**Weeks 14–24**	Monthly	3 ($24.00–25.00)	$73.50	$24.50
**Total (6 months)**		**25**	**$475**	

*Incentive payment begins @ $13.00 and increases by $0.50 for each consecutively negative CO sample (<5 ppm) to a maximum of $25.00 for the last sample.

#### Control group incentives

Participants in the control group will receive a fixed incentive of $5 for attending each of 25 abstinence assessment visits for the first 6 months of the study.

#### Follow-up time periods

**Fig 1:**
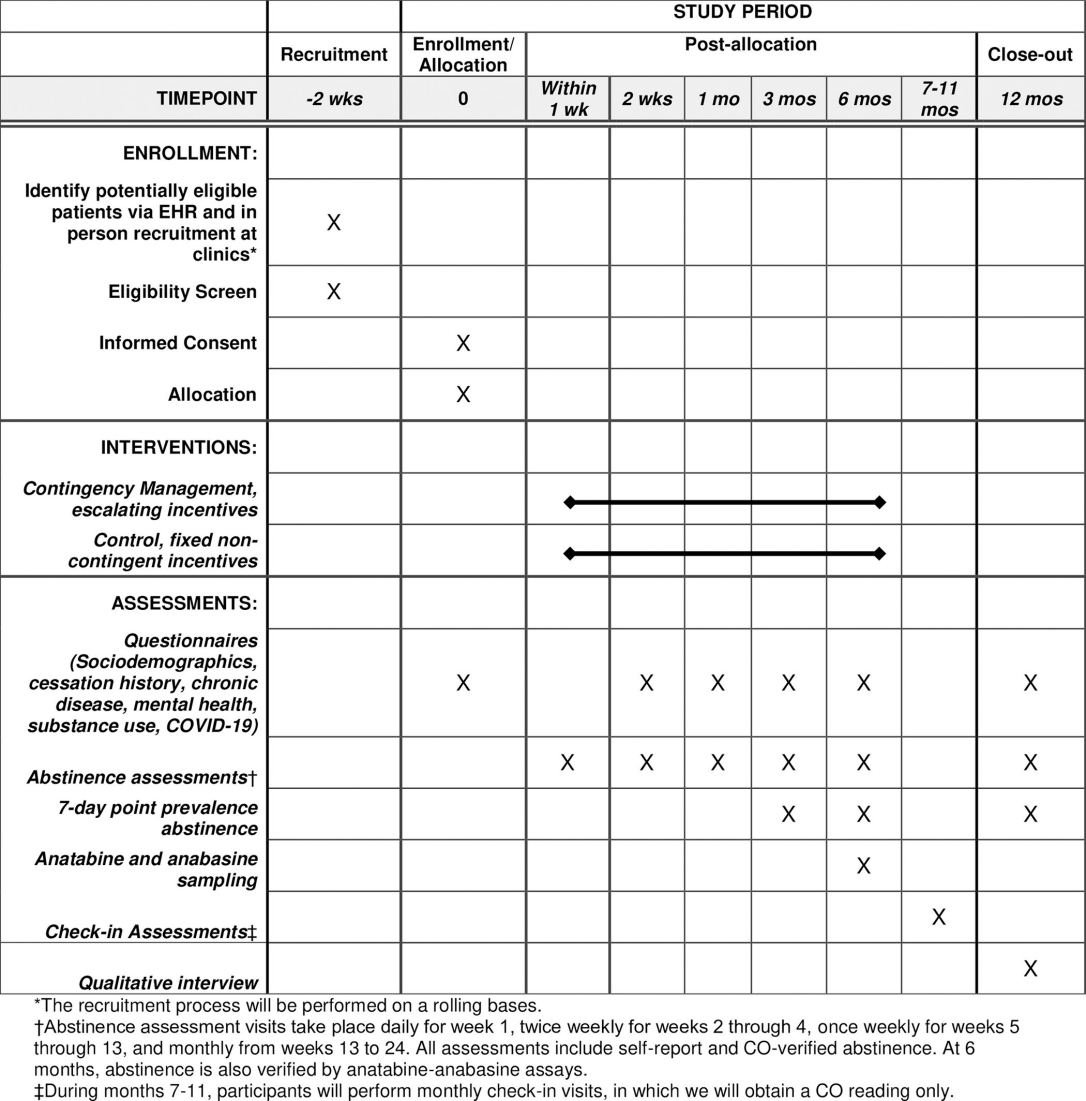
Schedule of enrollment, intervention, and assessments.

The duration of the CM intervention is 6 months, with an additional 6 months of follow up after the last intervention visit. We will ask intervention and control group participants to complete questionnaires at enrollment, 2 weeks, 1 month, 3 months, 6 months, and 12 months. Abstinence assessment visits—where participants self-report smoking abstinence and provide an expired carbon monoxide sample—will take place daily for the first 7 days (week 1), twice weekly for the next 3 weeks (until 1 month post enrollment), once weekly for the next 8 weeks (until 3 months post enrollment), and then monthly through 6 months of follow up. This follow-up schedule is based on assessment frequencies from prior studies among PEH or those with substance use disorders [22, 24, 25, 30, 59]. The abstinence assessment visit schedule will be identical for both the intervention and control groups. All participants will receive incentivized monthly check-in assessments during months 7–11 to ensure retention in the study through the last study visit at 12 months follow-up. A complete schedule of enrollment, intervention, and assessments is presented in Fig 1, which has been adapted from the SPIRIT figure [61].

### Participant retention

We will ask participants to provide and update contact information at each visit. If a participant does not present for their scheduled visit, study staff will call the participant, as well as any contacts they have provided, visit the places the participant reported that they spend time, or contact the clinic staff (study outreach). Between the 6- and 12-month assessments, we will ask and incentivize participants ($5 per visit) to check in monthly (i.e., check-in assessments). During the study period, if a participant does not show up to a follow-up visit, staff will attempt to reach the participant three times to schedule a make up visit. Participants will only be designated as “lost to follow up” when they fail to complete all of their scheduled visits for the study period. We have used these procedures successfully in prior studies that have had a similar frequency of visits and length of follow up [40–47].

#### Reimbursements

We will reimburse all participants $20 for completing the enrollment questionnaire, $15 each for questionnaires at 2 weeks, 1 month, and 3 months, and $25 each for questionnaires at 6 months and 12 months [24]. Between 6 and 12 months, all participants will also receive $5 for checking in at each monthly visit and providing a CO specimen. Thus, the total amount that all participants could earn for 1 year of participation exclusive of contingency management is $145. Intervention group participants could earn as much as $620 inclusive of contingency management ($145+$475), whereas control group participants could earn as much as $270 inclusive of the abstinence assessment visits ($145+$125).

### Quantitative measures

At the enrollment, 2-week, 1-month, 3-month, 6-month, and 12-month follow-up visits, participants will complete an interviewer-administered questionnaire in Research Electronic Data Capture (REDCap), a secure HIPAA-compliant online data collection platform [62, 63].

#### Sociodemographic, residential history, and health status

We will obtain information on age, sex assigned at birth, current gender identity, race/ethnicity, education, monthly income from all sources, health insurance, and health status [64]. For residential history, we will ask where participants stayed the previous night (e.g., unsheltered, emergency shelter, transitional shelter, being doubled up with friends/family, single room occupancy hotel, supportive housing), the length of time they stayed there, whether they were continuously homeless in the past year, and the number of episodes of homelessness in the past 3 years [54].

#### Nicotine dependence and tobacco cessation history

We will administer the Fagerstrom’s test for nicotine dependence [65]. We will collect cessation history, including intention to quit, quit attempts in the past year, length of the last quit attempt, and use of cessation aids during the last quit attempt (e.g., medications, telephone quit line).

#### Alternative tobacco and nicotine product use

We will obtain information on lifetime use and use in the past 30 days of non-cigarette tobacco and nicotine products, including electronic cigarettes, cigars, little cigars, smokeless tobacco, hookah/waterpipe, cannabis, and blunts.

#### Chronic diseases

We will ask participants whether they have received a diagnosis of liver, renal, or cardiovascular disease, hypertension, diabetes, cancer, chronic obstructive pulmonary disease, or HIV [66].

#### Mental health

We will screen for depression using the 10-item Center for Epidemiologic Studies Depression Scale, [67] anxiety using the 7-item Generalized Anxiety Disorder-7 Scale (GAD-7), [68] post-traumatic stress disorder using the Primary Care PTSD Screen for DSM-5 (PC-PTSD-5), [69] and urban stress using the Urban Life Stressors Scale [70].

#### Alcohol use, substance use, and use disorders

We will administer the Alcohol, Smoking and Substance Involvement Screening Test version 3.0 (WHO-ASSIST) [71–73]. In order to assess the volume of alcohol consumed, we will administer the consumption questions from the Alcohol Use Disorders Identification Test (AUDIT-C) [74, 75].

#### Experiences related to the COVID-19 pandemic, maltreatment, and subsistence needs

We will ask participants how the COVID-19 pandemic impacted them and their tobacco use (i.e., change in tobacco use and motivation to quit) [76]. Participants will be asked to report whether they experienced difficulty meeting subsistence needs such as food, utilities, medications, healthcare, phone, clothing, childcare, or anything else in the past 12 months [77]. Because racism and discrimination are central to experiences of homelessness and also related to tobacco use, we will assess these experiences using the Everyday Discrimination Scale [78].

### Qualitative measures

At the 12-month follow-up visit, we will inform and invite participants to take part in a qualitative study about their experiences with the CM smoking cessation trial. We will select the first 15 participants who agree to enroll. We will use an open-ended interview protocol to explore participant perceptions regarding the CM payment amounts and their perceived efficacy in motivating any smoking abstinence and/or long-term abstinence. We will also examine whether factors such as alternative forms of tobacco or treatment for substance use influenced cessation behaviors.

### Feasibility and acceptability outcomes

Table 2 shows the feasibility goals defined for this study, which we will assess using a combination of qualitative and quantitative methods. We specified goals for participants screened and recruited per month, percent of eligible participants randomized, fidelity of the CM payment procedure, study retention, and participant compliance with data collection procedures. Our threshold estimates for feasibility outcomes are based on findings from prior studies [24, 25]. For example, for the outcome of adherence to the CO assessment visits, a prior study showed that 78% of participants attended half of the assessment visits. Another study showed that, on average, participants provided a CO reading for 60% of the total possible assessment visits [24, 25]. Our goal aggregates these data and is set at 75% of participants attending at least 60% of visits.

**Table 2:** Feasibility and acceptability outcomes.

Outcomes	Measure	Goal defined as acceptable to support feasibility
Screening	# opting out, # screened	40 screened per month
Recruitment	# enrolled per month	8/month for 12 months
Randomization	Proportion eligible who are randomized	90% randomized to intervention and control arms [24]
Fidelity	Observations of incentive payment delivery	Incentive payments earned are delivered within 2 days of the visit by study staff
Adherence	Protocol adherence to the CO assessment schedule Attendance to counseling sessions Adherence to NRT among those prescribed NRT	75% of those enrolled will provide at least 15 out of 25 (60%) requested CO samples at assessment visits [24, 25] 75% will attend at least 6 out of 12 clinical care counseling sessions during the treatment duration [25] 75% will use NRT for at least half of the 6-month treatment duration [25]
Study Retention	Proportion of the sample retained as a result of retention procedures	2 weeks (85%), 3-month (85%), 6-month (75%), and 1 year (75%) retention rates
Assessment	Proportion who completed all questionnaires	75% will complete all questionnaires
Acceptability	Questionnaire and qualitative interviews with staff and patients	75% satisfied with the overall intervention

We will assess the following quantitative process outcomes monthly through the EHR, study records, and patient self-report: current smoking status, number of counseling sessions attended, number of NRT doses prescribed, number of days NRT used (by self-report), total number of follow-up visits attended, number and amount of incentive payments received.

Using data including notes/memos from staff during the study and in-depth structured interviews, we will qualitatively explore patient perceptions of the trial, contingency management, and the influence of alternative tobacco forms or treatments for substance use on cessation behaviors.

### Study outcomes

We also will assess the feasibility and acceptability of collecting abstinence outcomes. At each of the 25 scheduled abstinence assessment visits, participants will provide a CO sample and complete a short questionnaire on cigarette consumption, quit attempts since the last visit, the length of the last quit attempt, and use of medications and services. Primary outcome measures will include: 1) the proportion of participants who achieve biochemically-verified point prevalence abstinence at 6 months; 2) the median number of carbon monoxide (CO) negative samples per participant at 6 months; 3) the median total number of counseling sessions attended as part of usual cessation care per participant at 6 months; 4) the proportion of the sample retained as a result of retention procedures over time (up to 12 months). Biochemically-verified 7-day point prevalence abstinence will be defined as 1) reporting not smoking a single cigarette in the past 7 days, not even a puff, 2) having CO levels ≤5 ppm, and 3) urinary anatabine and anabasine (i.e., metabolites of tobacco) <2 ng/ml [79] (analyzed by the UCSF Cancer Center Tobacco Biomarkers Core [80]). Urinary anatabine and anabasine are tobacco alkaloids that are useful as confirmatory biomarkers to verify abstinence among patients who are using NRT for smoking cessation and for whom urinary cotinine would not be appropriate [79].

Secondary outcomes will include 7-day point prevalence abstinence at 2 weeks, 3 months and 12 months of follow up (i.e., 6 months after incentives stop), as well as prolonged abstinence, defined as participants 1) not smoking a single cigarette since their last visit, and 2) having CO levels ≤5 ppm from baseline to 3 months and baseline to 6 months [81]. We will assess the feasibility and acceptability of measuring prolonged abstinence at 6 months, as well as cigarette consumption for those unable to quit between the treatment groups.

### Ethics and dissemination

We will communicate any important protocol modifications to the IRB and the UCSF Helen Diller Family Comprehensive Cancer Center. We will document any adverse events experienced by participants in the study Case Report Forms and report them to the IRB and collaborators in accordance with all applicable institutional and regulatory requirements.

In addition to scientific publications, we expect to disseminate our findings at regional and national academic and public health conferences (e.g., Society for Research on Nicotine and Tobacco, Tobacco-Related Disease Research Program, or the Society for the General Internal Medicine), as well as conferences focused on policy-/service-related issues among populations experiencing homelessness (National Health Care for the Homeless Council). Authorship eligibility will be based on International Committee of Medical Journal Editors (ICMJE) guidelines. Other potential avenues for disseminating study results include the San Francisco County Departments of Public Health and Homelessness and Supportive Housing, as well as homeless shelters and other service providers serving homeless adults.

### Data management and storage

We will build all questionnaires on REDCap. For secure data entry and management, we will use an iPad to enter questionnaire responses directly into REDCap, which will be hosted by UCSF and maintained by the San Francisco Coordinating Center (SFCC). Study staff will review data periodically to evaluate the quality of the data. All electronic study data files will be stored in UCSF Box on a secure server. All servers are regularly backed up off-site.

We will link patient identifiers to unique study identification numbers, and we will store the identifiers in files that are kept separate from other study data, accessible only to relevant members of the research team. Qualitative data from interviews will not include any personal identifying information. We will store the transcripts on our secure server. We will password protect the study computer and store it in a locked filing cabinet in the PI’s office. Only relevant study staff will have access to study data. There will be no paper records. We will not store data at the clinic sites. We will destroy all identifying information within one year after study completion.

### Planned analyses

#### Quantitative data analysis

We will assess feasibility and acceptability outcomes, comparing them to predefined goals for each criterion listed in Table 2. Outcomes meeting or exceeding these goals will suggest a reasonable level of feasibility and acceptability for the corresponding aspects of study procedures. Any sub-threshold finding would suggest that remedial modifications to study procedures and/or design would be required prior to moving forward with a full-scale RCT. We will examine descriptive statistics of the primary/secondary clinical outcomes and explore their associations with relevant biological variables such as sex, age, and race/ethnicity. If the compliance data meets feasibility criteria and participants endorse acceptability, then we will explore differences in clinical outcomes between the two study arms.

#### Qualitative data analysis

Qualitative data can provide insights into the utility of the intervention and clinical trial procedures beyond what is learned in quantitative data. We will audio-record interviews and transcribe the recordings. We will import the documents into the Atlas.ti.v8 [82] qualitative analysis package to facilitate data management. Study staff and the principal investigator will analyze this data using a directed content analysis approach, [83] as we have done in prior studies [51, 84–86]. We will ‘code’ each transcript by labeling or marking text segments with the appropriate theme. We will re-read these text segments and develop more fine-grained codes. We will enter these new codes into Atlas.ti.v8 and we will examine the text referring to a particular code for closer analysis. We will repeat this process for all categories and domains of interest, until all the transcript data have been given fine-grain codes. We will resolve discrepancies in assignment or description of codes through discussion and consensus.

### Status and timeline of the study

At the time of this manuscript submission, the trial is actively enrolling participants. Recruitment began in October 2021, and we have enrolled 45 participants to date. We have recruited 33 participants from Site 1 and 3 participants from Site 2. We plan to continue recruitment from Site 2 and begin recruitment from Site 3 in October 2022. We expect to continue recruitment until we reach our sample size target of 90, whichever occurs first. We expect to complete 12 months of follow-up data collection by December 2023.

## Discussion

Mental health and substance use disorders among people experiencing homelessness, combined with poor access to healthcare, contribute to poor health outcomes [8] and add to the importance of addressing tobacco use in this vulnerable population. While prior efforts have focused on guideline-recommended cessation care—behavioral counseling and pharmacotherapy [12–16]—adjunctive interventions to cessation care could help PEH initate and sustain quit attempts. CM provides external motivation to engage in a health behavior. This in turn may facilitate smoking cessation by increasing self-efficacy and encouraging the use of cessation medications [87].

Although several studies have demonstrated feasibility of short-term contingency management, [22–25] studies exploring sustainable ways to promote long-term abstinence are lacking. The proposed protocol addresses this gap by incentivizing and tracking tobacco abstinence for up to 6 months.

Our trial has several strengths. First, the pragmatic nature of this pilot trial will allow us to assess the feasibility of an intervention integrated within clinical practice settings. If feasible, the intervention could be implemented at other safety-net clinics with relative ease. A larger-scale, pragmatic RCT would then improve generalizability of findings to other safety-net practice settings. Second, our methodology innovatively leverages the EHR to recruit participants, establish a registry of enrollees to obtain information on cessation services utilization, and communicate with providers to optimize protocol adherence and consistency. Through the EHR, we will be able to track participant receipt of various cessation interventions in addition to missed opportunities, mitigating the need to collect this data from individual participants. While the existence of an EHR registry is sophisticated, it could be easily developed in clinics using EHR systems such as Epic and could also serve dual purpose by helping clinics meet Quality Incentive Program metrics [88]. Furthermore, the EHR infrastructure will facilitate the scalability of a full-scale RCT. Lastly, our study employs intensive outreach procedures specifically tailored to the study population to maximize recruitment and retention, which include: obtaining multiple forms of contact, conducting study outreach to visit participants at sites where they spend time, contacting clinical staff to locate participants, incentivizing check-in visits between months 6 and 12 after CM payments have stopped, and incentivizing participants to refer other patients to the study [40–47].

One common criticism of contingency management is that the intervention effects may wane after contingency management is terminated. We hope to circumvent this by offering contingency management for up to 6 months—which is 3 months longer than most contingency management interventions in the general population [59] and 5 months longer than those among PEH [22–25]. Those participants who achieve abstinence for 6 months are less likely to experience waning of intervention effects. We will evaluate the extent to which abstinence at 6 months is sustained at 12 months of follow up, 6 months after termination of incentives.

This pilot pragmatic RCT will advance our understanding of the feasibility and acceptability of extended CM interventions for PEH. If found to be feasible, acceptable, and subsequently efficacious in a large-scale RCT, the extended duration CM intervention has the potential to reduce tobacco-related health disparities by increasing long-term abstinence among this vulnerable population.

## Supporting information

S1 Checklist SPIRIT 2013 checklist: Recommended items to address in a clinical trial protocol and related documents*. (DOC)

S1 File Approved protocol. (DOCX)

S2 File Baseline survey. (PDF)

S3 File Follow-up survey. (PDF)

S4 File Smoking abstinence assessment. (PDF)
