# Supplementary material for: Contingency management to promote smoking cessation in people experiencing homelessness: Leveraging the electronic health record in a pilot, pragmatic randomized controlled trial
Source: PLoS One. 2022 Dec 16;17(12):e0278870. doi: 10.1371/journal.pone.0278870 (PMC9757562; doi:10.1371/journal.pone.0278870)
Supplement: S1 File — (DOCX) [file pone.0278870.s002.docx]

Randomized trial of a contingency management smoking cessation intervention for homeless adults

Protocol Number: CC #MVijayaraghavan-B

Protocol Version Number: 3

Protocol Version Date: 06.28.22

Study Intervention: Contingency management intervention for smoking cessation

IND Number:

NCT Number:

Principal Investigator (Sponsor-Investigator)

Maya Vijayaraghavan

University of California San Francisco

1001 Potrero Avenue, Box 1394

San Francisco, CA 94110

Telephone: 628-206-6959

E-mail: maya.vijayaraghvan@ucsf.edu

Statistician

Steve Gregorich

**Revision History**

| Version 1  Version 2 | Date 12.31.20  Date 4.20.21 |
| --- | --- |

# Protocol Signature Page

1. I agree to follow this protocol version as approved by the Institutional Review Board (IRB).
2. I will conduct the study in accordance with Good Clinical Practices (ICH-GCP) and the applicable IRB, ethical, federal, state, and local regulatory requirements.
3. I certify that I, and the study staff, have received the required training to conduct this research protocol.
4. I agree to maintain adequate and accurate records in accordance with IRB policies and federal, state and local laws and regulations.

| **UCSF Principal Investigator**  Maya Vijayaraghavan |  |  |
| --- | --- | --- |
| Printed Name |  | 06.28.22 |
| Signature |  | Date |

# Abstract

| Title | Randomized trial of a contingency management smoking cessation intervention for homeless adults |
| --- | --- |
| Study Description | In this pilot randomized controlled trial (RCT), we will (1) adapt a known-efficacious extended CM cessation intervention to a novel population and setting, with the ultimate goal of increasing long-term abstinence among homeless adults seeking care in safety net health clinics, (2) develop a corresponding RCT protocol, and (3) conduct a pilot RCT to assess the feasibility and acceptability of the RCT. If the pilot RCT is feasible and acceptable, we will test the adapted intervention in a subsequently-funded, full-scale randomized controlled trial. |
| Study Intervention | In this study, we will test a contingency management intervention for carbon monoxide-verified smoking abstinence for people experiencing homelessness. |
| Study Population | The proposed study aims to enroll 90 homeless participants who are representative of the racial/ethnic and gender diversity of the homeless population in San Francisco. There are no exclusion criteria for this study based on gender, race, or ethnicity. We estimated our targeted enrollment based on the point-in-time count of the homeless population that occurred in 2010 in San Francisco. In 2019, there were 7499 unsheltered or publicly sheltered homeless persons in San Francisco, of whom 29% were white, 37% were black, 5% were American Indian/Alaskan Native, 5% were Asian, 2% were Native Hawaiian/Pacific Islander, 18% were Hispanic/Latino, 22% were mixed race, 35% were women, 4% identified as transgender, 1% as genderqueer/gender non-binary, and 1% as another gender. |
| Primary Objective | The primary objective is to assess feasibility and acceptability of:   - measuring biochemically-verified point prevalence abstinence at 6 months follow-up. - adhering to the protocol. - gathering information on the number of visits attended. - the retention protocol. |
| Secondary Objectives | - Assess feasibility and acceptability of measuring biochemically-verified 7-day point months follow-up at 3 months. - Assess feasibility and acceptability of measuring prolonged abstinence at 3 months and 6 months follow-up. - Assess feasibility and acceptability of measuring 12 point prevalence abstinence |
| Recruitment Methods | Recruitment methods include flyers, in-clinic recruitment, and via email to healthcare providers. |
| Sample Size | We plan to enroll 90 participants. |
| Duration of Study Participation | The intervention duration is 1 year. |
| Unique Aspects of this Study | This study will be the first to evaluate an extended contingency management intervention for smoking cessation for people experiencing homelessness. |

| List of Abbreviations *Add/Remove abbreviations as applicable to the study protocol* | |
| --- | --- |
| AE | adverse event |
| CRF | case report form |
| CTCAE | Common Terminology Criteria for Adverse Events |
| CTMS | Clinical Trial Management System |
| DSMC  DSMP | Data and Safety Monitoring Committee  Data and Safety Monitoring Plan |
| GCP | Good Clinical Practice |
| HDFCCC | Helen Diller Family Comprehensive Cancer Center |
| HIPAA | Health Insurance Portability and Accountability Act |
| ICF | informed consent form |
| ICH | International Conference on Harmonization |
| IRB  PHP | Institutional Review Board  Positive Health Program |
| PRC  RFPC  TWUHC  ZSFG | Protocol Review Committee (UCSF)  Richard H. Fine People’s Clinic  Tom Waddell Urban Health Clinic  Zuckerberg San Francisco General Hospital |

| Table of Contents |
| --- |
| [Protocol Signature Page 3](#_Toc50989995)  [Abstract 4](#_Toc50989997)  [List of Abbreviations 6](#_Toc50989998)  [Table of Contents 7](#_Toc50989999)  [1 Introduction 10](#_Toc50990000)  [1.1 Background on <<condition, symptom, behavior, or other primary study focus>> 10](#_Toc50990001)  [1.2 Background on <<study intervention>> 10](#_Toc50990002)  [1.3 Study Rationale 10](#_Toc50990003)  [1.4 Risk/Benefit Assessment 10](#_Toc50990004)  [2 Study Objectives and Endpoints 13](#_Toc50990005)  [2.1 Primary Objective 14](#_Toc50990006)  [2.2 Secondary Objective(s) 15](#_Toc50990007)  [2.3 Exploratory Objective(s) 15](#_Toc50990008)  [3 Study Design 15](#_Toc50990009)  [3.1 Characteristics 15](#_Toc50990010)  [3.2 Sample Size 16](#_Toc50990011)  [3.3 Primary Completion 16](#_Toc50990012)  [3.4 Study Completion 16](#_Toc50990013)  [4 Selection and Enrollment of Participants 16](#_Toc50990014)  [4.1 Eligibility Criteria 16](#_Toc50990015)  [4.1.1 Inclusion Criteria 16](#_Toc50990016)  [4.1.2 Exclusion Criteria 17](#_Toc50990017)  [4.2 Recruitment Methods 17](#_Toc50990018)  [4.3 Inclusion of Women and Minorities 18](#_Toc50990019)  [4.3.1 Eligibility of Women and Minorities 18](#_Toc50990020)  [4.3.2 Recruitment of Women and Minorities 18](#_Toc50990021)  [4.4 Inclusion Across the Lifespan 18](#_Toc50990022)  [4.4.1 Age Range of Participants 18](#_Toc50990023)  [4.4.2 Study Design/Recruitment Considerations Related to Age Groups 18](#_Toc50990024)  [4.5 Participant Registration 18](#_Toc50990025)  [4.6 Randomization/Assignment to Intervention 18](#_Toc50990026)  [4.7 Blinding 19](#_Toc50990027)  [5 Study Intervention 19](#_Toc50990028)  [5.1 Administration and/or Delivery of Study Intervention 19](#_Toc50990029)  [5.2 Interventionist Training and Tracking 20](#_Toc50990030)  [5.3 Modifications to Administration of the Intervention and/or Supportive Care 20](#_Toc50990031)  [5.4 Adherence Assessment 21](#_Toc50990032)  [5.5 Concomitant Therapy 21](#_Toc50990033)  [5.5.1 Allowed Therapy 21](#_Toc50990034)  [5.5.2 Required Therapy 21](#_Toc50990035)  [5.5.3 Prohibited Therapy 22](#_Toc50990036)  [5.6 Participant Discontinuation/Withdrawal from the Study 22](#_Toc50990037)  [5.7 Lost to Follow-up 22](#_Toc50990038)  [6 Study Procedures and Assessments 23](#_Toc50990039)  [6.1 Schedule of Activities 24](#_Toc50990040)  [6.2 Study Procedures and Assessments 25](#_Toc50990041)  [6.2.1 Screening Period / Visit -1 (Day -# to Day 1) 25](#_Toc50990042)  [6.2.2 Study Intervention Period 25](#_Toc50990043)  [6.2.3 End of Study Intervention / Visit X (Day # +/- #) 26](#_Toc50990044)  [6.2.4 Follow-up 26](#_Toc50990045)  [7 Reporting and Documentation of Results 26](#_Toc50990046)  [7.1 Measures and Instruments 26](#_Toc50990047)  [8 Adverse Events and Serious Adverse Events 27](#_Toc50990048)  [8.1 Definition of Adverse Event 27](#_Toc50990049)  [8.2 Definition of Serious Adverse Event 27](#_Toc50990050)  [8.3 Classification of Adverse Events 28](#_Toc50990051)  [8.3.1 Severity 28](#_Toc50990052)  [8.3.2 Attribution 28](#_Toc50990053)  [8.3.3 Expectedness 28](#_Toc50990054)  [8.4 Adverse Events Monitoring 28](#_Toc50990055)  [8.5 Follow up of Adverse Events 28](#_Toc50990056)  [8.6 Documenting and Reporting of Adverse Events 28](#_Toc50990057)  [9 Statistical Considerations 28](#_Toc50990058)  [9.1 Sample Size Considerations 28](#_Toc50990059)  [9.1.1 Sample Size and Power Estimate 28](#_Toc50990060)  [9.1.2 Randomization and Blinding 29](#_Toc50990061)  [9.1.3 Stratification Factors 29](#_Toc50990062)  [9.1.4 Accrual Estimates 29](#_Toc50990063)  [9.2 Interim Analyses and Stopping Rules 29](#_Toc50990064)  [9.3 Statistical Analysis Plans 29](#_Toc50990065)  [9.3.1 Analysis Populations 29](#_Toc50990066)  [9.3.2 Primary Analysis (or Analysis of Primary Endpoints) 29](#_Toc50990067)  [9.3.3 Secondary Analysis (or Analysis of Secondary Endpoints) 30](#_Toc50990068)  [9.3.4 Exploratory/Correlative Analysis/Assessments 30](#_Toc50990069)  [10 Study Management 30](#_Toc50990070)  [10.1 Pre-study Documentation 30](#_Toc50990071)  [10.2 Institutional Review Board Approval 30](#_Toc50990072)  [10.3 Informed Consent 30](#_Toc50990073)  [10.4 Changes in the Protocol 30](#_Toc50990074)  [10.5 Case Report Forms (CRFs) 30](#_Toc50990075)  [10.6 Record Retention 31](#_Toc50990076)  [10.7 Publications 31](#_Toc50990077)  [11 References 32](#_Toc50990083) |

# Introduction

## Background

Cigarette smoking is exceedingly common among people experiencing homelessness, with a prevalence of 70% compared to 13.7% in the general population.^1,2^ Severe mental illness and substance use disorders are common among homeless adults and are risk factors for smoking and for poor smoking-related health outcomes.^3^ Smoking-induced cardiovascular diseases and cancers are the leading causes of morbidity and mortality among homeless adults.^4-8^

Homeless adults make quit attempts as frequently as the general population, but they are less successful at quitting completely.^9-11^ The quit ratio (former/ever smokers), a measure of successful quitting, among homeless adults is between 9% and 13%,^11,12^ almost 5 times lower than the general population (~61%).^2^ Clinical trials of behavioral counseling and pharmacotherapy—the standard care for smoking cessation—have failed to demonstrate substantial long-term abstinence among homeless adults.^13-17^ Long-term abstinence, defined as abstinence for 6 months or more, is a strong predictor of successful cessation.^18^ Behavioral counseling and pharmacotherapy alone may be insufficient to promote long-term abstinence, which is necessary to increase cessation rates among this population.

Cessation interventions not only need to be efficacious in clinical trials, but also scalable to practice settings.^19,20^ Although there have been randomized controlled trials (RCT) of cessation interventions for homeless adults, these trials have not been tested in practice-based settings. The majority of homeless adults in the U.S. seek health care in federally-funded, safety net community health centers. Safety net health clinics that provide care to homeless adults could bring cessation interventions to scale.

Research is needed to 1) identify interventions that can increase the effect of behavioral counseling and pharmacotherapy on long-term abstinence (≥6 months) among homeless adults; and 2) determine how such interventions can be brought to scale in safety net clinics serving homeless adults. As a step toward answering those questions, we will pilot test a RCT of a contingency management smoking cessation intervention for homeless adults seeking care in a safety net health clinic.

## Background on the intervention

Contingency management (CM), a behavior change strategy that reinforces positive health behaviors with incentives (e.g., cash), has been used in clinical trials to reduce tobacco and substance use in the general population.^21-24^ Smokers who abstain receive modest incentives; these incentives reinforce the healthy behavior.^21^ There have been clinical trials that have incorporated CM for the treatment of tobacco dependence among low-income pregnant women and persons with mental health or substance use disorders.^23,25-29^ Recent research, including uncontrolled pilot studies ^30,31^ and two recent pilot RCT,^32,33^ has demonstrated the feasibility of short-term CM (8 weeks or less) for smoking cessation among homeless adults. Some evidence suggests that extended CM, defined as CM for ≥12 weeks, can be efficacious in promoting long-term abstinence for ≥6 months.^21,34-39^ There are no feasibility trials evaluating extended CM to promote long-term abstinence among homeless adults, and none that have been adapted to safety net health clinics.

In this pilot randomized controlled trial (RCT), we will (1) adapt a known-efficacious extended CM cessation intervention to a novel population and setting, with the ultimate goal of increasing long-term abstinence among homeless adults seeking care in safety net health clinics, (2) develop a corresponding RCT protocol, and (3) conduct a pilot RCT to assess the feasibility and acceptability of the RCT. If the pilot RCT is feasible and acceptable, we will test the adapted intervention in a subsequently-funded, full-scale randomized controlled trial.

## Study Rationale

This study is being done to evaluate the feasibility and acceptability of an extended intervention for contingency management for smoking cessation for homeless adults. Some evidence suggests that extended CM, defined as CM ≥12 weeks (range 3 months to 1 year), among smokers with substance use disorders, can increase abstinence rates and efficacy of long-term abstinence for 6 months or more. In this pilot study, we will adapt a known-efficacious extended CM intervention, with provision of incentives for 6 months to promote abstinence for ≥ 6 months, to homeless adults engaged in care in a safety net health clinic.

## Risk/Benefit Assessment

**Potential risks.** There may be a potential for loss of privacy around homelessness status, which could affect participant’s standing in society. There may be a potential for loss of privacy relating to the information asked in the questionnaires, particularly around issues on mental health disorders, substance use disorders, and previous history of homelessness. Loss of privacy or confidentiality–particularly around mental health, illicit substance use, HIV status, impaired function, and information contained in medical records–could result in embarrassment or social marginalization. In the case of illicit substance use, loss of privacy or confidentiality could result in legal consequences. Because we will ask participants to consent to keep on file their name and contact and tracking information, there may be a potential for loss of confidentiality. Participants may experience symptoms of nicotine withdrawal from reducing consumption or quitting smoking. These symptoms may include irritability, fatigue, headaches or cravings for cigarettes. Participants may also experience hunger as a result of reducing consumption or quitting smoking. There is a possibility that participants may experience side effects from medications for cessation. There is a low risk of study participants experiencing fatigue and/or boredom during study visits. In addition to the above, we note other risks that may be relevant to our study population. Given the significantly low economic status of our study participants, study incentives and contingency management may be coercive. We believe that the risk of these events is low given the measures we will put in place to minimize these risks (see below).

**Procedures for protecting against risks.**

1. Procedures for protecting against loss of privacy. Although we collect basic information from homeless participants on socio-demographics and factors that can influence smoking cessation such as mental health disorders or substance use disorders, it will be kept to a minimum to minimize any psychological risk. All interviews will be conducted in a way that is formative without breaching the confidentiality of individuals participating in the study. We will ensure participants that responses to the questionnaires, in-depth, semi-structured interviews or focus groups will not affect their eligibility for receiving services. We will assure participants who decide not to participate that their decision would not affect their ability to seek services. Participation in this study is voluntary.
2. Procedures for protecting against loss of confidentiality. There is a potential for loss of confidentiality, however we will take measures to minimize this risk. Each participant will have a unique alpha-numeric identification (ID) number. We will use the UCSF REDCap secure database to enter personal identifying information and to create questionnaires for each of the studies. Identifying information collected from participants for tracking purposes will be stored separately from research data and will not be associated with the participants’ unique study ID number. All questionnaire data will be entered in real-time using an iPad through a secure VPN network. In-depth, semi-structured interviews and focus groups will be audio-recorded. These recordings and their transcriptions will be stored on our secure server at UCSF. The server where data will be stored is backed up nightly. We will periodically go through the study data to make sure there is no personal identifying information. Only study staff will have access to study data. For added protection of confidentiality, we will apply for Certificate of Confidentiality from the National Cancer Institute.
3. Procedures for protection against legal risk to participants. The structured interview will include questions about illegal behavior (e.g., the use of illicit drugs). Procedures explained above for protecting privacy of individuals and confidentiality of data will minimize the legal risk to participants. For added protection of confidentiality of data, we will apply for a Certificate of Confidentiality from the National Cancer Institute. Trained study staff will inform participants of legal risks during the informed consent process and will explain that participants can skip out of any interview questions they are not comfortable answering.
4. Procedures for protecting against coercion. Reimbursements may be coercive. There is controversy around whether reimbursements could be coercive or used to purchase illicit drugs when given to indigent populations participating in research. In trying to balance the need to reimburse participants for their time while limiting coercion and/or dangerous behaviors, we have set reimbursement levels comparable to those used in studies in similar populations.^18-20,22^ An alternative to cash reimbursements, we have chosen to reimburse in the form of gift certificates for grocery stores and general pharmacies.
5. Procedures for protecting against hunger. It is possible that individuals who are attempting to quit smoking may experience hunger. Should this issue arise during the intervention, we will provide clients a list of resources of free food around the city of San Francisco including food banks for food in bulk, soup kitchens for daily meals, and other free meal programs.
6. Procedures for protecting against withdrawal symptoms. Participants may experience symptoms of nicotine withdrawal from reducing consumption or quitting smoking. These symptoms include irritability, fatigue, headaches or cravings for cigarettes. We expect that participants enrolled in the clinical trial will be engaged in usual cessation care at the Tom Waddell Urban Health Clinic (TWUHC), The Richard H. Fine People’s Clinic (RFPC), or Positive Health Program Clinic (PHP), that includes one-on-one cessation counseling through behavioral counselors and NRT, dispensed on-site by a pharmacist, to prevent nicotine withdrawal and/or reduce cravings. Dosage of NRT will be adjusted according to participants’ consumption levels and based on participants’ urge to smoke. Primary care providers (PCPs) will verify and document in the EHR that there are no contraindications to receiving NRT such as having had a myocardial infarction in the 2 weeks prior to enrollment or pregnancy. HIPAA-trained study staff will access the EHR weekly to verify participants’ attendance of counseling and receipt of NRT. We anticipate that some participants may be co-prescribed varenicline or bupropion with NRT; we will document receipt of non-NRT medications by self-report at each visit and verify this information through the EHR. For participants who are unable to obtain medications, the study will provide a 12-week supply of NRT and study staff will discuss indications of use, contraindications, and potential side effects prior to use.
7. Procedures for protecting against adverse effects of cessation medications. PCPs will verify and document in the EHR that there are no contraindications to receiving NRT such as having had a myocardial infarction in the 2 weeks prior to enrollment or pregnancy. Participants who receive varenicline or bupropion from their PCPs will be asked to discuss the potential side effects with their PCPs before initiating medications, and study staff will confirm that this discussion has taken place. We will document receipt of non-NRT medications by self-report at each visit and verify this information through the EHR. At each assessment visit, study staff will assess adherence to medications and side effects of medications. We will ask participants to contact us in between visits if they experience side effects. If participants report side effects related to the use of cessation medications or if any psychiatric symptoms emerge, we will refer them to their PCPs. For serious concerns such as suicidal ideation or self-injurious behavior, we will refer them to the emergency room at the Zuckerberg San Francisco General Hospital, where the PI is an attending physician. The PI is a general internist with over 13 years of clinical experience, licensed in the state of California, and will be on call at all times to advise staff and can directly intervene with participants if needed.
8. Procedures for handling reportable conditions: During the informed consent process, participants will be informed of information that must be reported by law should it be revealed during study visits, including suicidality and homicidality. The PI will train the study staff on how to assess for symptoms of suicidality or homicidality, and the procedures to follow in the event of these occurrences. In addition, the PI will be on call at all times and will be available for consultation.
9. Procedures for protecting against fatigue or boredom: The enrollment study visit for the pilot RCT will last approximately 1 hour, and the follow-up visits at 2 weeks, 1 months, 3 months, 6 months, and 1 year will last approximately a half-hour. The in-depth, semi-structured interviews and focus group in Aims 1 and 2 will last 60 minutes to 90 minutes. Assessment visits for abstinence monitoring will last approximately 10 minutes. Regular breaks will be scheduled throughout the study visits, between structured interview instruments and assessments. Study staff will inform participants, during informed consent and at each study visit, that they can take breaks or stop participating in the study at any time.
10. Added protections for vulnerable populations: If a participant becomes incarcerated during the course of the study, they will be censored from participation during incarceration. If released from jail/prison during the study timeframe they can choose to re-enroll and a research assistant will repeat the informed consent process using procedures explained above.

**Risk benefit.** Given the minimal anticipated risks from this study, we think that proposed study has the potential to offer significant societal benefits. This study has the potential to inform development of an effective and scalable intervention to reduce tobacco use among homeless adults. In doing so, the study may inform future policies and interventions to reduce tobacco use and its associated morbidity and mortality among the diverse populations experiencing homelessness.

# Study Objectives and Endpoints

## Primary Objective

| **Primary Objective** | **Endpoint(s)** | **Time Frame** |
| --- | --- | --- |
| 1. Assess feasibility and acceptability of measuring biochemically-verified point prevalence abstinence at 6 months follow-up. | Evaluate the proportion of participants who achieve biochemically-verified 7-day point prevalence abstinence defined as participants (1) reporting not smoking a single cigarette in the past seven days, not even a puff; (2) having CO levels ≤5 ppm, and (3) anatabine/anabasine assays <2 ng/ml. | Baseline to 6 months follow-up |
| 1. Assess feasibility and acceptability of adhering to the protocol | Total number of carbon monoxide negative samples | Baseline to 6 months follow-up |
| 1. Assess feasibility and acceptability of gathering information on the number of visits attended | Total number of visits attended | 6 months |
| 1. Assess feasibility and acceptability of the retention protocol | Proportion of the sample retained as a result of retention procedures | 2 weeks, 1 month, 3 months, 6 months, and 12 months follow-up |

## Secondary Objective(s)

| **Secondary Objective** | **Endpoint(s)** | **Time Frame** |
| --- | --- | --- |
| 1. Assess feasibility and acceptability of measuring biochemically-verified 7-day point months follow-up at 3 months | Evaluate the proportion of participants who achieve biochemically-verified 7-day point prevalence abstinence defined as participants (1) reporting not smoking a single cigarette in the past seven days, not even a puff; and (2) having CO levels ≤5 ppm | Baseline to 3 months |
| 1. Assess feasibility and acceptability of measuring prolonged abstinence at 3 months and 6 months follow-up | Evaulate the proportion of participants who achieve prolonged abstinence defined as participants (1) not smoking a single cigarette since the last visit; (2) having CO levels ≤5 ppm. | Baseline to 3 months, and baseline to 6 months follow-up |
| 1. Assess feasibility and acceptability of measuring 12 point prevalence abstinence | Proportion of participants who achieve biochemically-verified 7-day point prevalence abstinence defined as participants (1) reporting not smoking a single cigarette in the past seven days, not even a puff and (2) having CO levels ≤5 ppm. | Baseline to 12 months follow-up. |

## Exploratory Objective(s)

| **Exploratory Objective** | **Endpoint(s)** |
| --- | --- |
| 1. Longest duration of abstinence | 6 months |

# Study Design

## Characteristics

This is a phase I single site RCT of an extended contingency management intervention for smoking cessation for people experiencing homelessness and who are engaged in clinical care at the Tom Waddell Urban Health Clinic (TWUHC), Richard H. Fine People’s Clinic (RFPC), or the Positive Health Program (PHP) Clinic. Participants will be recruited from the TWUHC, RFPC, and PHP, and study procedures will take place at the Civic Center Plaza or at the Zuckerberg San Francisco General Hospital (ZSFG) courtyard to minimize risk from gathering in indoor locations during the COVID-19 pandemic. We will randomize 45 participants to the intervention arm and 45 participants to the control arm. Participants will receive usual cessation care at TWUHC, RFPC, or PHP, that includes weekly behavioral counseling and pharmacotherapy. The intervention will include the provision of financial incentives contingent on biochemically-verified abstinence for up to 6 months, with a potential to earn up to $620 in contingent reinforcements for cessation. Control group participants will receive $5 for attending each abstinence assessment. Participants will be asked to choose a quit date within 7 days of providing consent, and will meet study staff at TWUHC, RFPC, or PHP on the day of the quit attempt. Participants will meet daily for the first 7 days (week 1), twice weekly for the next 3 weeks (1 month), once weekly for the next 9 weeks (3 months), and monthly until the one-year follow-up (1 year). Assessment visit schedule is identical for both groups. At each of the scheduled assessment visits, participants will provide a CO sample and complete a short questionnaire on cigarette consumption, quit attempts since the last visit, the length of the last quit attempt, and use of medications and services. Questionnaires will be built on REDCap, and administered by study staff using an iPad. At enrollment, 2 weeks, 1 month, 3 months, 6 months, and 1-year follow-up, participants will be asked to complete structured questionnaires. The final study follow-up will be at 1 year.

## Sample Size

We expect to enroll 90 participants in the RCT, with 45 randomized to control and 45 randomized to intervention. We expect to screen 40 eligible participants per month, of which we anticipate that 10 (33% participation rate) will agree to enroll, for a goal enrollment of 8 participants per month over 12 months.

## Primary Completion

The expected primary completion date is 18 months after the study opens to accrual.

## Study Completion

The expected study completion date is 2 years after the study opens to accrual.

# Selection and Enrollment of Participants

## Eligibility Criteria

### Inclusion Criteria

In order to be eligible to participate in this study, an individual must meet all of the following criteria:

1. Are 18 years or older,
2. Attend an in-person visit at the Tom Waddell Urban Health Clinic, Richard H. Fine People’s Clinic, or the Positive Health Program Clinic,
3. Meet criteria for homelessness as defined by the Homeless Emergency Assistance and Rapid Transition to Housing Act,
4. Are current smokers (smoked at least 100 cigarettes in lifetime, smoked daily in the past 7 days and at least 5 cigarettes per day, verified by expired Carbon Monoxide (CO)≥ 8 parts per million (ppm)),
5. Have an intention to quit smoking within the next six months,
6. Are attending on-site smoking cessation counseling provided by the behavioral counselors,
7. Are English proficient, and
8. Are able to provide informed consent. Patients who are interested in participating but not enrolled in counseling services will be given the opportunity to join weekly one-on-one counseling for a period of 2 weeks prior to enrollment.

### Exclusion Criteria

An individual who meets any of the following criteria will be excluded from participation in this study:

1. Are unable to take nicotine replacement therapy (e.g., pregnancy or MI within the past 2 weeks)

## Recruitment Methods

The study recruitment strategy aims to achieve representation of minority groups that reflects the demographics of the affected population in the catchment area. HIPAA-trained study staff will be present during scheduled clinic sessions to recruit patients. Prior to each clinic session, study staff will use the EHR to identify smoking patients among those scheduled to be seen. Study staff will approach patients who have screened positive for cigarette smoking during intake prior to their primary care or urgent care visits, confirm cigarette smoking status, ask whether they would be interested in hearing about a research study, and screen for basic study eligibility. Study staff will also recruit participants using flyers in the clinic waiting area, and have interested participants call study staff at the designated phone numbers. Lastly, study staff will email providers at the clinic to inform them about the study and ask them to either refer interested patients and request permission to contact potentially eligible patients for the study via telephone.

Participants from the TWUHC, RFPC, and PHP, who meet study criteria will complete enrollment procedures at the time of eligibility or within 1 week of recruitment either by telephone or in person at the Civic Center Plaza or the Zuckerberg San Francisco General Hospital courtyard, the primary study site for the study. Study activities will also take place at the RFPC and PHP, both located at the ZSFG. Study staff will describe study procedures and obtain written informed consent using the teach-to-goal method at the time of eligibility. We will conduct all in person procedures at the Civic Center Plaza or at ZSFG.

The study site has been modified from the TWUHC to the Civic Center Plaza and the courtyard outside of ZSFG because the clinics have reduced their capacity for in-person evaluation because of the COVID19 pandemic. To minimize risk of transmission, we have identified the Civic Center Plaza, an outdoor site, as an alternative study site for the study. We will follow the San Francisco Department of Public Health guidance for preventing transmission of viruses. All participants will be provided a mask if they don’t have one. We will pick a quiet spot at the plaza, and meet participants one at a time to maintain privacy and confidentiality.

## Inclusion of Women and Minorities

### Eligibility of Women and Minorities

Individuals of any sex/gender, race, or ethnicity are eligible for this study. There are no exclusion criteria for this study based on gender, race, or ethnicity. We estimated our targeted enrollment based on the point-in-time count of the homeless population that occurred in 2010 in San Francisco (See Targeted Enrollment Table). In 2019, there were 7499 unsheltered or publicly sheltered homeless persons in San Francisco, of whom 29% were white, 37% were black, 5% were American Indian/Alaskan Native, 5% were Asian, 2% were Native Hawaiian/Pacific Islander, 18% were Hispanic/Latino, 22% were mixed race, 35% were women, 4% identified as transgender, 1% as genderqueer/gender non-binary, and 1% as another gender.

### Recruitment of Women and Minorities

The study recruitment strategy aims to achieve representation of minority groups that reflects the demographics of the affected population in the catchment area.

## Inclusion Across the Lifespan

### Age Range of Participants

Individuals ages 18 and over are eligible for this study. No children under the age of 18 years of age will be included in this study of adult cigarette smokers. Eligible participants will include those who are ≥ 18 years of age. The tobacco use profile and the likely response to interventions among homeless youth smokers is sufficiently different from that of homeless adult smokers, warranting separate studies of homeless youth and adult cigarette smokers. Moreover, the proposed CM intervention is based on prior studies of adults and not youth participants.

### Study Design/Recruitment Considerations Related to Age Groups

The study design and recruitment strategy aim to achieve representation of age groups that reflect the demographics of the affected population. We will recruit participants that reflect the age and gender diversity of people experiencing homelessness in San Francisco (See eligibility criteria and inclusion of women and minorities sections above).

## Participant Registration

A written, signed, informed consent form (ICF) and a Health Insurance Portability and Accountability Act (HIPAA) authorization must be obtained before any study-specific assessments are initiated. A copy of the signed ICF will be given to the subject and a copy will be filed in the medical record. The original will be kept on file with the study records.

All participants consented to the study will be registered in OnCore®, the UCSF Helen Diller Family Comprehensive Cancer Center Clinical Trial Management System (CTMS). The system is password protected and meets HIPAA requirements.

## Randomization/Assignment to Intervention

Randomization will be stratified by recruiter and a binary nicotine dependency code determined by participant response to a single item asked during study enrollment: "How soon after you wake up do you smoke your first cigarette?" (≤30 minutes coded as 'high' dependency; >30 minutes coded as 'low' dependency; Fagerström 2003). Before initiating the study, stratum-specific sequential ID numbers will be randomly pre-assigned to experimental groups in blocks of random size. The PI will be blinded to this assignment, and only the study’s statistician (Dr. Gregorich) will have access to the linkage between ID numbers and intervention assignments. The study ID numbers and corresponding experimental group assignments will be preloaded into REDCap. When a participant is enrolled, REDCap will assign the first available ID number within the appropriate stratum. After completing the baseline interview, participants will be informed of their study ID number and their experimental group assignment. The study will randomize 45 participants to the intervention arm and 45 to the control arm (n=90).

## Blinding

In the proposed Feasibility and Acceptability RCT and the subsequent RCT, the PI will stay blinded and study staff will be responsible for the incentive payments, collecting the study data, and this information will not be shared with the PI. Any progress report prepared during the study will not reveal intervention assignments, and the study’s statistician will not share any unblinded results with the PI. We have clarified this in the protocol.

# Study Intervention

## Administration and/or Delivery of Study Intervention

**Usual smoking cessation care at TWUHC, RFPC, and PHP**. Patient participants will be required agree to attend usual smoking cessation care that is available to all clinic patients who receive clinical services at the clinic. Usual cessation care includes weekly one-on-one counseling by on-site behavioral counselors and pharmacist-dispensed NRT (i.e., transdermal patch, the gum or lozenge, the primary cessation medications in this clinical trial) covered by health insurance for at least 12 weeks.

| **Table 1: Escalating Contingency Management Schedule for Intervention Group^** | | | | |
| --- | --- | --- | --- | --- |
|  | Visit frequency | # of Visits | Intervention group potential earnings | |
|  |  |  | Total for time period | Average per visit |
| Week 1 | Daily | 7 ($13.00-16.00) | $101.50 | $14.50 |
| Weeks 2 – 4 | Bi-weekly | 6 ($16.50-19.00) | $106.50 | $17.75 |
| Weeks 5 – 13 | Weekly | 9 ($19.50-23.50) | $193.50 | $21.50 |
| Weeks 14– 24 | Monthly | 3 ($24.00-25.00) | $73.50 | $24.50 |
| Total (6 months) |  | **25** | **$ 475** |  |
| ^ Incentive payment begins @ $13.00 and increases by $0.50 for each consecutively negative CO sample (<5 ppm) to a maximum of $24.50 for the last sample. | | | | |

**Intervention.** In addition to receiving usual cessation care, CM intervention participants with CO-verified abstinence will obtain a CM incentive payment, via gift cards redeemable in national retail chains, according to a pre-defined schedule. Gift card amounts and assessment frequencies are based on prior studies among individuals experiencing homelessness or with substance use disorders,^26,30,32,33,38^ and are expected to be effective in promoting smoking cessation. We think this CM schedule is close to what will be adopted; however, our results from Aim 1 will inform the protocol. Gift card amounts will begin at US $13 and increase by $0.50 for each negative CO specimen throughout the first 6 months of the program to a maximum of $24.50 per specimen for a total of 25 specimens collected through week 24 (Table 3). An escalating schedule that increases per visit incentive payment over time is especially important in this study to maintain motivation as visit frequency decreases over time. The total amount that participants can earn under the contingent incentive program through week 24 is $620, an amount comparable to what has been used in prior studies.^30-33^ Test results indicative of smoking (i.e. CO >5ppm) or missed visits will lead to resetting the gift card amount back to the initial low value, but two consecutive negative tests will restore the value back to the highest level previously achieved. ^26,30,32,33,100^

**Control condition.** Control group participants will receive a fixed amount of $5 for attending each abstinence assessment visit and participate in usual cessation care, as described above.^32^ This approach has been used in a prior study of CM for smoking cessation among homeless adults.^32^

**Abstinence assessment schedule*.*** All other aspects of the study protocol will be identical across the two experimental groups. Participants will be asked to choose a quit date within 7 days of providing consent,^26,30,32,33,100^ and will meet study staff at TWUHC or ZSFG on the day of the quit attempt (Table 1). Participants will meet daily for the first 7 days (week 1), twice weekly for the next 3 weeks (1 month), once weekly for the next 9 weeks (3 months), and monthly until the one-year follow-up (1 year). Assessment visit schedule is identical for both groups. At each of the scheduled assessment visits, participants will provide a CO sample and complete a short questionnaire on cigarette consumption, quit attempts since the last visit, the length of the last quit attempt, and use of medications and services. Questionnaires will be built on REDCap, and administered by study staff using an iPad.

***Abstinence assessment.*** As in prior studies, ^26,30,32,33,100^to determine eligibility for CM, we will assess point-prevalence abstinence with CO monitoring, using a cut-off of CO≤ 5 parts per million (ppm).^98^ For primary outcome assessment, we will confirm abstinence among participants who self-report abstinence and meet criteria for abstinence based on CO with urinary anatabine and anabasine (i.e. metabolites of tobacco) using a cut-off of <2 ng/ml ^101^ (analyzed by the UCSF Cancer Center Tobacco Biomarkers Core ^102^). Urinary anatabine and anabasine are tobacco alkaloids that are useful as confirmatory biomarkers to verify abstinence among patients who are using NRT for smoking cessation, and for whom urinary cotinine would not be appropriate.^101^

**Data collection reimbursements*.*** We will reimburse all participants with $20 for completing the baseline questionnaire, $15 each for questionnaires at 2, weeks, 1 month, and 3 months, and $25 each for questionnaires at 6 months and 1-year follow-up. Between 6 and 12 months, all participants will receive $5 for checking in at each monthly visit and providing a CO specimen. The total amount that all participants could earn for 1 year of participation exclusive of CM is $145 and as much as $620 inclusive of CM ($145+$475).

**Clinical outcomes for the future full-scale RCT*.*** The primary outcome for the subsequent full-scale RCT will be biochemically-verified 7-day point prevalence abstinence at 6 months. Biochemically-verified 7-day point prevalence abstinence is defined as participants (1) reporting not smoking a single cigarette in the past seven days, not even a puff; (2) having CO levels ≤5 ppm, and (3) anatabine/anabasine assays <2 ng/ml. Secondary outcomes include 7-day point prevalence abstinence at 2 weeks and 3 months, and prolonged abstinence at each follow-up visit as per recent guidelines.^122^ We will also assess total number of CO-negative samples, longest duration of abstinence (i.e., total number of un-interrupted days of abstinence), total number of visits attended, and cigarette consumption for those unable to quit between the treatment groups. We will evaluate abstinence at 1 year to assess this outcome 6 months after incentives stop. In this pilot, we will assess the feasibility and acceptability of collecting each of these outcomes.

## Adherence Assessment

Using a combination of qualitative and quantitative methods, we will evaluate whether: 1) we met our screening and recruitment goals, 2) the randomization scheme worked and that participants were willing to be randomized, 3) the CM intervention was delivered with fidelity, 4) the retention procedures enabled tracking and retention, 5) the outcome assessments were valid, reliable, and complete, and 6) participants adhered to the protocol (Table 2).

| **Table 2: Feasibility and acceptability outcomes** | | |
| --- | --- | --- |
| Outcomes | **Measure** | **Threshold** |
| Screening | # opting out, # screened | 40 screened per month |
| Recruitment | # enrolled per month | 8/month for 12 months |
| Randomization | Proportion enrolled who are randomized | 90% randomized to intervention and control arms^32^ |
| Fidelity | Observations of incentive payment delivery | Incentive payments that are due are delivered within 2 days of the visit by study staff |
| Adherence | Protocol adherence to the CO assessment schedule  Attendance to counseling sessions  Adherence to NRT among those prescribed NRT | 75% of those enrolled will provide a CO sample at 60%-70%, i.e., 14 -17 out of 25 possible assessment visits^32,33^  75% will attend at least 6 out of 12 counseling sessions during the treatment duration^33^  75% will adhere to using NRT for at least half of the treatment duration^33^ |
| Retention | Proportion of the sample retained as a result of retention procedures | 2 weeks (85%), 3-month (85%), 6-month (75%), and 1 year (75%) retention rates |
| Assessment | Proportion who completed all questionnaires | 75% completed all questionnaires |
| Acceptability | Questionnaire and qualitative interviews with staff and patients | 75% satisfied with the overall intervention |

Our threshold estimates for outcomes are based on findings from prior studies. For example, for the outcome of adherence to the CO assessment visits, a prior study showed that 78% of participants attended half of the assessment visits. Another study showed that participants provided a CO reading on average for 60% of the total possible assessment visits.^32,33^ We will assess the following process outcomes: number of counseling sessions attended (verified through the EHR), prescribed NRT (verified through the EHR), use of NRT and number of days used (determined by self-report at each assessment visit), total number of follow-up visits, number and amount of incentives received, and the incentive cost per quit attempt (i.e., the cost of incentives for a participant who achieves abstinence at each time point compared to a participant who does not). We will explore perceptions around CM payment amounts and perceived efficacy of the payment amounts in motivating long-term abstinence. We will conduct this evaluation using a combination of methods including notes/memos from staff during the study and in-depth interviews with clinic staff and patients at the TWUHC, RFPC, and PHP around satisfaction with the clinical trial. We will invite patient (n=15) and staff (n=8) participants to participate in a closing interview about the study.

## Concomitant Therapy

### Allowed Therapy

Participants can take bupropion or varenicline.

### Required Therapy

Participants will be required to participate in the on-site, standard cessation treatment that includes behavioral counseling and pharmacotherapy that includes nicotine replacement therapy provided by their medical team at the Tom Waddell Urban Health Clinic, Richard H. Fine People’s Clinic, and the Positive Health Program Clinic. It is possible that participants may recive counseling from different members of the medical team. We will be tracking this through follow-up questionnaires, as well as within the EHR through receipt of counseling (i.e., medical assistant, behavioral assistant and/or PCP counseling) and medications for cessation. HIPPA trained study staff will be trained to extract data on weekly receipt of counseling from the EHR. Two study staff will provide the incentive payments for demonstration of abstinence.

### Prohibited Therapy

Not applicable.

## Participant Discontinuation/Withdrawal from the Study

Participants are free to withdraw from participation in the study at any time upon request.

An investigator may discontinue a participant from the study for the following reasons:

- Unacceptable adverse event(s)
- Significant study intervention non-compliance, unless varying compliance is an aspect of the study objectives
- Lost-to-follow up; unable to contact participant (see Section 5.7 - Lost to Follow-Up)
- Any event or medical condition or situation occurs such that continued collection of follow-up study data would not be in the best interest of the participant or might require an additional treatment that would confound the interpretation of the study
- The participant meets an exclusion criterion (either newly developed or not previously recognized) that precludes further study participation

## Lost to Follow-up

A participant will be considered lost to follow-up if he or she fails to return for a scheduled visits and study staff are unable to contact the participant after at least 3 attempts. Before a participant is deemed lost to follow-up, the investigator or designee will make every effort to regain contact with the participant (where possible, 3 telephone calls and, if necessary, a letter to the participant’s last known mailing address or local equivalent methods). These contact attempts will be documented in the participant’s study file. Should the participant continue to be unreachable, he or she will be considered to have withdrawn from the study with a primary reason of lost to follow-up.

Participants will be asked to provide and update contact information at each visit. If a participant does not present for their scheduled visit, study staff will call the participants, or contact the participant’s contacts, or visit the places the participant reported that they spend time. Between the 6 month and 12 month assessment, participants will be asked and incentivized to check-in monthly by phone or in-person. We will hire two people who will assist with recruitment and retention efforts – patients seeking care at the TWUHC, RFPC, or PHP who have experienced homelessness and not participating in the trial – who will assist in contacting participants who are lost to follow-up.^52,53^ We have used these tracking procedures successfully in prior studies that have had a similar frequency of visits and length of follow-up.^61-63,65-69^ Based on prior work, we expect retention rates of 85% at 2 weeks, 85% at 3 months, and over 75% at 6-months and 1-year follow-up.^61-63,65-69^

# Study Procedures and Assessments

Participants will be asked to choose a quit date within 7 days of providing consent,^26,30,32,33,100^ and will meet study staff at the TWUHC, RFPC, or PHP on the day of the quit attempt (Table 3). Participants will meet daily for the first 7 days (week 1), twice weekly for the next 3 weeks (1 month), once weekly for the next 9 weeks (3 months), and monthly until the one-year follow-up (1 year). Assessment visit schedule is identical for both groups. At each of the scheduled assessment visits, participants will provide a CO sample and complete a short questionnaire on cigarette consumption, quit attempts since the last visit, the length of the last quit attempt, and use of medications and services. Questionnaires will be built on REDCap, and administered by study staff using an iPad. At enrollment, 2 weeks, 1 month, 3 months, 6 months, and 1-year follow-up, participants will be asked to complete structured questionnaires.

## Schedule of Activities

| **Assessments/Procedures** | **Screening** |  |  | **Study Intervention Period** |  | **End of Study Intervention** | **Follow-up** |
| --- | --- | --- | --- | --- | --- | --- | --- |
| **Study Visit / Day**  **(Window, # Days)** | **Visit -1/enrollment** | **Visits 2-8 / Week 1**  **Daily assessment** | **Visit 9-14 / Weeks 2- 4**  **Bi-weekly assessment** | **Visits 15-22/Weeks 5-13**  **Weekly assessment** | **Visits 23-25/Weeks 16-24**  **Monthly assessment** | **Visit 27 / Week 52**  **1 year follow-up** |  |
| Informed Consent^[[1]](#footnote-2)^ | **X** |  |  |  |  |  |  |
| Inclusion/Exclusion Criteria | **X** |  |  |  |  |  |  |
| Medical Record Review | **X** | **X** | **X** | **X** | **X** | **X** | **X** |
| Concomitant Medications | **X** | **X** | **X** | **X** | **X** | **X** |  |
| Adverse Events | **X** | **X** | **X** | **X** | **X** | **X** | **X** |
| Randomization/Assignment to Intervention | **X** |  |  |  |  |  |  |
| Administration of Study Intervention |  | **X** | **X** | **X** | **X** |  |  |
| Protocol-Specific Assessments/Procedures^2^ | **X** | **X** | **X** | **X** | **X** | **X^3^** | **X** |

## Study Procedures and Assessments

### Screening Period / Visit -1 (Day -0 to Day 1)

After an individual provides informed consent, the following activities will be performed during the Screening Period:

- Inclusion/exclusion criteria review
- Medical record review – smoking status and housing status
- Review of concomitant medications
- Adverse Events assessment
- Protocol-specific assessments/procedures
- Randomization/Assignment to Intervention

### Study Intervention Period

#### Visits 2-8 (Week 1)

- Review of concomitant medications
- Adverse Events assessment
- Administration of Study Intervention: Contingent incentives for smoking cessation
- Protocol-specific assessments/procedures: Daily expired carbon monoxide sample and a brief questionnaire

#### Visits 9-14 (Weeks 2-4)

- Review of concomitant medications
- Adverse Events assessment
- Administration of Study Intervention: Contingent incentives for smoking cessation
- Protocol-specific assessments/procedures: Expired carbon monoxide sample and a brief questionnaire bi-weekly; Questionnaires at 2 weeks and 1 months follow-up

#### Visits 15-23 (Week 5-13)

- Review of concomitant medications
- Adverse Events assessment
- Administration of Study Intervention: Contingent incentives for smoking cessation
- Protocol-specific assessments/procedures: Expired carbon monoxide sample and a brief questionnaire weekly; Questionnaires at 3 months follow-up;

#### Visits 24-26 (Weeks 16-24)

- Review of concomitant medications
- Adverse Events assessment
- Administration of Study Intervention: Contingent incentives for smoking cessation
- Protocol-specific assessments/procedures: Expired carbon monoxide sample and a brief questionnaire monthly between 3 and 6 months; Questionnaires at 6 months follow-up; At 6 months follow-up, if participants self-report abstinence, and have an expired carbon monoxide level < 5ppm, then we will collect a urine sample for anabasine/anatabine analysis.

### End of Study Intervention / Visit 27 (Week 52)

- Review of concomitant medications
- Adverse Events assessment
- Protocol-specific assessments/procedures: Expired carbon monoxide sample and an exit questionnaire at 12 months follow-up

### Follow-up

Participants will be followed daily for the first week, bi-weekly between 2 and 4 weeks, weekly for 4 weeks to 13 weeks, monthly from 16 weeks to 1 year after discontinuing the study intervention. The following procedures will be performed at each follow-up time point:

- Medical record review
- Adverse Events assessment

# Reporting and Documentation of Results

## Measures and Instruments

We will develop surveys in RedCap using our previously validated instruments in this population. Questionnaire measures will include:

| **Table 3: Questionnaire measures** |
| --- |
| **Sociodemographics**: Age, sex (male, female, transgender), race/ethnicity, education, monthly income, health insurance, health status, marital status |
| **Residential history**: Lifetime and past 6-months history of homelessness ^55^ |
| **Alternative tobacco and nicotine produce use:** Ever, past 30-day use of cigarettes and frequency of use of non-cigarette tobacco and nicotine products.^103^ |
| **Nicotine dependence:** Fagerstrom Test for Nicotine Dependence,^104^ Smoking urges,^105^ Confidence to quit.^103^ |
| **Cessation history**: Intention to quit, quit attempt (ever and in the past year), length of last quit attempt, use of cessation aids (medications, telephone quit line, health care provider advice ) ^97^ |
| **Substance use history**: Alcohol,^106^ and Substance Involvement Screening Test version 3.0 (WHO-ASSIST) during lifetime and in the past 6 months.^107^ |
| **Mental health disorders:**  Mini International Neuropsychiatric Interview v.6.0 ^108-111^ |
| **Chronic health conditions:** Self-reported chronic health conditions^112^ |

*Sociodemographic, residential history, and health status.* We will obtain information on age, sex (sex assigned at birth, current gender), race/ethnicity, education, monthly income (i.e. income from all sources), marital status, health insurance, and health status.^113^ For residential history, we will ask where participants had stayed the previous night (unsheltered, emergency shelter, transitional shelter, single room occupancy hotel, supportive housing). We will ask questions to assess whether they currently meet HEARTH criteria for homelessness,^96^ when their current episode of homelessness began, and when they were last stably housed.

*Nicotine dependence and tobacco cessation history.* We will assess Fagerstrom’s test for nicotine dependence,^104^  smoking urges,^105^ confidence to quit.^103^ We will assess cessation history including intention to quit, quit attempt (ever and in the past year), length of the last quit attempt, and use of cessation aids during the last quit attempt (e.g., medications, telephone quit line).

*Alternative tobacco and nicotine product use.* We will obtain information on ever use and use in the past 30 days of non-cigarette tobacco and nicotine products including electronic cigarettes, cigars, little cigars, smokeless tobacco, hookah/waterpipe, cannabis, and blunts.

*Chronic Diseases.* We will ask participants whether they have liver, renal, or cardiovascular disease, hypertension, diabetes, cancer, chronic obstructive pulmonary disease, or HIV.^114^

*Mental health.* We will conduct the Mini International Neuropsychiatric Interview v.6.0 (MINI 6.0),^108,115-117^ a brief structured interview that has been validated for assessing DSM-IV and ICD-10 psychiatric diagnoses (lifetime and current diagnosis for major depression, bipolar I and II, posttraumatic stress disorder, psychotic disorders, and generalized anxiety disorder).

*Alcohol and Substance Use and Use Disorders.* We will administer the Alcohol, Smoking and Substance Involvement Screening Test version 3.0 (WHO-ASSIST).^107,118-120^ In order to assess volume of alcohol consumed, we will administer the consumption questions from the Alcohol Use Disorders Identification Test (AUDIT-C).^106,121^

# Adverse Events and Serious Adverse Events

## Definition of Adverse Event

An adverse event (AE) is defined as any untoward medical occurrence associated with the use of an intervention in humans, whether or not considered intervention related.

## Definition of Serious Adverse Event

An AE that results in any of the following outcomes is defined as a Serious Adverse Event:

- Death,
- Life-threatening adverse experience*,
- Inpatient hospitalization or prolongation of existing hospitalization,
- Persistent or significant disability/incapacity,
- Congenital anomaly/birth defect, or cancer, or
- Any other experience that suggests a significant hazard, contraindication, side effect or precaution that may require medical or surgical intervention to prevent one of the outcomes listed above,
- Event that changes the risk/benefit ratio of the study.

*A life-threatening adverse experience is any AE that places the patient or subject, in the view of the investigator, at immediate risk of death from the reaction as it occurred, i.e., it does not include a reaction that, had it occurred in a more severe form, might have caused death.

## Classification of Adverse Events

### Severity

Adverse events are graded according to the Common Terminology Criteria for Adverse Events (CTCAE) as developed and revised by the Common Therapy Evaluation Program (CTEP) of the National Cancer Institute.

### Attribution

Adverse events are further given an assignment of attribution or relationship to study intervention or procedure. Attribution categories are:

- **Definite** – The adverse event is clearly related to the study intervention or procedure.
- **Probable** – The adverse event is likely related to the study intervention or procedure.
- **Possible** – The adverse event may be related to the study intervention or procedure.
- **Unrelated** – the adverse event is clearly not related to the study intervention or procedure.

### Expectedness

An adverse event is considered unexpected if it is not listed in the investigator brochure or package insert(s), or is not listed at the specificity or severity that has been observed, or, if an investigator brochure is not required or available, the event is not consistent with the risk information described in the general investigational plan or elsewhere in the current application.

## Adverse Events Monitoring

This study is a minimal risk level study that does not require monitoring by the HDFCCC Data and Safety Monitoring Committee (DSMC) as per the National Cancer Institute-approved Data and Safety Monitoring Plan. Ultimately, the PI is responsible for the safety and conduct of this study.

## Follow up of Adverse Events

All participants who experience adverse events will be followed with appropriate medical management until resolved or stabilized, as determined by the investigator.

## Documenting and Reporting of Adverse Events

Adverse Events will be documented in the study Case Report Forms (CRFs) and reported to the IRB, HDFCCC DSMC, and collaborators in accordance with all applicable institutional and regulatory requirements.

# Statistical Considerations

## Sample Size Considerations

### Sample Size and Power Estimate

Because pilot studies are too small to definitively test hypotheses or estimate precise

effect sizes, we do not provide power analyses. As the primary aim of this pilot study is to assess feasibility and acceptability of the research protocol for a future clinical trial, the sample size of *N*=90 (45 in each condition) was set primarily for practical reasons and not driven by hypothesis testing or estimating precise effect size estimates. Effect sizes used to inform power analysis for a future full-scale RCT will be based on clinically important differences in primary abstinence outcomes.

### Randomization and Blinding

See Section 4.6

### Stratification Factors

Not applicable.

### Accrual Estimates

We will recruit all participants by 1 year.

## Interim Analyses and Stopping Rules

Not applicable

## Statistical Analysis Plans

1. ***Statistical analyses****.* Because pilot studies are too small to definitively test hypotheses or estimate precise effect sizes, we do not propose any inferential statistics. Primary analyses will include descriptive statistics of the feasibility and acceptability indicators, comparing each statistic (e.g., % retained) to its tabled threshold (above, see Section 5.2). Above-threshold findings will suggest a reasonable level of feasibility and acceptability for the corresponding aspects of study procedures. Any sub-threshold finding would suggest that remedial modifications to study procedures and/or design would be required prior to moving forward with a full-scale RCT. We also will examine descriptive statistics of the primary and secondary clinical outcomes and include exploratory analyses of relevant biological variables such as age, sex, and race/ethnicity. To the extent possible, descriptive anaylses will consider whether feasibility and acceptability outcomes were consistent across service providers and service settings.

.

### Analysis Populations

Not applicable

### Primary Analysis (or Analysis of Primary Endpoints)

We will conduct exploratory analyses to assess feasibility and acceptability of collecting data on primary, secondary and other outcomes.

### Secondary Analysis (or Analysis of Secondary Endpoints)

### Exploratory/Correlative Analysis/Assessments

# Study Management

## Pre-study Documentation

Before initiating this trial, the PI will have written and dated approval from the Institutional Review Board for the protocol, written informed consent form, subject recruitment materials, and any other written information to be provided to participants before any protocol related procedures are performed on any participants.

The PI must comply with GCP/ICH guidelines and all applicable regulatory requirements.

## Institutional Review Board Approval

The protocol, the proposed informed consent form, and all forms of participant-facing materials related to the study (e.g., advertisements used to recruit participants) will be reviewed and approved by the IRB. The initial protocol and all protocol amendments must be approved by the IRB prior to implementation.

## Informed Consent

All participants must be provided a consent form describing the study with sufficient information for each participant to make an informed decision regarding their participation. Participants must sign the IRB-approved informed consent form prior to participation in any study specific procedure. The participant must receive a copy of the signed and dated consent document. The original signed copy of the consent document must be retained in the medical record or research file.

## Changes in the Protocol

Once the protocol has been approved by the IRB, any changes to the protocol must be documented in the form of an amendment. The amendment must be signed by the PI and approved by the IRB prior to implementation.

If it becomes necessary to alter the protocol to eliminate an immediate hazard to participants, an amendment may be implemented prior to IRB approval. In this circumstance, however, the PI must then notify the IRB according to institutional requirements.

*For multicenter studies add:* The Study Chair and the UCSF study team will be responsible for updating any participating sites.

## Case Report Forms (CRFs)

The PI will prepare and maintain adequate and accurate participant case histories with observations and data pertinent to the study. Study specific Case Report Forms (CRFs) will document safety and treatment outcomes for safety monitoring and data analysis. All study data will be entered into OnCore^®^ via standardized CRFs in accordance with the CTMS study calendar, using single data entry with a secure access account. Study personnel will complete the CRFs; the PI will review and approve the completed CRFs.

The information collected on CRFs shall be identical to that appearing in original source documents. Source documents will be found in the participant’s medical records maintained by study personnel. All source documentation should be kept in separate research files for each participant.

In accordance with federal regulations, the PI is responsible for the accuracy and authenticity of all clinical and laboratory data entered onto CRFs. The PI will approve all completed CRFs to attest that the information contained on the CRFs is true and accurate.

The PI will be responsible for ensuring the accurate capture of study data. At study completion, when the CRFs have been declared to be complete and accurate, the database will be locked. Any changes to the data entered into the CRFs after that time can only be made by joint written agreement among the and the trial statistician.

All source documentation and CTMS data will be available for review/monitoring by the UCSF DSMC and regulatory agencies.

## Record Retention

The PI is required to prepare and maintain adequate and accurate case histories that record all observations and other data pertinent to the investigation on each study participant. Study documentation includes all CRFs, data correction forms or queries, source documents, Sponsor-Investigator correspondence, monitoring logs/letters, and regulatory documents (e.g., protocol and amendments, IRB correspondence and approval, signed participant consent forms). Source documents include all recordings of observations or notations of clinical activities and all reports and records necessary for the evaluation and reconstruction of the clinical research study. The PI shall retain records for a period of 2 years following the conclusion of the study.

## Publications

The preparation and submittal for publication of manuscripts containing the study results shall be in accordance with a process determined by mutual written agreement among the study’s investigators.

# References

1. Baggett TP, Tobey ML, Rigotti NA. Tobacco use among homeless people--addressing the neglected addiction. N Engl J Med;369:201-4.

2. Creamer MR, Wang TW, Babb S, et al. Tobacco Product Use and Cessation Indicators Among Adults - United States, 2018. MMWR Morb Mortal Wkly Rep 2019;68:1013-9.

3. Baggett TP, Chang Y, Singer DE, et al. Tobacco-, alcohol-, and drug-attributable deaths and their contribution to mortality disparities in a cohort of homeless adults in Boston. Am J Public Health 2015;105:1189-97.

4. Hwang SW. Homelessness and health. CMAJ 2001;164:229-33.

5. Lee TC, Hanlon JG, Ben-David J, et al. Risk factors for cardiovascular disease in homeless adults. Circulation 2005;111:2629-35.

6. Hwang SW, Orav EJ, O'Connell JJ, Lebow JM, Brennan TA. Causes of death in homeless adults in Boston. Ann Intern Med 1997;126:625-8.

7. Baggett TP, Hwang SW, O'Connell JJ, et al. Mortality among homeless adults in Boston: shifts in causes of death over a 15-year period. JAMA Intern Med 2013;173:189-95.

8. Hwang SW, Wilkins R, Tjepkema M, O'Campo PJ, Dunn JR. Mortality among residents of shelters, rooming houses, and hotels in Canada: 11 year follow-up study. BMJ 2009;339:b4036.

9. Connor SE, Cook RL, Herbert MI, Neal SM, Williams JT. Smoking cessation in a homeless population: there is a will, but is there a way? J Gen Intern Med 2002;17:369-72.

10. Vijayaraghavan M, Pierce JP. Interest in Smoking Cessation Related to a Smoke-Free Policy Among Homeless Adults. Journal of community health 2015.

11. Vijayaraghavan M, Penko J, Vittinghoff E, Bangsberg DR, Miaskowski C, Kushel MB. Smoking Behaviors in a Community-Based Cohort of HIV-Infected Indigent Adults. AIDS Behav.

12. Baggett TP, Lebrun-Harris LA, Rigotti NA. Homelessness, cigarette smoking and desire to quit: results from a US national study. Addiction.

13. Shelley D, Cantrell J, Wong S, Warn D. Smoking cessation among sheltered homeless: a pilot. Am J Health Behav;34:544-52.

14. Okuyemi KS, Thomas JL, Hall S, et al. Smoking cessation in homeless populations: a pilot clinical trial. Nicotine Tob Res 2006;8:689-99.

15. Spector A, Alpert H, Karam-Hage M. Smoking cessation delivered by medical students is helpful to homeless population. Acad Psychiatry 2007;31:402-5.

16. Segan CJ, Maddox S, Borland R. Homeless Clients Benefit From Smoking Cessation Treatment Delivered by a Homeless Persons' Program. Nicotine Tob Res 2015;17:996-1001.

17. Okuyemi KS, Goldade K, Whembolua GL, et al. Motivational interviewing to enhance nicotine patch treatment for smoking cessation among homeless smokers: a randomized controlled trial. Addiction 2013;108:1136-44.

18. Gilpin EA, Pierce JP, Farkas AJ. Duration of smoking abstinence and success in quitting. J Natl Cancer Inst 1997;89:572-6.

19. Milat AJ, King L, Bauman AE, Redman S. The concept of scalability: increasing the scale and potential adoption of health promotion interventions into policy and practice. Health Promot Int 2013;28:285-98.

20. Barker PM, Reid A, Schall MW. A framework for scaling up health interventions: lessons from large-scale improvement initiatives in Africa. Implement Sci 2016;11:12.

21. Higgins ST, Silverman K, Sigmon SC, Naito NA. Incentives and health: an introduction. Prev Med 2012;55 Suppl:S2-6.

22. Stitzer ML, Rand CS, Bigelow GE, Mead AM. Contingent payment procedures for smoking reduction and cessation. J Appl Behav Anal 1986;19:197-202.

23. Stitzer ML, Bigelow GE. Contingent reinforcement for reduced carbon monoxide levels in cigarette smokers. Addict Behav 1982;7:403-12.

24. Cahill K, Hartmann-Boyce J, Perera R. Incentives for smoking cessation. Cochrane Database Syst Rev 2015;5:CD004307.

25. Tidey JW, O'Neill SC, Higgins ST. Contingent monetary reinforcement of smoking reductions, with and without transdermal nicotine, in outpatients with schizophrenia. Experimental and clinical psychopharmacology 2002;10:241-7.

26. Dunn KE, Sigmon SC, Reimann EF, Badger GJ, Heil SH, Higgins ST. A contingency-management intervention to promote initial smoking cessation among opioid-maintained patients. Experimental and clinical psychopharmacology 2010;18:37-50.

27. Etter JF. Financial incentives for smoking cessation in low-income smokers: study protocol for a randomized controlled trial. Trials 2012;13:88.

28. Kendzor DE, Businelle MS, Poonawalla IB, et al. Financial incentives for abstinence among socioeconomically disadvantaged individuals in smoking cessation treatment. Am J Public Health 2015;105:1198-205.

29. Stitzer ML, Bigelow GE, Liebson IA, Hawthorne JW. Contingent reinforcement for benzodiazepine-free urines: evaluation of a drug abuse treatment intervention. J Appl Behav Anal 1982;15:493-503.

30. Carpenter VL, Hertzberg JS, Kirby AC, et al. Multicomponent smoking cessation treatment including mobile contingency management in homeless veterans. J Clin Psychiatry 2015;76:959-64.

31. Businelle MS, Kendzor DE, Kesh A, et al. Small financial incentives increase smoking cessation in homeless smokers: a pilot study. Addict Behav 2014;39:717-20.

32. Baggett TP, Chang Y, Yaqubi A, McGlave C, Higgins ST, Rigotti NA. Financial Incentives for Smoking Abstinence in Homeless Smokers: A Pilot Randomized Controlled Trial. Nicotine Tob Res 2018;20:1442-50.

33. Rash CJ, Petry NM, Alessi SM. A randomized trial of contingency management for smoking cessation in the homeless. Psychol Addict Behav 2018;32:141-8.

34. Sigmon SC, Miller ME, Meyer AC, et al. Financial incentives to promote extended smoking abstinence in opioid-maintained patients: a randomized trial. Addiction 2016;111:903-12.

35. Davis DR, Kurti AN, Skelly JM, Redner R, White TJ, Higgins ST. A review of the literature on contingency management in the treatment of substance use disorders, 2009-2014. Prev Med 2016;92:36-46.

36. Secades-Villa R, Garcia-Rodriguez O, Garcia-Fernandez G, Sanchez-Hervas E, Fernandez-Hermida JR, Higgins ST. Community reinforcement approach plus vouchers among cocaine-dependent outpatients: twelve-month outcomes. Psychol Addict Behav 2011;25:174-9.

37. Stitzer M, Calsyn D, Matheson T, Sorensen J, Gooden L, Metsch L. Development of a Multi-Target Contingency Management Intervention for HIV Positive Substance Users. J Subst Abuse Treat 2017;72:66-71.

38. Carpenedo CM, Kirby KC, Dugosh KL, Rosenwasser BJ, Thompson DL. Extended voucher-based reinforcement therapy for long-term drug abstinence. Am J Health Behav 2010;34:776-87.

39. Kirby KC, Carpenedo CM, Dugosh KL, et al. Randomized clinical trial examining duration of voucher-based reinforcement therapy for cocaine abstinence. Drug Alcohol Depend 2013;132:639-45.

40. Research AS. San Francisco 2017 Homeless Count & Survey. <http://hshsfgovorg/wp-content/uploads/2017/06/2017-SF-Point-in-Time-Count-General-FINAL-62117pdf> 2017.

41. Vijayaraghavan M, Hurst S, Pierce JP. Implementing Tobacco Control Programs in Homeless Shelters: A Mixed-Methods Study. Health Promot Pract 2015;17:501-11.

42. Baggett TP, Rigotti NA. Cigarette smoking and advice to quit in a national sample of homeless adults. Am J Prev Med 2010;39:164-72.

43. Gelberg L, Linn LS, Usatine RP, Smith MH. Health, homelessness, and poverty. A study of clinic users. Arch Intern Med 1990;150:2325-30.

44. Fazel S, Geddes JR, Kushel M. The health of homeless people in high-income countries: descriptive epidemiology, health consequences, and clinical and policy recommendations. Lancet 2014;384:1529-40.

45. Okuyemi KS, Caldwell AR, Thomas JL, et al. Homelessness and smoking cessation: insights from focus groups. Nicotine Tob Res 2006;8:287-96.

46. Porter J, Houston L, Anderson RH, Maryman K. Addressing tobacco use in homeless populations: recommendations of an expert panel. Health Promot Pract 2011;12:144S-51S.

47. Butler J, Okuyemi KS, Jean S, Nazir N, Ahluwalia JS, Resnicow K. Smoking characteristics of a homeless population. Subst Abus 2002;23:223-31.

48. Fiore MC JC, Baker T, et al. Treating Tobacco Use and Dependence: 2008 Update. Clinical Practice Guideline. . Rockville, MD: US Dept of Health and Human Services, Public Health Service; 2008 2008.

49. Troxel AB, Volpp KG. Effectiveness of financial incentives for longer-term smoking cessation: evidence of absence or absence of evidence? Am J Health Promot 2012;26:204-7.

50. Sigmon SC, Patrick ME. The use of financial incentives in promoting smoking cessation. Prev Med 2012;55 Suppl:S24-32.

51. Baggett T, Chang, Y., Awesta Y., McGlave C., Higgins S.T., Rigotti N.A. Financial incentives for smoking cessation: A pilot randomized controlled trial. Nicotine and Tobacco Research 2017;Advance Access Publication, Aug. 18 2017.

52. Olamide Ojo-Fati JA, Ig-Izevbekhia J, Thomas JL, Everson-Rose SA, Pratt R, Raymond N, Cooney NL, Luo X, Okuyemi KS. Practical issues regarding implementing a randomized clinical trial in a homeless population: strategies and lessons learned. Trials 2017;18.

53. Goldade K WG, Thomas J, et al. Designing a smoking cessation intervention for the unique needs of homeless persons: a community-based randomized clinical trial. Clin Trials 2011;8:744-54.

54. Vijayaraghavan M, Hurst S, Pierce JP. A Qualitative Examination of Smoke-Free Policies and Electronic Cigarettes Among Sheltered Homeless Adults. Am J Health Promot 2017;31:243-50.

55. Vijayaraghavan M, Tieu L, Ponath C, Guzman D, Kushel M. Tobacco Cessation Behaviors Among Older Homeless Adults: Results From the HOPE HOME Study. Nicotine Tob Res 2016.

56. Neisler J, Reitzel LR, Garey L, et al. Concurrent nicotine and tobacco product use among homeless smokers and associations with cigarette dependence and other factors related to quitting. Drug Alcohol Depend 2018;185:133-40.

57. Gubner NR, Williams DD, Chen E, et al. Recent cessation attempts and receipt of cessation services among a diverse primary care population - A mixed methods study. Prev Med Rep 2019;15:100907.

58. Vijayaraghavan M, Dove MS, Stewart SL, et al. Racial/Ethnic Differences in the Response to Incentives for Quitline Engagement. Am J Prev Med 2018;55:S186-S95.

59. Tong EK, Stewart SL, Schillinger D, et al. The Medi-Cal Incentives to Quit Smoking Project: Impact of Statewide Outreach Through Health Channels. Am J Prev Med 2018;55:S159-S69.

60. Centers for Medicare and Medicaid Services. Medicaid Incentives for the Prevention of Chronic Diseases Model. 2016; <https://innovation.cms.gov/initiatives/mipcd/>. Accessed September.

61. Kushel MB, Colfax G, Ragland K, Heineman A, Palacio H, Bangsberg DR. Case management is associated with improved antiretroviral adherence and CD4+ cell counts in homeless and marginally housed individuals with HIV infection. Clin Infect Dis 2006;43:234-42.

62. Kushel MB, Perry S, Bangsberg D, Clark R, Moss AR. Emergency department use among the homeless and marginally housed: results from a community-based study. Am J Public Health 2002;92:778-84.

63. Weiser SD, Bangsberg DR, Kegeles S, Ragland K, Kushel MB, Frongillo EA. Food insecurity among homeless and marginally housed individuals living with HIV/AIDS in San Francisco. AIDS Behav 2009;13:841-8.

64. Weiser SD, Bangsberg DR, Kegeles S, Ragland K, Kushel MB, Frongillo EA. Food Insecurity Among Homeless and Marginally Housed Individuals Living with HIV/AIDS in San Francisco. AIDS Behav 2009.

65. Vijayaraghavan M, Penko J, Guzman D, Miaskowski C, Kushel MB. Primary Care Providers' Judgments of Opioid Analgesic Misuse in a Community-Based Cohort of HIV-Infected Indigent Adults. J Gen Intern Med 2010.

66. Thom DH, Wong ST, Guzman D, et al. Physician trust in the patient: development and validation of a new measure. Ann Fam Med 2011;9:148-54.

67. Hansen L, Penko J, Guzman D, Bangsberg D, Miaskowski C, Kushel MB. Aberrant Behaviors with Prescription Opioids and Problem Drug Use History in a Community-Based Cohort of HIV Infected Individuals. J Pain and Symptom Management 2011.

68. Miaskowski C, Penko J, Guzman D, Mattson J, Bangsberg DR, Kushel MB. Occurrence and characteristics of chronic pain in a community-based cohort of indigent adults living with HIV infection. J Pain 2011.

69. Vijayaraghavan M, Penko J, Bangsberg DR, Miaskowski C, Kushel MB. Opioid Analgesic Misuse in a Community-Based Cohort of HIV-Infected Indigent Adults. JAMA Intern Med;173:235-7.

70. Vijayaraghavan M, Yuan P, Gregorich S, et al. Disparities in receipt of 5As for smoking cessation in diverse primary care and HIV clinics. Prev Med Rep 2017;6:80-7.

71. Prochaska JJ, Gill P, Hall SM. Treatment of tobacco use in an inpatient psychiatric setting. Psychiatr Serv 2004;55:1265-70.

72. Hall SM, Humfleet GL, Munoz RF, Reus VI, Robbins JA, Prochaska JJ. Extended treatment of older cigarette smokers. Addiction 2009;104:1043-52.

73. Prochaska JJ, Delucchi K, Hall SM. A meta-analysis of smoking cessation interventions with individuals in substance abuse treatment or recovery. J Consult Clin Psychol 2004;72:1144-56.

74. Carmody TP, Delucchi K, Duncan CL, et al. Intensive intervention for alcohol-dependent smokers in early recovery: A randomized trial. Drug Alcohol Depend.

75. Kaplan CP, Livaudais-Toman J, Tice JA, et al. A randomized, controlled trial to increase discussion of breast cancer in primary care. Cancer Epidemiol Biomarkers Prev 2014;23:1245-53.

76. Wang EA, Hong CS, Shavit S, Sanders R, Kessell E, Kushel MB. Engaging individuals recently released from prison into primary care: a randomized trial. Am J Public Health 2012;102:e22-9.

77. Dallery J, Raiff BR, Kim SJ, Marsch LA, Stitzer M, Grabinski MJ. Nationwide access to an internet-based contingency management intervention to promote smoking cessation: a randomized controlled trial. Addiction 2017;112:875-83.

78. Vijayaraghavan M, Hurst S, Pierce JP. Implementing Tobacco Control Programs in Homeless Shelters: A Mixed-Methods Study. Health Promot Pract 2015.

79. Napoles AM, Appelle N, Kalkhoran S, Vijayaraghavan M, Alvarado N, Satterfield J. Perceptions of clinicians and staff about the use of digital technology in primary care: qualitative interviews prior to implementation of a computer-facilitated 5As intervention. BMC Med Inform Decis Mak 2016;16:44.

80. Kim-Hwang JE, Chen AH, Bell DS, Guzman D, Yee HF, Jr., Kushel MB. Evaluating electronic referrals for specialty care at a public hospital. J Gen Intern Med;25:1123-8.

81. Wang EA, Hong CS, Samuels L, Shavit S, Sanders R, Kushel M. Transitions clinic: creating a community-based model of health care for recently released California prisoners. Public Health Rep;125:171-7.

82. Chan B, Goldman LE, Sarkar U, et al. The Effect of a Care Transition Intervention on the Patient Experience of Older Multi-Lingual Adults in the Safety Net: Results of a Randomized Controlled Trial. J Gen Intern Med 2015;30:1788-94.

83. Vijayaraghavan M, Guydish J, Pierce JP. Building Tobacco Cessation Capacity in Homeless Shelters: A Pilot Study. Journal of community health 2016;41:998-1005.

84. Hagedorn HJ, Stetler CB, Bangerter A, Noorbaloochi S, Stitzer ML, Kivlahan D. An implementation-focused process evaluation of an incentive intervention effectiveness trial in substance use disorders clinics at two Veterans Health Administration medical centers. Addict Sci Clin Pract 2014;9:12.

85. Alizaga NM, Nguyen T, Petersen AB, Elser H, Vijayaraghavan M. Developing Tobacco Control Interventions in Permanent Supportive Housing for Formerly Homeless Adults. Health Promot Pract 2019:1524839919839358.

86. Petersen AB, Elser H, Nguyen T, Alizaga NM, Vijayaraghavan M. Smoke-Free or Not: Attitudes Toward Indoor Smoke-Free Policies Among Permanent Supportive Housing Residents. Am J Health Promot 2020;34:32-41.

87. Ajzen I. The Theory of Planned Behavior. Organization Behavior and Human Decision Processes 1991;50:179-211.

88. Ford JG FJ, Howerton MW, Lai GY, Gary TL, Bolen S, Gibbons MC, Tilburt J, Baffi C, Tanpitukpongse TP, Wilson RF, Powe NR, Bass EB. Barriers to recruiting underrepresented populations to cancer clinical trials: a systematic review. Cancer 2008;112:228-42.

89. Ford JG HM, Bolen S, Gary TL, Lai GY, Tilburt J, Gibbons MC, Baffi C, Wilson RF, Feuerstein CJ, Tanpitukpongse P, Powe NR, Bass EB. . Knowledge and Access to Information on Recruitment of Underrepresented Populations to Cancer Clinical Trials. Summary, Evidence Report/Technology Assessment No. 122. Prepared by the Johns Hopkins University Evidence-based Practice Center, under Contract No 290-02-0018) AHRQ Publication No 05-E019-1 Rockville, MD: Agency for Healthcare Research and Quality 2005.

90. Hsieh HF, Shannon SE. Three approaches to qualitative content analysis. Qual Health Res 2005;15:1277-88.

91. Pilot Studies: Common Uses and Misuses. 2017. (Accessed February 14, 2018, at <https://nccih.nih.gov/grants/whatnccihfunds/pilot_studies?nav=govd>.)

92. Kraemer HC, Mintz J, Noda A, Tinklenberg J, Yesavage JA. Caution regarding the use of pilot studies to guide power calculations for study proposals. Archives of general psychiatry 2006;63:484-9.

93. Leon AC, Davis LL, Kraemer HC. The role and interpretation of pilot studies in clinical research. Journal of psychiatric research 2011;45:626-9.

94. Kistin C, Silverstein M. Pilot Studies: A Critical but Potentially Misused Component of Interventional Research. JAMA 2015;314:1561-2.

95. <http://www.lung.org/stop-smoking/join-freedom-from-smoking/about-freedom-from-smoking.html>. Freedom from Smoking American Lung Association.

96. Act TM-VHA. General Definition of Homeless Individuals. <https://wwwonecpdinfo/resources/documents/homelessassistanceactamendedbyhearthpdf> 2009.

97. Survey CT. California Tobacco Surveys. <http://librariesucsdedu/locations/sshl/data-gov-info-gis/ssds/guides/tobacco-surveyshtml>.

98. Verification SSoB. Biochemical Verification of tobacco use and cessation. Nicotine Tob Res 2001;4:149-59.

99. Sudore RL, Landefeld CS, Williams BA, Barnes DE, Lindquist K, Schillinger D. Use of a modified informed consent process among vulnerable patients: a descriptive study. J Gen Intern Med 2006;21:867-73.

100. Higgins ST, Heil SH, Solomon LJ, et al. A pilot study on voucher-based incentives to promote abstinence from cigarette smoking during pregnancy and postpartum. Nicotine Tob Res 2004;6:1015-20.

101. Jacob P HD, Severson H, Hall S, Yu L, Benowitz NL. Anabasine and anatabine as biomarkers for tobacco use during nicotine replacement therapy. Cancer Epidemiol Biomarkers Prev 2002;11:1668-73.

102. Core UTB. <http://cancer.ucsf.edu/research/cores/tobacco-biomarkers/>. 2017.

103. Al-Delaimy W, Messer K, Pierce J, Trinidad D, White M. Technical Report on Analytic Methods and Approaches Used in the 2005 California Tobacco Survey Analysis. La Jolla, CA: University of California, San Diego; 2007.

104. Heatherton TF, Kozlowski LT, Frecker RC, Fagerstrom KO. The Fagerstrom Test for Nicotine Dependence: a revision of the Fagerstrom Tolerance Questionnaire. Br J Addict 1991;86:1119-27.

105. Tiffany ST, Drobes DJ. The development and initial validation of a questionnaire on smoking urges. Br J Addict 1991;86:1467-76.

106. Bush K, Kivlahan DR, McDonell MB, Fihn SD, Bradley KA. The AUDIT alcohol consumption questions (AUDIT-C): an effective brief screening test for problem drinking. Ambulatory Care Quality Improvement Project (ACQUIP). Alcohol Use Disorders Identification Test. Arch Intern Med 1998;158:1789-95.

107. WHO Assist Working Group. The Alcohol, Smoking and Substance Involvement Screening Test (ASSIST): development, reliability and feasibility. Addiction 2002;97:1183-94.

108. Sheehan DV, Lecrubier Y, Sheehan KH, et al. The Mini-International Neuropsychiatric Interview (M.I.N.I.): the development and validation of a structured diagnostic psychiatric interview for DSM-IV and ICD-10. J Clin Psychiatry 1998;59 Suppl 20:22-33;quiz 4-57.

109. Amorim P, Lecrubier Y, Weiller E, Hergueta T, Sheehan D. DSM-III-R psychotic disorders: Procedural validity of the Mini International Neuropsychiatric Interview (MINI): Concordance and causes for discordance with the CIDI. European Psychiatry 1998;13:26-34.

110. Lecrubier Y, Sheehan DV, Weiller E, et al. The Mini International Neuropsychiatric Interview (MINI): A short diagnostic structured interview: Reliability and validity according to the CIDI. European Psychiatry 1997;12:224-31.

111. Sheehan DV, Lecrubier Y, Sheehan KH, et al. The validity of the Mini International Neuropsychiatric Interview (MINI) according to the SCID-P and its reliability. European Psychiatry 1997;12:232-41.

112. National Health Interview Survey Questionnaire 2010. at <ftp://ftp.cdc.gov/pub/Health_Statistics/NCHS/Survey_Questionnaires/NHIS/2010/English/qadult.pdf>.)

113. Larson CO. Use of the SF-12 instrument for measuring the health of homeless persons. Health Serv Res 2002;37:733-50.

114. National Health Interview Survey. <https://www.cdc.gov/nchs/nhis/data-questionnaires-documentation.htm>: Centers for Disease Control and Prevention; 2017.

115. Lecrubier Y, Sheehan DV, Weiller E, et al. The Mini International Neuropsychiatric Interview (MINI). A short diagnostic structured interview: reliability and validity according to the CIDI. European Psychiatry 1997;12:224-31.

116. Sheehan DV, Lecrubier Y, Harnett Sheehan K, et al. The validity of the Mini International Neuropsychiatric Interview (MINI) according to the SCID-P and its reliability. European Psychiatry 1997;12:232-41.

117. Amorim P, Lecrubier Y, Weiller E, Hergueta T, Sheehan D. DSM-IH-R Psychotic Disorders: procedural validity of the Mini International Neuropsychiatric Interview (MINI). Concordance and causes for discordance with the CIDI. Eur Psychiatry 1998;13:26-34.

118. Humeniuk R, Ali R, Babor TF, et al. Validation of the Alcohol, Smoking And Substance Involvement Screening Test (ASSIST). Addiction 2008;103:1039-47.

119. Newcombe DA, Humeniuk RE, Ali R. Validation of the World Health Organization Alcohol, Smoking and Substance Involvement Screening Test (ASSIST): report of results from the Australian site. Drug Alcohol Rev 2005;24:217-26.

120. The Alcohol, Smoking and Substance Involvement Screening Test (ASSIST): development, reliability and feasibility. Addiction 2002;97:1183-94.

121. Aalto M, Alho H, Halme JT, Seppa K. The Alcohol Use Disorders Identification Test (AUDIT) and its derivatives in screening for heavy drinking among the elderly. International journal of geriatric psychiatry 2011;26:881-5.

122. Piper ME, Bullen C, Krishnan-Sarin S, et al. Defining and measuring abstinence in clinical trials of smoking cessation interventions: An updated review. Nicotine Tob Res 2019.

123. Shenkman E, Muller K, Vogel B, et al. The wellness incentives and navigation project: design and methods. BMC Health Serv Res 2015;15:579.

124. Centers for Medicare & Medicaid Services. Patient protection and Affordable Care Act section 4108 MifpocdM, initial announcement. 2011. (CMS Publication No. CMS-1B1-11-001). Retrieved from <http://innovation.cms.gov/Files/fact-> sheet/MIPCD-Funding-Opportunity-Announcement.pdf.

125. Hand DJ, Heil SH, Sigmon SC, Higgins ST. Improving medicaid health incentives programs: lessons from substance abuse treatment research. Prev Med 2014;63:87-9.

Appendix 1: Data and Safety Monitoring Plan

Procedures for implementing a Data Safety and Monitoring Plan (DSMP) at the University of California San Francisco have been developed on a campus-wide basis in conjunction with the Institutional Review Board (Committee on Human Research), the Office of the Vice-Chancellor for Research and the Office of Clinical Research in the School of Medicine. The Data and Safety Monitoring Plan for the proposed project incorporates the policies on human subject data and safety monitoring specified by the UCSF Committee on Human Research (i.e. UCSF Institutional Review Board).

# 1.  Risk Assessment - Minimal Risk

This study represents a minimal risk intervention. In this study, we will recruit 90 participants who are current smokers who have an intention to quit smoking within 6 months and who are homeless into a pilot randomized controlled trial of a contingency management (CM) smoking cessation intervention (see Research Strategy section). In this trial we will assess feasibility and acceptability of the study protocol, including CM for smoking cessation, escalating incentives, and study procedures for recruitment and retention. This study will provide preliminary data and inform development for the subsequent full-scale RCT to test the efficacy of the CM intervention for long-term absitnence. Participants will receive escalating incentive payments for demonstrating biochemically-verified smoking abstinence. The proposed study will be conducted by study staff in a private room at the Tom Waddell Urban Health Clinic (Aim 2). Staff will be well-trained in the program components. All data will be stored in a password protected database, on a secure UCSF server. The inclusion criteria do not allow individuals at very high risk for adverse events to be enrolled (e.g., recent myocardial infarction, or pregnancy). Participants will be required to attend usual smoking cessation care at the Tom Waddell Urban Health Clinic, which involves weekly one-on-one counseling conducted by behavioral counselors and receipt of nicotine replacement therapy (NRT) (the primary cessation medication in the clinical trial) dispensed on-site by a pharmacist and covered by participants’ health insurance. The study will provide a 12-week supply of NRT to participants who are unable to get covered medications due to insurance-related barriers. Pharmacists will determine dosage of NRT based on articipants’ current consumption level and nicotine dependence, and will assess whether the dose is meeting their needs. Primary care providers (PCPs) will verify and document in the EHR that there are no contraindications to receiving NRT such as having had a myocardial infarction in the 2 weeks prior to enrollment or pregnancy. HIPAA-trained study staff will access the EHR weekly to verify participants’ attendance of counseling and receipt of NRT. We anticipate that some participants may be co-prescribed varenicline or bupropion with NRT; we will document receipt of non-NRT medications by self-report at each visit and verify this information through the EHR. To decrease the likelihood of an adverse event during the study, study staff will provide information on the medications, directions of  use, indications of use, side effects of medications at the enrollment visit. At each assessment visit, study staff will assess adherence to medication and side effects of medications. Participants will also be asked to call study staff in between visits if new symptoms were to arise.

# 2.  Description of Adverse Event Grading and Anticipated Adverse Events

In this study, we do not anticipate moderate or severe adverse events (AE).  AE will be scored as follows:

1. = No adverse event or within normal limits
2. = Mild AE – not requiring follow-up
3. = Moderate AE – resolved with follow-up
4. = Severe AE – resulted in inability to carry on normal activities and required professional medical attention, requiring hospitalization
5. = Life-threatening or disabling AE – results in an immediate risk of death and/or results in persistent or significant disability
6. = Fatal AE

We anticipate at most mild AE, including potential adverse effects of nicotine withdrawal or hunger among those reducing consumption or quitting cigarette smoking. Risks of participating in one-on-one counseling sessions and assessment interviews include fatigue from answering questions, worry about loss of confidentiality/privacy, and perception of coercion to participate. The Principal Investigator (PI), Maya Vijayaraghavan, in consultation with the research team, is responsible for evaluating each AE and for determining whether the AE affects the risk/benefit ratio of the study and whether modifications to the protocol and consent form are required.

Participants will attend abstinence assessment visits according to the following schedule: daily visits for the 1^st^week, twice weekly for the next 3 weeks (1 month), once weekly for the next 8 weeks (3 months), and monthly until the one-year follow-up (1 year). In addition, participants will be required to attend cessation services at the TWUHC that include behavioral counseling and pharmacotherapy. At each visit, we will ask participants whether they had experienced any adverse effects since the last visit. Participants will also be encouraged to call the study team in between visits if they were to occur.  Adverse events will be graded according to their significance for severe consequences, such as injury or death.

1. Examples of severe adverse events (life-threatening or disabling and requiring medical attention).  Severe adverse events that may occur during the CM intervention include death and cardiovascular events, though these are extremely rare in the absence of significant cardiac pathology. Other serious adverse events include suicidal ideation and/or self-injurious behaviors. For these adverse events, participants will be referred to the nearest emergency room.
2. Examples of moderate adverse events (resolve with treatment).  Moderate adverse events include any new psychiatric symptoms or side effects related to the use of cessation medications (e.g. nightmares for NRT, seizures for bupropion, new depressive symptoms for varenicline). For these events, we will refer the participants to their primary care providers.
3. Examples of mild adverse events (ones that do not require treatment).  Mild adverse effects may be symptoms of irritability or fatigue or hunger from stopping smoking. Participants will be offered information on these symptoms prior to the onset of the study and during the study. Participants will be given a list of resources, including food pantries, and other locations that offer free food.

# 3.  Description of Monitoring Study Progress and Safety of Human Subject Participants

The PI has primary responsibility for the overall conduct of the study and for the safety of participants. The PI will ensure that (1) the informed consent process is conducted appropriately and that informed consent is obtained prior to proceeding with any study procedures; (2) only eligible subjects, per protocol eligibility criteria, are enrolled in the study; (3) data are collected and analyzed per protocol requirements; (4) procedures are implemented to ensure that the project is consistently monitored for possible adverse events; (5) adverse events are reviewed promptly and reported as required to the UCSF Committee on Human Research (i.e. UCSF Institutional Review Board); (6) the privacy and confidentiality of study subjects is maintained.  While implementation of aspects of the DSMP may be delegated to members of the research team, the PI maintains ultimate responsibility for the project and for the safety of study participants.

The co-investigators, Dr. Margot Kushel, and Dr. Sharon Hall, along with the statistician Dr. Steven Gregorich, and other research team members (TBD, study staff) will participate in the monitoring of the safety of study participants together with the PI.  Every six months, the PI, the statistician, and study staff will prepare a written report on the progress of the project including data on: enrollments, comparison of target to actual enrollment, overall status of the study participants, information on race/ethnicity, age and gender, adverse events, and serious adverse events. The PI and the research team will meet to review information contained in the reports.  During the reviews, the PI and research team will determine whether additional effort is required to foster the progress of the study, whether adverse events have occurred, whether adverse events were dealt with appropriately, and whether adverse events were corrected and immediately reported to the UCSF Committee on Human Research. The PI and team will determine whether the study should be continued, terminated, or modified based on observed beneficial or adverse effects.

# 4.  Plans for Assuring Compliance with Requirements Regarding the Reporting of Adverse Events

The PI is responsible for reporting adverse events to the research team and to the UCSF Committee on Human Research. Mild and moderate adverse events will be reported within 10 working days of the occurrence. Severe, life-threatening or fatal adverse events will be reported immediately to the PI, who will notify the UCSF Committee on Human Research (CHR) within 24 hours by telephone. An expedited report will follow within 10 days. Specifically, the following will be reported, in writing: 1) all severe adverse events associated with the study procedures, and/or 2) any incidents or problems involving the conduct of the study or patient participation, including problems with the recruitment and/or consent processes and/or education of participants about safety. The PI will provide a written report of moderate adverse events during the course of the study to the CHR on an annual basis.

When the PI receives a report of a severe or greater adverse event, she will immediately notify all members of the research team.  A meeting will be held within ten days to discuss whether study procedures should be modified.  Moderate and mild adverse events will be tabulated by the PI and study staff, who will produce reports of adverse events every six months. The research team will review reports to identify any potential trends. If trends are noted, preventive measures will be implemented, such as changing the education of participants in the study to emphasize prevention of the adverse event. This means that study staff have been trained to recognize, respond to, and record adverse events when they occur or immediately after they occur to insure the safety of the human subjects; and to report adverse events to the PI in a timely manner to ensure compliance with institutional policies on human subject protection. This also means that study staff engaged in data collection can contact the PI as soon as an adverse event occurs.

Cardiac events or deaths are very rare in persons engaging in smoking cessation interventions. We will stop the study based on instructions from the UCSF CHR, which will be notified immediately if a participant in this study experiences a severe adverse event while enrolled in the study. Because the proposed study entails minimal risk, we do not anticipate that a DSMB will be required.  However, if the funding agency requests a DSMB, we will work with them to form one.

1. Informed consent must be obtained prior to any study-specific procedures and may be obtained prior to the screening window.

   ^2^ Participants will be asked to provide an expired carbon monoxide sample at each abstinence assessment visit, and complete a short questionnaire. At 2 weeks, 1 months, 3 months, 6 months and 1 year follow-up, participants will also be required to complete a longer questionnaire.

   ^3^ Between 6 months and 1 year follow-up, participants will be asked to check in by telephone monthly. [↑](#footnote-ref-2)
