# Supplementary material for: Contingency management to promote smoking cessation in people experiencing homelessness: Leveraging the electronic health record in a pilot, pragmatic randomized controlled trial
Source: PLoS One. 2022 Dec 16;17(12):e0278870. doi: 10.1371/journal.pone.0278870 (PMC9757562; doi:10.1371/journal.pone.0278870)
Supplement: S2 File — (PDF) [file pone.0278870.s003.pdf]

Data Dictionary Codebook

#	Variable / Field Name	Field Label<br>*Field Note*	Field Attributes (Field Type, Validation, Choices, Calculations, etc.)
Instrument: **RCT Participant Survey- Baseline** (rct_participant_survey_baseline)			
1	[participant_id]	Participant ID	text
2	[study_id]	Study ID	text, Required
3	[recruiter]	Section Header: *Screening Questions*<br>Recruiter name	radio<br>1 Recruiter X (Jordan)<br>2 Recruiter Y (Phoebe or Jessica)
4	[screen_pregnancy]	Are you currently pregnant?	yesno, Required<br>1 Yes<br>0 No<br>Stop actions on 1
5	[screen_heartattack]	Have you had a myocardial infarction (heart attack) in the past two weeks?	yesno, Required<br>1 Yes<br>0 No
6	[screen_100cigs]	Have you smoked at least 100 cigarettes in your lifetime?	yesno, Required<br>1 Yes<br>0 No
7	[screen_5cigs]	Do you currently smoke at least 5 cigarettes per day, every day?	yesno, Required<br>1 Yes<br>0 No
8	[screen_firstcig]	How soon after you wake up to you have your first cigarette?	radio, Required<br>1 Within 30 minutes<br>2 After 30 minutes
9	[screen_meduse]	Are you willing to use medication and receive counseling to help with smoking cessation at your clinic?	yesno, Required<br>1 Yes<br>0 No
10	[screen_time]	Are you able to meet with study staff frequently for the next 6 months?	yesno, Required<br>1 Yes<br>0 No
11	[screen_homeless]	Are you currently homeless (on the streets, in a shelter, in a short term SRO, staying with a friend or family member, etc.)?	yesno, Required<br>1 Yes<br>0 No
12	[screen_insurance]	Do you currently have health insurance?	yesno, Required<br>1 Yes<br>0 No

13	[screen_insuranceprovider]<br>Show the field ONLY if:<br>[screen_insurance] = '1'	Which insurance provider do you have?	radio<br>1 San Francisco Health Plan Medi-Cal<br>2 Anthem Blue Cross Medi-Cal<br>3 MediCare<br>4 Healthy San Francisco<br>5 Healthy Workers<br>6 Private insurance<br>7 Other Medi-Cal/Medicaid<br>8 None<br>9 Other (please specify): ____________________
14	[other_ins]<br>Show the field ONLY if:<br>[screen_insuranceprovider] = '9'	If other, please explain	text
15	[prescribed_medication]	Are you currently taking Wellbutrin or Zyban?	yesno, Required<br>1 Yes<br>0 No
16	[nrt]	Are you currently taking NRT?	yesno, Required<br>1 Yes<br>0 No
17	[residence]	Do you plan to reside in the San Francisco Bay Area for the next 12 months?	yesno, Required<br>1 Yes<br>0 No
18	[current_date]	Section Header: *Please fill out the information below.*<br>Current Date	text (date_mdy), Required
19	[first_name]	First Name	text, Required
20	[last_name]	Last Name	text, Required
21	[dob]	Date of birth	text (date_mdy)
22	[email]	E-mail address	text (email), Identifier
23	[phone_number]	Phone Number	text, Required
24	[medical_care_loc]	Location of Medical Care	text, Required
25	[pcp]	Name of Primary Care Provider	text, Required
26	[lodging]	Where did you sleep last night?	text, Required
27	[consent]	By checking this box, I certify that I am at least 18 years old and that I give my consent freely to participant in this study.	checkbox<br>1 consent___1 I consent
28	[co_reading]	Carbon Monoxide reading	text (number)
29	[treatment_assignment]	Treatment assignment number	dropdown<br>101 101<br>102 102<br>103 103<br>104 104<br>105 105<br>106 106<br>107 107<br>108 108<br>109 109<br>110 110<br>111 111<br>112 112

113	113
114	114
115	115
116	116
117	117
118	118
119	119
120	120
121	121
122	122
123	123
124	124
125	125
126	126
127	127
128	128
129	129
130	130
131	131
132	132
133	133
134	134
135	135
136	136
137	137
138	138
139	139
140	140
141	141
142	142
143	143
144	144
145	145
146	146
147	147
148	148
149	149
150	150
151	151
152	152
153	153
154	154
155	155
156	156
157	157
158	158
159	159
160	160

161	161
162	162
163	163
164	164
165	165
166	166
167	167
168	168
169	169
170	170
171	171
172	172
173	173
174	174
175	175
176	176
177	177
178	178
179	179
180	180
181	181
182	182
183	183
184	184
185	185
186	186
187	187
188	188
189	189
190	190
191	191
192	192
193	193
194	194
195	195
196	196
197	197
198	198
199	199
200	200
201	201
202	202
203	203
204	204
205	205
206	206
207	207
208	208

209	209
210	210
211	211
212	212
213	213
214	214
215	215
216	216
217	217
218	218
219	219
220	220
221	221
222	222
223	223
224	224
225	225
226	226
227	227
228	228
229	229
230	230
231	231
232	232
233	233
234	234
235	235
236	236
237	237
238	238
239	239
240	240
241	241
242	242
243	243
244	244
245	245
246	246
247	247
248	248
249	249
250	250
251	251
252	252
253	253
254	254
255	255
256	256

257	257
258	258
259	259
260	260
261	261
262	262
263	263
264	264
265	265
266	266
267	267
268	268
269	269
270	270
271	271
272	272
273	273
274	274
275	275
276	276
277	277
278	278
279	279
280	280
281	281
282	282
283	283
284	284
285	285
286	286
287	287
288	288
289	289
290	290
291	291
292	292
293	293
294	294
295	295
296	296
297	297
298	298
299	299
300	300
301	301
302	302
303	303
304	304

305	305
306	306
307	307
308	308
309	309
310	310
311	311
312	312
313	313
314	314
315	315
316	316
317	317
318	318
319	319
320	320
321	321
322	322
323	323
324	324
325	325
326	326
327	327
328	328
329	329
330	330
331	331
332	332
333	333
334	334
335	335
336	336
337	337
338	338
339	339
340	340
341	341
342	342
343	343
344	344
345	345
346	346
347	347
348	348
349	349
350	350
351	351
352	352

353	353
354	354
355	355
356	356
357	357
358	358
359	359
360	360
361	361
362	362
363	363
364	364
365	365
366	366
367	367
368	368
369	369
370	370
371	371
372	372
373	373
374	374
375	375
376	376
377	377
378	378
379	379
380	380
381	381
382	382
383	383
384	384
385	385
386	386
387	387
388	388
389	389
390	390
391	391
392	392
393	393
394	394
395	395
396	396
397	397
398	398
399	399
400	400

401	401
402	402
403	403
404	404
405	405
406	406
407	407
408	408
409	409
410	410
411	411
412	412
413	413
414	414
415	415
416	416
417	417
418	418
419	419
420	420
421	421
422	422
423	423
424	424
425	425
426	426
427	427
428	428
429	429
430	430
431	431
432	432
433	433
434	434
435	435
436	436
437	437
438	438
439	439
440	440
441	441
442	442
443	443
444	444
445	445
446	446
447	447
448	448

449	449
450	450
451	451
452	452
453	453
454	454
455	455
456	456
457	457
458	458
459	459
460	460
461	461
462	462
463	463
464	464
465	465
466	466
467	467
468	468
469	469
470	470
471	471
472	472
473	473
474	474
475	475
476	476
477	477
478	478
479	479
480	480
481	481
482	482
483	483
484	484
485	485
486	486
487	487
488	488
489	489
490	490
491	491
492	492
493	493
494	494
495	495
496	496

497	497
498	498
499	499
500	500
501	501
502	502
503	503
504	504
505	505
506	506
507	507
508	508
509	509
510	510
511	511
512	512
513	513
514	514
515	515
516	516
517	517
518	518
519	519
520	520
521	521
522	522
523	523
524	524
525	525
526	526
527	527
528	528
529	529
530	530
531	531
532	532
533	533
534	534
535	535
536	536
537	537
538	538
539	539
540	540
541	541
542	542
543	543
544	544

545	545
546	546
547	547
548	548
549	549
550	550
551	551
552	552
553	553
554	554
555	555
556	556
557	557
558	558
559	559
560	560
561	561
562	562
563	563
564	564
565	565
566	566
567	567
568	568
569	569
570	570
571	571
572	572
573	573
574	574
575	575
576	576
577	577
578	578
579	579
580	580
581	581
582	582
583	583
584	584
585	585
586	586
587	587
588	588
589	589
590	590
591	591
592	592

593	593
594	594
595	595
596	596
597	597
598	598
599	599
600	600
601	601
602	602
603	603
604	604
605	605
606	606
607	607
608	608
609	609
610	610
611	611
612	612
613	613
614	614
615	615
616	616
617	617
618	618
619	619
620	620
621	621
622	622
623	623
624	624
625	625
626	626
627	627
628	628
629	629
630	630
631	631
632	632
633	633
634	634
635	635
636	636
637	637
638	638
639	639
640	640

641	641
642	642
643	643
644	644
645	645
646	646
647	647
648	648
649	649
650	650
651	651
652	652
653	653
654	654
655	655
656	656
657	657
658	658
659	659
660	660
661	661
662	662
663	663
664	664
665	665
666	666
667	667
668	668
669	669
670	670
671	671
672	672
673	673
674	674
675	675
676	676
677	677
678	678
679	679
680	680
681	681
682	682
683	683
684	684
685	685
686	686
687	687
688	688

689	689
690	690
691	691
692	692
693	693
694	694
695	695
696	696
697	697
698	698
699	699
700	700
701	701
702	702
703	703
704	704
705	705
706	706
707	707
708	708
709	709
710	710
711	711
712	712
713	713
714	714
715	715
716	716
717	717
718	718
719	719
720	720
721	721
722	722
723	723
724	724
725	725
726	726
727	727
728	728
729	729
730	730
731	731
732	732
733	733
734	734
735	735
736	736

737	737
738	738
739	739
740	740
741	741
742	742
743	743
744	744
745	745
746	746
747	747
748	748
749	749
750	750
751	751
752	752
753	753
754	754
755	755
756	756
757	757
758	758
759	759
760	760
761	761
762	762
763	763
764	764
765	765
766	766
767	767
768	768
769	769
770	770
771	771
772	772
773	773
774	774
775	775
776	776
777	777
778	778
779	779
780	780
781	781
782	782
783	783
784	784

785	785
786	786
787	787
788	788
789	789
790	790
791	791
792	792
793	793
794	794
795	795
796	796
797	797
798	798
799	799
800	800
801	801
802	802
803	803
804	804
805	805
806	806
807	807
808	808
809	809
810	810
811	811
812	812
813	813
814	814
815	815
816	816
817	817
818	818
819	819
820	820
821	821
822	822
823	823
824	824
825	825
826	826
827	827
828	828
829	829
830	830
831	831
832	832

833	833
834	834
835	835
836	836
837	837
838	838
839	839
840	840
841	841
842	842
843	843
844	844
845	845
846	846
847	847
848	848
849	849
850	850
851	851
852	852
853	853
854	854
855	855
856	856
857	857
858	858
859	859
860	860
861	861
862	862
863	863
864	864
865	865
866	866
867	867
868	868
869	869
870	870
871	871
872	872
873	873
874	874
875	875
876	876
877	877
878	878
879	879
880	880

			881 881<br>882 882<br>883 883<br>884 884<br>885 885<br>886 886<br>887 887<br>888 888<br>889 889<br>890 890<br>891 891<br>892 892<br>893 893<br>894 894<br>895 895<br>896 896<br>897 897<br>898 898<br>899 899<br>900 900
30	[smoker_frequency]	Section Header: *Cigarette Use and Smoking History*<br>How often do you smoke?	radio, Required<br>1 Everyday<br>2 Somedays
31	[menthol]	Do you usually smoke menthols or non-menthols?	radio, Required<br>1 Menthols<br>2 Non-Menthols
32	[timetofirstcig]	If there were no restrictions on you, how soon after you woke up would you have your first cigarette?	radio, Required<br>1 Within 5 minutes<br>2 6-30 minutes<br>3 31-60 minutes<br>4 After 60 minutes
33	[cessation_intention]	What best describes your intention to quit smoking?	radio<br>1 I never expect to quit<br>2 I may quit<br>3 I will quit in the next 6 months<br>4 I will quit in the next month
34	[doctor_recommendation]	Within the past 12 months, has a doctor or other healthcare provider recommended that you stop smoking?	yesno, Required<br>1 Yes<br>0 No
35	[quit_attempts]	During the past 12 months, have you stopped smoking for one day or longer because you were trying to stop smoking?	yesno, Required<br>1 Yes<br>0 No
36	[lengthofquit]<br>Show the field ONLY if:<br>[quit_attempts] = '1'	How long were you able to go without smoking during your last quit attempt (number of days) in the past 12 months?	text (number, Min: 1, Max: 365), Required

37	[cessationaids]<br><br>Show the field ONLY if:<br>[quit_attempts] = '1'	What products, methods or resources did you use to help you stop smoking in the past year (check all that apply, or "never tried to quit")?	checkbox, Required<br>1 cessationaids___1 Quit "cold turkey"<br>2 cessationaids___2 Gradually cut down<br>3 cessationaids___3 Smoking cessation class<br>4 cessationaids___4 Advice from a healthcare professional (doctor, nurse, psychologist, etc.)<br>5 cessationaids___5 Advice from shelter staff<br>6 cessationaids___6 Called a telephone hotline<br>7 cessationaids___7 Hypnosis<br>8 cessationaids___8 Acupuncture<br>9 cessationaids___9 Nicotine gum<br>10 cessationaids___10 Nicotine patch<br>11 cessationaids___11 Nicotine spray<br>12 cessationaids___12 Nicotine lozenge<br>13 cessationaids___13 Nicotine inhaler<br>14 cessationaids___14 Zyban/Wellbutrin for smoking cessation<br>15 cessationaids___15 Chantix/Varenicline for smoking cessation<br>16 cessationaids___16 E-cigarettes to help cut down or stop using regular cigarettes<br>17 cessationaids___17 Smokeless tobacco<br>18 cessationaids___18 Cannabis or marijuana to help cut down or stop using regular cigarettes<br>19 cessationaids___19 Other<br>20 cessationaids___20 Never tried to quit
38	[other_nrt]<br><br>Show the field ONLY if:<br>[cessationaids(18)] = '1'	If other, please explain	text, Required
39	[past7dayssmk]	Did you smoke any cigarettes in the past 7 days?	yesno, Required<br>1 Yes<br>0 No
40	[noofdayssmk_past7]<br><br>Show the field ONLY if:<br>[past7dayssmk] = '1'	Out of the past 7 days, on how many days did you smoke cigarettes?	text (number, Min: 1, Max: 7), Required
41	[noofcigssmk]<br><br>Show the field ONLY if:<br>[past7dayssmk] = '1'	In the past 7 days, on the days that you did smoke, about how many cigarettes did you usually smoke per day?	text (number, Min: 1), Required
42	[smkinitiation]	How old were you when you first started smoking cigarettes fairly regularly?	text (number), Required
43	[ecig_everuse]	Section Header: *I'm going to ask you about products you may or may not have used. Please answer "yes" if you have ever tried the product, even just once in your life.*<br><br>Have you ever used e-cigarettes, even one or two puffs?	yesno, Required<br>1 Yes<br>0 No
44	[ecig_freq]<br><br>Show the field ONLY if:<br>[ecig_everuse] = '1'	During your entire life, about how frequently have you used e-cigarettes?	radio, Required<br>1 Only once<br>2 A few times in my life<br>3 Most of the time<br>4 Always

45	[ecig_past30]<br><br>Show the field ONLY if:<br>[ecig_everuse] = '1'	Have you used an e-cigarette in the past 30 days?	yesno, Required<br>1 Yes<br>0 No
46	[ecig_nodays]<br><br>Show the field ONLY if:<br>[ecig_past30] = '1'	In the past 30 days, how many days did you use an e-cigarette?	text (number, Min: 1, Max: 30), Required
47	[ecig_timeofuse]<br><br>Show the field ONLY if:<br>[ecig_past30] = '1'	How many times per day do you usually use your e-cigarette / vape pen (assume that one "time" consists of around 15 puffs or lasts around 10 minutes)?	text (number, Min: 1), Required
48	[ecig_nicotine]<br><br>Show the field ONLY if:<br>[ecig_past30] = '1'	What concentration of nicotine do you usually use in your e-cigarette?	radio, Required<br>1 1-6 mg<br>2 7-12 mg<br>3 13-18 mg<br>4 19-24 mg<br>5 25< + mg<br>6 Don't know
49	[ecig_patternuse]<br><br>Show the field ONLY if:<br>[ecig_past30] = '1'	How do you usually smoke your e-cigarette / vapor pen?	radio, Required<br>1 Continuously throughout the day (only a few puffs each time)<br>2 Distinct smoking bouts that are shorter than smoking a traditional cigarette (shorter length of time and fewer number of puffs)<br>3 Distinct smoking bouts similar to smoking a traditional cigarette (similar length of time and number of puffs)<br>4 Other
50	[ecig_patternuseother]<br><br>Show the field ONLY if:<br>[ecig_patternuse] = '4'	If other, please explain	text
51	[ecig_timeusage]<br><br>Show the field ONLY if:<br>[ecig_past30] = '1'	During the past 30 days, on the days when you used e-cigarettes, about how much time did you use each day?	radio, Required<br>1 Just a few puffs, less than 1 minute total<br>2 1-5 minutes total<br>3 6-15 minutes total<br>4 16-60 minutes total<br>5 More than 1 hour total use

52	[ecig_motivation]<br><br>Show the field ONLY if:<br>[ecig_past30] = '1'	What best describes your motivation to use e-cigarettes?	checkbox<br>1 ecig_motivation___1 E-cigarettes help me to quit smoking<br>2 ecig_motivation___2 E-cigarettes help me to cut down on smoking<br>3 ecig_motivation___3 E-cigarettes are cheaper than smoking cigarettes<br>4 ecig_motivation___4 E-cigarettes are less harmful to my health than cigarettes<br>5 ecig_motivation___5 E-cigarettes can be used in places where cigarettes are not allowed<br>6 ecig_motivation___6 E-cigarettes taste better and are more pleasurable to use than cigarettes<br>7 ecig_motivation___7 I use this product for reasons not listed<br>8 ecig_motivation___8 I am trying to stop smoking cigarettes<br>9 ecig_motivation___9 Other<br>10 ecig_motivation___10 None of the above
53	[ecig_motivationother]<br><br>Show the field ONLY if:<br>[ecig_motivation(9)] = '1'	If other, please explain	text, Required
54	[smkless_everuse]	Have you ever used smokeless tobacco (chewing or dipping tobacco, moist snuff, or SNUs), even one or two puffs?	yesno<br>1 Yes<br>0 No
55	[smkless_freq]<br><br>Show the field ONLY if:<br>[smkless_everuse] = '1'	During your entire life, about how frequently have you used smokeless tobacco (chewing or dipping tobacco, moist snuff, or SNUS)?	radio, Required<br>1 Only once<br>2 A few times in my life<br>3 Most of the time<br>4 Always
56	[smkless_past30]<br><br>Show the field ONLY if:<br>[smkless_everuse] = '1'	Have you used smokeless tobacco in the past 30 days?	yesno, Required<br>1 Yes<br>0 No
57	[smkless_noofdays]<br><br>Show the field ONLY if:<br>[smkless_past30] = '1'	In the past 30 days, how many days did you use smokeless tobacco?	text (number, Min: 1, Max: 30), Required
58	[cigars_everuse]	Have you ever used cigars, cigarillos, or little cigars, even one or two puffs?	yesno<br>1 Yes<br>0 No
59	[cigar_use]<br><br>Show the field ONLY if:<br>[cigars_everuse] = '1'	During your entire life, about how frequently have you used cigars, cigarillos, or little cigars?	radio<br>1 Only once<br>2 A few times in my life<br>3 Most of the time<br>4 Always
60	[cigar_past30]<br><br>Show the field ONLY if:<br>[cigars_everuse] = '1'	Have you used cigars, cigarillos, or little cigars in the past 30 days	yesno, Required<br>1 Yes<br>0 No

61	[cigar_nodays]<br>Show the field ONLY if:<br>[cigar_past30] = '1'	In the last 30 days, on about how many days did you smoke cigars, cigarillos or little cigars?	text (number, Min: 1, Max: 30), Required
62	[pipe_everuse]	Have you ever smoked a tobacco pipe, even one or two puffs?	yesno<br>1 Yes<br>0 No
63	[pipe_freq]<br>Show the field ONLY if:<br>[pipe_everuse] = '1'	During your entire life, about how frequently have you smoked tobacco pipes?	radio, Required<br>1 Only once<br>2 A few times in my life<br>3 Most of the time<br>4 Always
64	[pipe_past30]<br>Show the field ONLY if:<br>[pipe_everuse] = '1'	Have you used a tobacco pipe in the past 30 days?	yesno, Required<br>1 Yes<br>0 No
65	[pipe_nodays]<br>Show the field ONLY if:<br>[pipe_past30] = 1	In the last 30 days, on about how many days did you smoke a tobacco pipe?	text (number, Min: 1, Max: 30), Required
66	[hookah_everuse]	Have you ever used a hookah, even one or two puffs?	yesno<br>1 Yes<br>0 No
67	[hookah_freq]<br>Show the field ONLY if:<br>[hookah_everuse] = '1'	During your entire life, about how frequently have you used a hookah?	radio, Required<br>1 Only once<br>2 A few times in my life<br>3 Most of the time<br>4 Always
68	[hookah_past30]<br>Show the field ONLY if:<br>[hookah_everuse] = '1'	Have you used a hookah in the past 30 days?	yesno, Required<br>1 Yes<br>0 No
69	[hookah_nodays]<br>Show the field ONLY if:<br>[hookah_past30] = '1'	In the last 30 days, on about how many days did you smoke from a hookah?	text (number, Min: 1, Max: 30), Required
70	[marijuana_everuse]	Have you ever used marijuana (cannabis) or hashish, even one or two puffs?	yesno<br>1 Yes<br>0 No
71	[marijuana_freq]<br>Show the field ONLY if:<br>[marijuana_everuse] = '1'	During your entire life, about how frequently have you used marijuana (cannabis) or hashish?	radio, Required<br>1 Only once<br>2 A few times in my life<br>3 Most of the time<br>4 Always
72	[marijuana_past30]<br>Show the field ONLY if:<br>[marijuana_everuse] = '1'	Have you used marijuana (cannabis) or hashish in the past 30 days?	yesno, Required<br>1 Yes<br>0 No
73	[marijuana_nodays]<br>Show the field ONLY if:<br>[marijuana_past30] = '1'	In the last 30 days, on about how many days did you use marijuana (cannabis) or hashish?	text (number, Min: 1, Max: 30), Required
74	[blunts_everuse]	Have you ever used a blunt (marijuana rolled in a tobacco leaf), even one or two puffs?	yesno<br>1 Yes<br>0 No

75	[blunts_freq]<br><br>Show the field ONLY if:<br>[blunts_everuse] = '1'	During your entire life, about how frequently have you used a blunt?	radio, Required<br>1 Only once<br>2 A few times in my life<br>3 Most of the time<br>4 Always
76	[blunts_past30]<br><br>Show the field ONLY if:<br>[blunts_everuse] = '1'	Have you used blunts in the past 30 days?	yesno, Required<br>1 Yes<br>0 No
77	[blunts_nodays]<br><br>Show the field ONLY if:<br>[blunts_past30] = '1'	In the last 30 days, on about how many days did you use blunts?	text (number, Min: 1, Max: 30), Required
78	[cannabismotivations]<br><br>Show the field ONLY if:<br>[marijuana_everuse] = '1'	What are some of the reasons for your using marijuana (cannabis) or hashish? (Mark all that apply.)	checkbox, Required<br>1 cannabismotivations___1 To experiment - to see what it's like<br>2 cannabismotivations___2 To relax or relieve tension<br>3 cannabismotivations___3 To feel good or get high<br>4 cannabismotivations___4 To seek deeper insights and understanding<br>5 cannabismotivations___5 To have a good time with my friends<br>6 cannabismotivations___6 To fit in with a group I like<br>7 cannabismotivations___7 To get away from my problems or troubles<br>8 cannabismotivations___8 To relieve of boredom, nothing else to do<br>9 cannabismotivations___9 To release of anger or frustration<br>10 cannabismotivations___10 To get through the day<br>11 cannabismotivations___11 To increase the effects of some other drug(s)<br>12 cannabismotivations___12 To decrease (offset) the effects of some other drug(s)<br>13 cannabismotivations___13 To get to sleep<br>14 cannabismotivations___14 It tastes good<br>15 cannabismotivations___15 I am "hooked"- I feel I have to have it<br>16 cannabismotivations___16 To quit or cut down on smoking cigarettes<br>17 cannabismotivations___17 Other
79	[other_cannamotiv]<br><br>Show the field ONLY if:<br>[cannabismotivations(17)] = '1'	If other, please explain	text

80	[medmarijuana_card]	Do you currently have a medical marijuana card?	yesno, Required<br>1 Yes<br>0 No
81	[tobacco_sources]	Where do you usually get your tobacco products? Please tell us your most common sources	checkbox<br>1 tobacco_sources___1 From a liquor store, convenience store or gas station<br>2 tobacco_sources___2 From a supermarket or drug store<br>3 tobacco_sources___3 From a smoke shop, vape shop or marijuana dispensary<br>4 tobacco_sources___4 From friends or strangers (Please check all that apply.)
82	[easy_purchase]	How easy was it to get your typical tobacco products in the past 30 days?	radio, Required<br>1 Very difficult<br>2 Somewhat difficult<br>3 Somewhat easy<br>4 Very easy
83	[audit7_freq]	Section Header: *Alcohol Use. Please answer the following questions regarding alcohol use.*<br>How often do you have a drink containing alcohol?	radio<br>1 Never<br>2 Monthly or less<br>3 2-4 times a month<br>4 2-3 times a week<br>5 4 or more times a week
84	[audit7alcohol_quantity]<br>Show the field ONLY if: [audit7_freq] = '2' or [audit7_freq] = '3' or [audit7_freq] = '4' or [audit7_freq] = '5'	How many standard drinks containing alcohol do you have on a typical day?	radio, Required<br>0 0<br>1 1 to 2<br>2 3 to 4<br>3 5 to 6<br>4 7 to 9<br>5 10 or more
85	[audit7alcohol_intake]<br>Show the field ONLY if: [audit7_freq] = '2' or [audit7_freq] = '3' or [audit7_freq] = '4' or [audit7_freq] = '5'	How often do you have six or more drinks on one occasion	radio, Required<br>1 Never<br>2 Less than monthly<br>3 Monthly<br>4 Weekly<br>5 Daily or almost daily
86	[audit7gender_intake]<br>Show the field ONLY if: [audit7_freq] = '2' or [audit7_freq] = '3' or [audit7_freq] = '4' or [audit7_freq] = '5'	During the past 30 days, on how many days did you drink at least 4 or more alcoholic drinks (if you are a woman) OR 5 or more alcoholic drinks (if you are a man)?	radio, Required<br>1 Never<br>2 1-3 times this month<br>3 1-2 times a week<br>4 3 or more times a week

87	[cocaine_everuse]	Section Header: *This next section asks about your use of drugs. I am going to ask you some questions about your experience of using different substances across your lifetime and in the past 6 months. These substances can be smoked, swallowed, snorted, inhaled, injected or taken in the form of pills. Some substances listed may be prescribed by a doctor (like amphetamines, sedatives, pain medications). For this interview, we will NOT record medications that are used AS PRESCRIBED by your doctor. However, if you have taken such medications for reasons other than prescription, or taken them more frequently or at higher doses than prescribed, please let me know. While we are also interested in knowing about your use of various illicit drugs, please be assured that information on such use will be treated strictly confidential.*<br>Have you ever used cocaine (coke, crack, etc.)?	yesno<br>1 Yes<br>0 No
88	[cocaine_past30]<br>Show the field ONLY if:<br>[cocaine_everuse] = '1'	Have you used cocaine in the past 30 days?	yesno, Required<br>1 Yes<br>0 No
89	[cocaine_nodays]<br>Show the field ONLY if:<br>[cocaine_past30] = '1'	In the past 30 days, how many days did you use Cocaine, or Crack?	text (number, Min: 1, Max: 30), Required
90	[amphetamines_everuse]	Have you ever used amphetamine type stimulants (speed, meth, crystal, diet pills, ecstasy, methamphetamine, ice, etc.)?	yesno, Required<br>1 Yes<br>0 No
91	[amphetamines_past30]<br>Show the field ONLY if:<br>[amphetamines_everuse] = '1'	Have you used amphetamines in the past 30 days?	yesno<br>1 Yes<br>0 No
92	[amphetamines_nodays]<br>Show the field ONLY if:<br>[amphetamines_past30] = '1'	In the last 30 days, how many days have you used amphetamines?	text (number, Min: 1, Max: 30), Required
93	[inhalants_everuse]	Have you ever in your life used any inhalants (poppers, nitrous, glue, petrol/gasoline, paint thinner) ?	yesno, Required<br>1 Yes<br>0 No
94	[inhalants_past30]<br>Show the field ONLY if:<br>[inhalants_everuse] = '1'	Have you used any inhalants in the last 30 days?	yesno<br>1 Yes<br>0 No
95	[inhalants_nodays]<br>Show the field ONLY if:<br>[inhalants_past30] = '1'	In the last 30 days, how many days did you use inhalants?	text (number, Min: 1, Max: 30), Required
96	[sedatives_everuse]	Have you ever in your life used any sedatives or sleeping pills recreationally/ that were not prescribed to you (Valium, Klonopin, Xanax, Serepax, Rohypnol, Seconal, Ativan, Phenergan, Ambien, etc.)?	yesno, Required<br>1 Yes<br>0 No
97	[sedative_past30]<br>Show the field ONLY if:<br>[sedatives_everuse] = '1'	Have you used any sedatives or sleeping pills in the last 30 days?	yesno, Required<br>1 Yes<br>0 No
98	[sedatives_nodays]<br>Show the field ONLY if:<br>[sedative_past30] = '1'	In the last 30 days, how many days have you used sleeping pills or sedatives?	text (number, Min: 1, Max: 30), Required
99	[hallucinogens_everuse]	Have you ever in your life used any Hallucinogens (LSD, Acid, mushrooms, PCP, psilocybin, Angel Dust, Special K, etc.)?	yesno<br>1 Yes<br>0 No
100	[hallucinogens_past30]<br>Show the field ONLY if:<br>[hallucinogens_everuse]	Have you used any hallucinogens in the last 30 days?	yesno, Required<br>1 Yes<br>0 No
101	[hallucinogens_nodays]<br>Show the field ONLY if:<br>[hallucinogens_past30] = '1'	In the last 30 days, how many days did you use hallucinogens?	text (number, Min: 1, Max: 30), Required

102	[opioids_everuse]	Have you ever in your life used any opioids recreationally that were not prescribed to you (Heroin, morphine, methadone, Vicodin, Codeine, Demerol, Dilaudid, hydromorphone, fentanyl, oxycodone, Oxycontin, MSContin, etc.)?	yesno<br>1 Yes<br>0 No
103	[opiods_past30]<br>Show the field ONLY if:<br>[opioids_everuse] = '1'	Have you used any opioids in the last 30 days?	yesno, Required<br>1 Yes<br>0 No
104	[opioids_nodays]<br>Show the field ONLY if:<br>[opiods_past30] = '1'	In the last 30 days, how many days did you use opioids?	text (number, Min: 1, Max: 30), Required
105	[otherdrugs_everuse]	Have you ever in your life used any other drugs (GHB, Kava, Khat, ect.)?	yesno<br>1 Yes<br>0 No
106	[otherdrugs_past30]<br>Show the field ONLY if:<br>[otherdrugs_everuse] = '1'	Have you used any other drugs in the last 30 days?	yesno<br>1 Yes<br>0 No
107	[otherdrugs_nodays]<br>Show the field ONLY if:<br>[otherdrugs_past30] = '1'	In the last 30 days, how many days did you use any other drugs?	text (number, Min: 1, Max: 30), Required
108	[health_rating]	Section Header: *Health History: Please answer these questions about your health history*<br>In general, would you say your overall health is?	radio, Required<br>1 Excellent<br>2 Very good<br>3 Good<br>4 Fair<br>5 Poor<br>6 Very poor
109	[hypertension]	Has a doctor or other healthcare provider ever told you that you had hypertension or high blood pressure?	yesno, Required<br>1 Yes<br>0 No
110	[heart_failure]	Has a doctor or other healthcare provider ever told you that you had congestive heart failure?	yesno, Required<br>1 Yes<br>0 No
111	[heart_disease]	Has a doctor or other healthcare provider ever told you that you had coronary artery disease or angina pectoris?	yesno<br>1 Yes<br>0 No
112	[stroke]	Has a doctor or other healthcare provider ever told you that you had a stroke, or TIA or mini-stroke?	yesno, Required<br>1 Yes<br>0 No
113	[arthritis]	Has a doctor or other healthcare provider ever told you that you had arthritis?	yesno, Required<br>1 Yes<br>0 No
114	[breathing]	Has a doctor or other healthcare provider ever told you that you had emphysema, chronic bronchitis or COPD (chronic obstructive pulmonary disease)?	yesno, Required<br>1 Yes<br>0 No
115	[liver]	Has a doctor or other healthcare provider ever told you that you had any kind of liver problem, such as cirrhosis or chronic hepatitis?	yesno, Required<br>1 Yes<br>0 No

116	[diabetes]	Has a doctor or other healthcare provider ever told you that you had diabetes, other than during pregnancy?	yesno, Required<br>1 Yes<br>0 No
117	[asthma]	Has a doctor or other healthcare provider ever told you that you had asthma?	yesno, Required<br>1 Yes<br>0 No
118	[renal]	Has a doctor or other healthcare provider ever told you that you had weak or failing kidneys? Do not include kidney stones, bladder infections, or incontinence.	yesno, Required<br>1 Yes<br>0 No
119	[cancer]	Have you ever been told by a doctor or other health professional that you had cancer or a malignancy of any kind?	radio, Required<br>1 yes<br>2 no
120	[cancer_diagnosis]<br>Show the field ONLY if:<br>[cancer] = '1'	What kinds of Cancer have you had?	checkbox, Required<br>1 cancer_diagnosis___1 Bladder Blood Bone Brain Breast<br>2 cancer_diagnosis___2 Cervix (Cervical) Colon<br>3 cancer_diagnosis___3 Esophagus (Esophageal) Gallbladder<br>4 cancer_diagnosis___4 Kidney Larynx/Windpipe Leukemia<br>5 cancer_diagnosis___5 Liver Lung<br>6 cancer_diagnosis___6 Lymphoma/Hodgkins' Disease Melanoma<br>7 cancer_diagnosis___7 Mouth / Tongue / Lip<br>8 cancer_diagnosis___8 Nervous System<br>9 cancer_diagnosis___9 Ovary (Ovarian)<br>10 cancer_diagnosis___10 Pancreas (Pancreatic)<br>11 cancer_diagnosis___11 Prostate<br>12 cancer_diagnosis___12 Rectum (Rectal)<br>13 cancer_diagnosis___13 Skin (Non-Melanoma)<br>14 cancer_diagnosis___14 Skin (Don't know what kind)<br>15 cancer_diagnosis___15 Soft tissue (muscle or fat)<br>16 cancer_diagnosis___16 Stomach<br>17 cancer_diagnosis___17 Testis (Testicular) Thyroid<br>18 cancer_diagnosis___18 Uterus (Uterine)<br>19 cancer_diagnosis___19 Don't know
121	[bipolar]	Section Header: *Mental Health Diagnosis: Please answer these questions about your mental diagnoses or distresses*<br>Has a doctor or other healthcare provider ever told you that you had bipolar disorder?	yesno, Required<br>1 Yes<br>0 No
122	[schizoaffective]	Has a doctor or other healthcare provider ever told you that you had schizoaffective disorder?	yesno, Required<br>1 Yes<br>0 No
123	[schizophrenia]	Has a doctor or other healthcare provider ever told you that you had schizophrenia?	yesno, Required<br>1 Yes<br>0 No

124	[gad7_nervous]	Section Header: *Over the last two weeks, how often have you been bothered by the following problems?*<br>Feeling nervous, anxious, or on edge	radio (Matrix), Required<br>1 Not at all<br>2 Several days<br>3 More than half the days<br>4 Nearly every day
125	[gad7_worried]	Not being able to stop or control worrying	radio (Matrix), Required<br>1 Not at all<br>2 Several days<br>3 More than half the days<br>4 Nearly every day
126	[gad7_consumed]	Worrying too much about different things	radio (Matrix), Required<br>1 Not at all<br>2 Several days<br>3 More than half the days<br>4 Nearly every day
127	[gad7_troubled]	Trouble relaxing	radio (Matrix), Required<br>1 Not at all<br>2 Several days<br>3 More than half the days<br>4 Nearly every day
128	[gad7_restless]	Being so restless that it is hard to sit still	radio (Matrix), Required<br>1 Not at all<br>2 Several days<br>3 More than half the days<br>4 Nearly every day
129	[gad7_annoyance]	Being easily annoyed or irritable	radio (Matrix), Required<br>1 Not at all<br>2 Several days<br>3 More than half the days<br>4 Nearly every day
130	[gad7_afraid]	Feeling afraid as if something might happen	radio (Matrix), Required<br>1 Not at all<br>2 Several days<br>3 More than half the days<br>4 Nearly every day
131	[cesd_bothered]	Section Header: *Here is a list of ways you might have felt or behaved. Please tell me how often you have felt this way during the past week.*<br>I was bothered by things that usually don't bother me	radio (Matrix), Required<br>1 Rarely or none of the time (Less than 1 day)<br>2 Some or a little of the time (1-2 days)<br>3 Occasionally or a moderate amount of the time (3-4 days)<br>4 Most or all of the time (5-7 days)
132	[cesd_focused]	I had trouble keeping my mind on what I was doing	radio (Matrix), Required<br>1 Rarely or none of the time (Less than 1 day)<br>2 Some or a little of the time (1-2 days)<br>3 Occasionally or a moderate amount of the time (3-4 days)<br>4 Most or all of the time (5-7 days)

133	[cesd_depression]	I felt depressed	radio (Matrix), Required<br>1 Rarely or none of the time (Less than 1 day)<br>2 Some or a little of the time (1-2 days)<br>3 Occasionally or a moderate amount of the time (3-4 days)<br>4 Most or all of the time (5-7 days)
134	[cesd_effort]	I felt that everything I did was an effort	radio (Matrix), Required<br>1 Rarely or none of the time (Less than 1 day)<br>2 Some or a little of the time (1-2 days)<br>3 Occasionally or a moderate amount of the time (3-4 days)<br>4 Most or all of the time (5-7 days)
135	[cesd_hope]	I felt hopeful about the future	radio (Matrix), Required<br>1 Rarely or none of the time (Less than 1 day)<br>2 Some or a little of the time (1-2 days)<br>3 Occasionally or a moderate amount of the time (3-4 days)<br>4 Most or all of the time (5-7 days)
136	[cesd_fear]	I felt fearful	radio (Matrix), Required<br>1 Rarely or none of the time (Less than 1 day)<br>2 Some or a little of the time (1-2 days)<br>3 Occasionally or a moderate amount of the time (3-4 days)<br>4 Most or all of the time (5-7 days)
137	[cesd_restlessness]	My sleep was restless	radio (Matrix), Required<br>1 Rarely or none of the time (Less than 1 day)<br>2 Some or a little of the time (1-2 days)<br>3 Occasionally or a moderate amount of the time (3-4 days)<br>4 Most or all of the time (5-7 days)
138	[cesd_happy]	I was happy	radio (Matrix), Required<br>1 Rarely or none of the time (Less than 1 day)<br>2 Some or a little of the time (1-2 days)<br>3 Occasionally or a moderate amount of the time (3-4 days)<br>4 Most or all of the time (5-7 days)
139	[cesd_lonely]	I felt lonely	radio (Matrix), Required<br>1 Rarely or none of the time (Less than 1 day)<br>2 Some or a little of the time (1-2 days)<br>3 Occasionally or a moderate amount of the time (3-4 days)<br>4 Most or all of the time (5-7 days)
140	[cesd_stagnant]	I could not "get going"	radio (Matrix), Required<br>1 Rarely or none of the time (Less than 1 day)<br>2 Some or a little of the time (1-2 days)<br>3 Occasionally or a moderate amount of the time (3-4 days)<br>4 Most or all of the time (5-7 days)

141	[ptsd_experience]	Section Header: *Sometimes things happen to people that are unusually or especially frightening, horrible, or traumatic. For example: a serious accident or fire, a physical or sexual assault or abuse, an earthquake or flood, a war, seeing someone be killed or seriously injured, having a loved one die through homicide or suicide*<br>Have you ever experienced this kind of event?	radio (Matrix), Required<br>1 Yes<br>2 No
142	[ptsd_nightmares]<br>Show the field ONLY if:<br>[ptsd_experience] = '1'	In the past month, have you had nightmares about the event(s) or thought about the event(s) when you did not want to?	radio (Matrix), Required<br>1 Yes<br>2 No
143	[ptsd_avoidance]<br>Show the field ONLY if:<br>[ptsd_experience] = '1'	In the past month, have you tried hard not to think about the event(s) or went out of your way to avoid situations that reminded you of the event?	radio (Matrix), Required<br>1 Yes<br>2 No
144	[ptsd_startled]<br>Show the field ONLY if:<br>[ptsd_experience] = '1'	In the past month, have you been constantly on guard, watchful, or easily startled?	radio (Matrix), Required<br>1 Yes<br>2 No
145	[ptsd_numb]<br>Show the field ONLY if:<br>[ptsd_experience] = '1'	In the past month, have you felt numb or detached from others, activities, or your surroundings?	radio (Matrix), Required<br>1 Yes<br>2 No
146	[ptsd_guilt]<br>Show the field ONLY if:<br>[ptsd_experience] = '1'	In the past month, have you felt guilty or unable to stop blaming yourself or others for the event(s) or any problems the event(s) may have caused?	radio (Matrix), Required<br>1 Yes<br>2 No
147	[k7_tired]	Section Header: *Kessler Psychological Distress Scale. This 10 item questionnaire is intended to yield a global measure of distress based on questions about anxiety and depressive symptoms that a person has experienced in the last 30 days.*<br>During the last 30 days, about how often did you feel tired out for no good reason?	radio (Matrix), Required<br>1 None of the time<br>2 A little of the time<br>3 Some of the time<br>4 Most of the time<br>5 All of the time
148	[k7_nerves]	During the last 30 days, about how often did you feel nervous?	radio (Matrix), Required<br>1 None of the time<br>2 A little of the time<br>3 Some of the time<br>4 Most of the time<br>5 All of the time
149	[k7_calamity]	During the last 30 days, about how often did you feel so nervous that nothing could calm you down?	radio (Matrix), Required<br>1 None of the time<br>2 A little of the time<br>3 Some of the time<br>4 Most of the time<br>5 All of the time
150	[k7_hopeless]	During the last 30 days, about how often did you feel hopeless?	radio (Matrix), Required<br>1 None of the time<br>2 A little of the time<br>3 Some of the time<br>4 Most of the time<br>5 All of the time

151	[k7_fidgety]	During the last 30 days about how often did you feel restless or fidgety?	radio (Matrix), Required<br>1 None of the time<br>2 A little of the time<br>3 Some of the time<br>4 Most of the time<br>5 All of the time
152	[k7_rest]	During the last 30 days, about how often did you feel so restless you could not sit still?	radio (Matrix), Required<br>1 None of the time<br>2 A little of the time<br>3 Some of the time<br>4 Most of the time<br>5 All of the time
153	[k7_depressive]	During the last 30 days, about how often did you feel depressed?	radio (Matrix), Required<br>1 None of the time<br>2 A little of the time<br>3 Some of the time<br>4 Most of the time<br>5 All of the time
154	[k7_effort]	During the last 30 days, about how often did you feel that everything was an effort?	radio (Matrix), Required<br>1 None of the time<br>2 A little of the time<br>3 Some of the time<br>4 Most of the time<br>5 All of the time
155	[k7_cheer]	During the last 30 days, about how often did you feel so sad that nothing could cheer you up?	radio (Matrix), Required<br>1 None of the time<br>2 A little of the time<br>3 Some of the time<br>4 Most of the time<br>5 All of the time
156	[k7_worth]	During the last 30 days, about how often did you feel worthless?	radio (Matrix), Required<br>1 None of the time<br>2 A little of the time<br>3 Some of the time<br>4 Most of the time<br>5 All of the time
157	[ulss_money]	Section Header: *The Urban Life Stressors Scale is a 21-item instrument to measure community-level stressors as potential sources of psychological and emotional stress experienced by persons living in medium to large cities in 3 domains (economic stability, social & community context, and neighborhood & physical environment). In your day-to-day life, how much stress did you experience from the following:*<br>Money or Finances	radio (Matrix), Required<br>1 no stress<br>2 little stress<br>3 some stress<br>4 a lot of stress<br>5 extreme stress
158	[ulss_job]	Job Satisfaction	radio (Matrix), Required<br>1 no stress<br>2 little stress<br>3 some stress<br>4 a lot of stress<br>5 extreme stress

159	[ulss_parent]	Raising Children/Being a Parent	radio (Matrix), Required<br>1 no stress<br>2 little stress<br>3 some stress<br>4 a lot of stress<br>5 extreme stress
160	[ulss_death]	Death, Injury, or Illness of someone close	radio (Matrix), Required<br>1 no stress<br>2 little stress<br>3 some stress<br>4 a lot of stress<br>5 extreme stress
161	[ulss_housing]	Housing/Living situation	radio (Matrix), Required<br>1 no stress<br>2 little stress<br>3 some stress<br>4 a lot of stress<br>5 extreme stress
162	[ulss_health]	Physical Health	radio (Matrix), Required<br>1 no stress<br>2 little stress<br>3 some stress<br>4 a lot of stress<br>5 extreme stress
163	[ulss_neighborhood]	Neighborhood environment	radio (Matrix), Required<br>1 no stress<br>2 little stress<br>3 some stress<br>4 a lot of stress<br>5 extreme stress
164	[ulss_transit]	Transportation	radio (Matrix), Required<br>1 no stress<br>2 little stress<br>3 some stress<br>4 a lot of stress<br>5 extreme stress
165	[ulss_education]	Education	radio (Matrix), Required<br>1 no stress<br>2 little stress<br>3 some stress<br>4 a lot of stress<br>5 extreme stress
166	[ulss_marriage]	Marriage or Romantic Relationships	radio (Matrix), Required<br>1 no stress<br>2 little stress<br>3 some stress<br>4 a lot of stress<br>5 extreme stress

167	[ulss_famprob]	Other Family Problems	radio (Matrix), Required<br>1 no stress<br>2 little stress<br>3 some stress<br>4 a lot of stress<br>5 extreme stress
168	[ulss_pubsvcs]	Using Public Services	radio (Matrix), Required<br>1 no stress<br>2 little stress<br>3 some stress<br>4 a lot of stress<br>5 extreme stress
169	[ulss_crime]	Crime and Violence	radio (Matrix), Required<br>1 no stress<br>2 little stress<br>3 some stress<br>4 a lot of stress<br>5 extreme stress
170	[ulss_gang]	Gang Activity	radio (Matrix), Required<br>1 no stress<br>2 little stress<br>3 some stress<br>4 a lot of stress<br>5 extreme stress
171	[ulss_racism]	Experiences involving Racism or Discrimination	radio (Matrix), Required<br>1 no stress<br>2 little stress<br>3 some stress<br>4 a lot of stress<br>5 extreme stress
172	[ulss_social]	Social Life or Social Activities	radio (Matrix), Required<br>1 no stress<br>2 little stress<br>3 some stress<br>4 a lot of stress<br>5 extreme stress
173	[ulss_drugs]	Drugs or Alcohol	radio (Matrix), Required<br>1 no stress<br>2 little stress<br>3 some stress<br>4 a lot of stress<br>5 extreme stress
174	[ulss_comm]	Communication or Cultural Conflicts	radio (Matrix), Required<br>1 no stress<br>2 little stress<br>3 some stress<br>4 a lot of stress<br>5 extreme stress

175	[ulss_famviol]	Family Violence	radio (Matrix), Required<br>1 no stress<br>2 little stress<br>3 some stress<br>4 a lot of stress<br>5 extreme stress
176	[ulss_racerelation]	Relations with Racial groups, other than your own	radio (Matrix), Required<br>1 no stress<br>2 little stress<br>3 some stress<br>4 a lot of stress<br>5 extreme stress
177	[ulss_police]	Relations with the police	radio (Matrix), Required<br>1 no stress<br>2 little stress<br>3 some stress<br>4 a lot of stress<br>5 extreme stress
178	[subsistence_needs]	Section Header: *Subsistence needs. The following questions address your ability to afford or access necessities such as food, clothing, and medical care.*<br>In the past year, have you or a family member who you live with been unable to get any of the following when it was really needed? Check all that apply.	checkbox, Required<br>1 subsistence_needs___1 Food<br>2 subsistence_needs___2 Utilities<br>3 subsistence_needs___3 Medicine or any health care (medical, dental, mental health, vision)<br>4 subsistence_needs___4 Phone<br>5 subsistence_needs___5 Clothing<br>6 subsistence_needs___6 Child care<br>7 subsistence_needs___7 Other<br>0 subsistence_needs___0 Does not have any subsistence needs
179	[other_subsistence]<br>Show the field ONLY if:<br>[subsistence_needs(7)] = '1'	If other, please specify	text, Required
180	[maltreatment_treatment]	Section Header: *Maltreatment. (Everyday discrimination scale). In your day to day life, how often have any of the following things happened to you? Please tell me whether you feel that the statement is true almost every day,true at least once a week, true a few times a month, true a few times a year, true less than once a year, or never true for you*<br>You are treated with less courtesy than people.	radio (Matrix)<br>1 Never<br>2 Less than once a year<br>3 A few times a year<br>4 A few times a month<br>5 At least once a week<br>6 Almost everyday<br>7 Don't know or refused
181	[maltreatment_service]	You receive poorer service than other people at restaurants or stores.	radio (Matrix)<br>1 Never<br>2 Less than once a year<br>3 A few times a year<br>4 A few times a month<br>5 At least once a week<br>6 Almost everyday<br>7 Don't know or refused

182	[maltreatment_smart]	People act as if they think you are not smart.	radio (Matrix)<br>1 Never<br>2 Less than once a year<br>3 A few times a year<br>4 A few times a month<br>5 At least once a week<br>6 Almost everyday<br>7 Don't know or refused
183	[maltreatment_perception]	People act as if they are afraid of you.	radio (Matrix)<br>1 Never<br>2 Less than once a year<br>3 A few times a year<br>4 A few times a month<br>5 At least once a week<br>6 Almost everyday<br>7 Don't know or refused
184	[maltreatment_threat]	You are threatened or harassed.	radio (Matrix)<br>1 Never<br>2 Less than once a year<br>3 A few times a year<br>4 A few times a month<br>5 At least once a week<br>6 Almost everyday<br>7 Don't know or refused
185	[inst_shelter]	Section Header: *Institutional Maltreatment*<br>For unfair reasons, do you think that you have ever been denied housing or shelter?	radio (Matrix), Required<br>1 yes<br>0 no<br>2 don't know/refused
186	[inst_work]	For unfair reasons, do you think that you have ever not been hired for a job?	radio (Matrix), Required<br>1 yes<br>0 no<br>2 don't know/refused
187	[inst_harassment]	For unfair reasons, do you think that you have ever been stopped, searched, questioned, physically threatened or abused by the police?	radio (Matrix), Required<br>1 yes<br>0 no<br>2 don't know/refused
188	[covid19_diagnosis]	Section Header: *Covid-19 Screening and Tobacco Use*<br>Have you ever been diagnosed with COVID-19 or tested positive for the coronavirus?	radio, Required<br>1 yes<br>0 no<br>2 don't know/refused
189	[positive_diagnosis]<br>Show the field ONLY if:<br>[covid19_diagnosis] = '1'	If yes, approximate date of diagnosis:	text (date_dmy), Required
190	[hospital_covid]<br>Show the field ONLY if:<br>[covid19_diagnosis] = '1'	If yes, did you stay overnight in the hospital?	yesno, Required<br>1 Yes<br>0 No

191	[covid_ciguse]	How has your cigarette use changed since you learned about the coronavirus pandemic (COVID-19)?	radio, Required<br>1 I increased my cigarette use<br>2 My cigarette use stayed the same<br>3 I decreased my cigarette use<br>4 Other
192	[covidciguse_change]<br>Show the field ONLY if: [covid_ciguse] = '4'	If other, please explain	text, Required
193	[covid_motivation]	How has your motivation to quit smoking changed since you learned about the coronavirus pandemic (COVID-19)?	radio, Required<br>1 My motivation to quit has increased<br>2 My motivation to quit has stayed the same<br>3 My motivation to quit has decreased<br>4 Other
194	[covidciguse_motivation]<br>Show the field ONLY if: [covid_motivation] = '4'	If other, please explain	text, Required
195	[covid_getcovid]	Section Header: *Attitudes and perceptions. On a scale from 1-7, where 1 is "not at all likely" and 7 is "extremely likely*<br>How likely do you think you are to get COVID-19 in the next year?	radio (Matrix), Required<br>1 1 - Not at all<br>2 2<br>3 3<br>4 4<br>5 5<br>6 6<br>7 7 - Extremely
196	[covid_concernhealth]	How concerned about coronavirus (COVID-19) are you for the health of others in your life?	radio (Matrix), Required<br>1 1 - Not at all<br>2 2<br>3 3<br>4 4<br>5 5<br>6 6<br>7 7 - Extremely
197	[covid_cigharm]	How much do you think your cigarette use increases your risk of harm from coronavirus (COVID-19)?	radio (Matrix), Required<br>1 1 - Not at all<br>2 2<br>3 3<br>4 4<br>5 5<br>6 6<br>7 7 - Extremely
198	[covid_housing]	Section Header: *Many things have changed during the COVID-19/coronavirus pandemic. What are some of the changes you have experienced?*<br>I moved from the street to a shelter	radio (Matrix), Required<br>1 yes<br>0 no<br>2 don't know/refused
199	[covid_waitlisthotel]	I am on the waitlist for a hotel/motel	radio (Matrix), Required<br>1 yes<br>0 no<br>2 don't know/refused

200	[covid_increaseinc]	My monthly income went up	radio (Matrix), Required<br>1 yes<br>0 no<br>2 don't know/refused
201	[covid_decreaseinc]	My monthly income went down	radio (Matrix), Required<br>1 yes<br>0 no<br>2 don't know/refused
202	[covid_dx]	I was diagnosed with COVID-19	radio (Matrix), Required<br>1 yes<br>0 no<br>2 don't know/refused
203	[covid_illnessothers]	Someone I know got sick from COVID-19	radio (Matrix), Required<br>1 yes<br>0 no<br>2 don't know/refused
204	[covid_deathothers]	Someone I know died from COVID-19	radio (Matrix), Required<br>1 yes<br>0 no<br>2 don't know/refused
205	[covid_vaccination]	I was vaccinated for COVID-19	radio (Matrix), Required<br>1 yes<br>0 no<br>2 don't know/refused
206	[gender]	Section Header: *Demographics*<br>What terms best expresses how you describe your gender identity?	radio, Required<br>1 Female<br>2 Male<br>3 Non-Binary<br>4 Transgender<br>5 None of these describe me and I'd like to consider additional options<br>6 Prefer not to answer
207	[gender_cat]<br>Show the field ONLY if: [gender] = '5'	Are any of these a closer description to your gender identity?	radio<br>1 Trans man/Transgender Man/FTM<br>2 Trans woman/Transgender/MTF<br>3 Genderqueer<br>4 Genderfluid<br>5 Gender variant<br>6 Questioning or unsure of your gender identity<br>7 None of these describe me and I want to specify
208	[gender_specificity]<br>Show the field ONLY if: [gender_cat] = '7'	Please specify your gender identification	text
209	[ethnicity]	Ethnicity	radio, Required<br>0 Hispanic or Latino<br>1 NOT Hispanic or Latino<br>2 Unknown / Not Reported

210	[race]	Race	radio, Required<br>0 American Indian/Alaska Native<br>1 Asian<br>2 Native Hawaiian or Other Pacific Islander<br>3 Black or African American<br>4 White<br>5 More Than One Race<br>6 Unknown / Not Reported
211	[education]	Section Header: *Education*<br>What is your highest level of education?	radio<br>1 Less than high school<br>2 High school or GED<br>3 Some college<br>4 College or professional training
212	[income]	What is your current MONTHLY household income? Please include salary, SSI, Disability, GA, other public benefits, pensions, interests, etc. for both you and your partner (if applicable	text (number), Required
213	[employment]	Section Header: *Employment Status*<br>Are you currently employed?	yesno, Required<br>1 Yes<br>0 No
214	[recent_lodging]	Section Header: *Housing*<br>Please share where you slept last night?	radio, Required<br>1 In an emergency shelter or safe haven<br>2 In transitional housing<br>3 In an institution (including hospital, jail, prison, juvenile detention facility, long-term care facility, or nursing home)?<br>4 In a place not meant for human habitation (including in a car, unsheltered on the street or under a bridge, etc.)<br>5 In housing you shared with others, but did not own or rent<br>6 In housing that you rented<br>7 In a hotel or motel<br>8 In a short term SRO<br>9 In a shelter in place hotel<br>10 In a permanent supportive housing
215	[lengthofstay_years]	How long have you stayed in the place you stayed at last night (years)?	text (number), Required
216	[lenghtofstay_days]	How long have you stayed in the place you stayed at last night (in days)?	text (number), Required
217	[homeless_continuously]	Have you been continuously homeless in the past 12 months?	radio, Required<br>1 yes<br>0 no<br>2 don't know/refused
218	[episodic_homelessness]	How many episodes of homelessness have you had in the past 3 years?	text (number, Min: 1), Required
219	[rct_participant_survey_baseline_complete]	Section Header: *Form Status*<br>Complete?	dropdown<br>0 Incomplete<br>1 Unverified<br>2 Complete

Instrument: **Randomization** (randomization)

220	[study_idrandom]	Study ID	text, Required
221	[random_confirm]	Exposure: [screen_firstcig]<br>Recruiter: [recruiter]	descriptive
222	[random_grp]	Treatment Group	radio, Required<br>1 Group A<br>2 Group B
223	[random_secondid]	[initial_data_arm_1][participant_id]-[initial_data_arm_1][random_grp]	descriptive
224	[randomization_complete]	Section Header: *Form Status*<br>Complete?	dropdown<br>0 Incomplete<br>1 Unverified<br>2 Complete
Instrument: **Rct Participant Survey Followup** (rct_participant_survey_followup)			
225	[study_idfollowup]	Study ID	text, Required
226	[screen_pregnancy_v2]	Section Header: *Screening Questions*<br>Are you currently pregnant?	radio, Required<br>1 Yes<br>2 No
227	[screen_heartattack_v2]	Have you had a myocardial infarction (heart attack) in the past two weeks?	radio, Required<br>1 yes<br>2 no
228	[current_date_v2]	Section Header: *Please fill out the information below.*<br>Current Date	text (date_mdy), Required
229	[first_name_v2]	First Name	text, Required
230	[last_name_v2]	Last Name	text, Required
231	[dob_v2]	Date of birth	text (date_mdy)
232	[email_v2]	E-mail address	text (email), Identifier
233	[phone_number_v2]	Phone Number	text, Required
234	[medical_care_loc_v2]	Location of Medical Care	text, Required
235	[pcp_v2]	Name of Primary Care Provider	text, Required
236	[lodging_v2]	Where did you sleep last night?	text, Required
237	[consent_v2]	By checking this box, I certify that I am at least 18 years old and that I give my consent freely to participant in this study.	checkbox<br>1 consent_v2___1 I consent
238	[co_reading_v2]	Carbon Monoxide Reading	text (number), Required
239	[smoker_frequency_v2]	Section Header: *Cigarette Use and Smoking History*<br>How often do you smoke?	radio, Required<br>1 Everyday<br>2 Somedays<br>3 Not at all
240	[menthol_v2]<br>Show the field ONLY if:<br>[smoker_frequency_v2] = '1' or [smoker_frequency_v2] = '2'	Do you usually smoke menthols or non-menthols?	radio<br>1 Menthols<br>2 Non-Menthols
241	[timetofirstcig_v2]<br>Show the field ONLY if:<br>[smoker_frequency_v2] = '1' or [smoker_frequency_v2] = '2'	If there were no restrictions on you, how soon after you woke up would you have your first cigarette?	radio, Required<br>1 Within 5 minutes<br>2 6-30 minutes<br>3 31-60 minutes<br>4 After 60 minutes

242	[cessation_intention_v2]<br><br>Show the field ONLY if:<br>[smoker_frequency_v2] = '1' o<br>r [smoker_frequency_v2] = '2'	What best describes your intention to quit smoking?	radio<br>1 I never expect to quit<br>2 I may quit<br>3 I will quit in the next 6 months<br>4 I will quit in the next month
243	[quit_attempts_v2]	Since the last visit, have you stopped smoking for one day or longer because you were trying to stop smoking?	yesno, Required<br>1 Yes<br>0 No
244	[lengthofquit_v2]<br><br>Show the field ONLY if:<br>[quit_attempts_v2] = '1'	How long were you able to go without smoking during your last quit attempt (number of days) in the past 12 months?	text (number, Min: 1, Max: 365), Required
245	[cessationaids_v2]	What products, methods or resources did you use to help you stop smoking in the last quit attempt? (check all that apply, or "never tried to quit")?	checkbox, Required<br>1 cessationaids_v2___1 Quit "cold turkey"<br>2 cessationaids_v2___2 Gradually cut down<br>3 cessationaids_v2___3 Smoking cessation class<br>4 cessationaids_v2___4 Advice from a healthcare professional (doctor, nurse, psychologist, etc.)<br>5 cessationaids_v2___5 Advice from shelter staff<br>6 cessationaids_v2___6 Called a telephone hotline<br>7 cessationaids_v2___7 Hypnosis<br>8 cessationaids_v2___8 Acupuncture<br>9 cessationaids_v2___9 Nicotine gum<br>10 cessationaids_v2___10 Nicotine patch<br>11 cessationaids_v2___11 Nicotine spray<br>12 cessationaids_v2___12 Nicotine lozenge<br>13 cessationaids_v2___13 Nicotine inhaler<br>14 cessationaids_v2___14 Zyban/Wellbutrin for smoking cessation<br>15 cessationaids_v2___15 Chantix/Varenicline for smoking cessation<br>16 cessationaids_v2___16 E-cigarettes to help cut down or stop using regular cigarettes<br>17 cessationaids_v2___17 Smokeless tobacco<br>18 cessationaids_v2___18 Other<br>19 cessationaids_v2___19 Never tried to quit
246	[other_nrt_v2]<br><br>Show the field ONLY if:<br>[cessationaids_v2(18)] = '1'	If other, please explain	text, Required
247	[past7dayssmk_v2]	Did you smoke any cigarettes in the past 7 days?	yesno, Required<br>1 Yes<br>0 No
248	[noofdayssmk_past7_v2]<br><br>Show the field ONLY if:<br>[past7dayssmk_v2] = '1'	Out of the past 7 days, on how many days did you smoke cigarettes?	text (number, Min: 1, Max: 7), Required

249	[noofcigssmk_v2]<br>Show the field ONLY if:<br>[past7dayssmk_v2] = '1'	In the past 7 days, on the days that you did smoke, about how many cigarettes did you usually smoke per day?	text (number, Min: 1), Required
250	[ecig_past30_v2]	Section Header: *I'm going to ask you about products you may or may not have used. Please answer "yes" if you have ever tried the product, even just once in your life.*<br>Have you used an e-cigarette in the past 30 days?	yesno<br>1 Yes<br>0 No
251	[ecig_nodays_v2]<br>Show the field ONLY if:<br>[ecig_past30_v2] = '1'	In the past 30 days, how many days did you use an e-cigarette?	text (number, Min: 1, Max: 30), Required
252	[ecig_timeofuse_v2]<br>Show the field ONLY if:<br>[ecig_past30_v2] = '1'	How many times per day do you usually use your e-cigarette / vape pen (assume that one "time" consists of around 15 puffs or lasts around 10 minutes)?	text (number, Min: 1), Required
253	[ecig_nicotine_v2]<br>Show the field ONLY if:<br>[ecig_past30_v2] = '1'	What concentration of nicotine do you usually use in your e-cigarette?	radio, Required<br>1 1-6 mg<br>2 7-12 mg<br>3 13-18 mg<br>4 19-24 mg<br>5 25< + mg
254	[ecig_patternuse_v2]<br>Show the field ONLY if:<br>[ecig_past30_v2] = '1'	How do you usually smoke your e-cigarette / vapor pen?	radio, Required<br>1 Continuously throughout the day (only a few puffs<br>2 each time)<br>3 Distinct smoking bouts that are shorter than smoking a traditional cigarette (shorter length of time and fewer number of puffs)<br>4 Distinct smoking bouts similar to smoking a traditional cigarette (similar length of time and number of puffs)<br>5 Other
255	[other_eciguse_v2]<br>Show the field ONLY if:<br>[ecig_patternuse_v2] = '5'	If other, please explain	text
256	[ecig_timeusage_v2]<br>Show the field ONLY if:<br>[ecig_past30_v2] = '1'	During the past 30 days, on the days when you used e-cigarettes, about how much time did you use each day?	radio, Required<br>1 Just a few puffs, less than 1 minute total<br>2 1-5 minutes total<br>3 6-15 minutes total<br>4 16-60 minutes total<br>5 More than 1 hour total use

257	[ecig_motivation_v2]<br><br>Show the field ONLY if:<br>[ecig_past30_v2] = '1'	What best describes your motivation to use e-cigarettes?	checkbox<br>1 ecig_motivation_v2___1 E-cigarettes help me to quit smoking<br>2 ecig_motivation_v2___2 E-cigarettes help me to cut down on smoking<br>3 ecig_motivation_v2___3 E-cigarettes are cheaper than smoking cigarettes<br>4 ecig_motivation_v2___4 E-cigarettes are less harmful to my health than cigarettes<br>5 ecig_motivation_v2___5 E-cigarettes can be used in places where cigarettes are not allowed<br>6 ecig_motivation_v2___6 E-cigarettes taste better and are more pleasurable to use than cigarettes<br>7 ecig_motivation_v2___7 I use this product for reasons not listed<br>8 ecig_motivation_v2___8 I am trying to stop smoking cigarettes<br>9 ecig_motivation_v2___9 Other<br>10 ecig_motivation_v2___10 None of the above
258	[other_ecigmotivation_v2]<br><br>Show the field ONLY if:<br>[ecig_motivation_v2(9)] = '1'	If other, please explain	text, Required
259	[smkless_past30_v2]	Have you used smokeless tobacco in the past 30 days?	yesno, Required<br>1 Yes<br>0 No
260	[smkless_noofdays_v2]<br><br>Show the field ONLY if:<br>[smkless_past30_v2] = '1'	In the past 30 days, how many days did you use smokeless tobacco?	text (number, Min: 1, Max: 30), Required
261	[cigar_past30_v2]	Have you used cigars, cigarillos, or little cigars in the past 30 days	yesno, Required<br>1 Yes<br>0 No
262	[cigar_nodays_v2]<br><br>Show the field ONLY if:<br>[cigar_past30_v2] = '1'	In the last 30 days, on about how many days did you smoke cigars, cigarillos or little cigars?	text (number, Min: 1, Max: 30), Required
263	[pipe_past30_v2]	Have you used a tobacco pipe in the past 30 days?	yesno, Required<br>1 Yes<br>0 No
264	[pipe_nodays_v2]<br><br>Show the field ONLY if:<br>[pipe_past30_v2] = '1'	In the last 30 days, on about how many days did you smoke a tobacco pipe?	text (number, Min: 1, Max: 30), Required
265	[hookah_past30_v2]	Have you used a hookah in the past 30 days?	yesno, Required<br>1 Yes<br>0 No
266	[hookah_nodays_v2]<br><br>Show the field ONLY if:<br>[hookah_past30_v2] = '1'	In the last 30 days, on about how many days did you smoke from a hookah?	text (number, Min: 1, Max: 30), Required

267	[marijuana_past30_v2]	Have you used marijuana (cannabis) or hashish in the past 30 days?	yesno, Required<br>1 Yes<br>0 No
268	[marijuana_nodays_v2]<br>Show the field ONLY if:<br>[marijuana_past30_v2] = '1'	In the last 30 days, on about how many days did you use marijuana (cannabis) or hashish?	text (number, Min: 1, Max: 30), Required
269	[blunt_past30days]	Have you used a blunt (marijuana rolled in a tobacco leaf), in the past 30 days?	yesno, Required<br>1 Yes<br>0 No
270	[blunts_30daysf]<br>Show the field ONLY if:<br>[blunt_past30days] = '1'	In the last 30 days, on about how many days did you use blunts?	text (number, Min: 1, Max: 30), Required
271	[cannabismotivations_v2]<br>Show the field ONLY if:<br>[marijuana_past30_v2] = '1'	What are some of the reasons for your using marijuana (cannabis) or hashish? (Mark all that apply.)	checkbox, Required<br>1 cannabismotivations_v2___1 To experiment - to see what it's like<br>2 cannabismotivations_v2___2 To relax or relieve tension<br>3 cannabismotivations_v2___3 To feel good or get high<br>4 cannabismotivations_v2___4 To seek deeper insights and understanding<br>5 cannabismotivations_v2___5 To have a good time with my friends<br>6 cannabismotivations_v2___6 To fit in with a group I like<br>7 cannabismotivations_v2___7 To get away from my problems or troubles<br>8 cannabismotivations_v2___8 To relieve of boredom, nothing else to do<br>9 cannabismotivations_v2___9 To release of anger or frustration<br>10 cannabismotivations_v2___10 To get through the day<br>11 cannabismotivations_v2___11 To increase the effects of some other drug(s)<br>12 cannabismotivations_v2___12 To decrease (offset) the effects of some other drug(s)<br>13 cannabismotivations_v2___13 To get to sleep<br>14 cannabismotivations_v2___14 It tastes good<br>15 cannabismotivations_v2___15 I am "hooked"- I feel I have to have it<br>16 cannabismotivations_v2___16 To quit or cut down on smoking cigarettes<br>17 cannabismotivations_v2___17 Other

272	[other_cannamotiv_v2]<br>Show the field ONLY if:<br>[cannabismotivations_v2(17)] = '1'	If other, please explain	text
273	[medmarijuana_card_v2]<br>Show the field ONLY if:<br>[marijuana_past30_v2] = '1'	Do you currently have a medical marijuana card?	yesno, Required<br>1 Yes<br>0 No
274	[tobacco_sources_v2]<br>Show the field ONLY if:<br>[smoker_frequency_v2] = '1' or [smoker_frequency_v2] = '2'	Where do you usually get your tobacco products? Please tell us your most common sources	checkbox<br>1 tobacco_sources_v2___1 From a liquor store, convenience store or gas station<br>2 tobacco_sources_v2___2 From a supermarket or drug store<br>3 tobacco_sources_v2___3 From a smoke shop, vape shop or marijuana dispensary<br>4 tobacco_sources_v2___4 From friends or strangers (Please check all that apply.)
275	[easy_purchase_v2]<br>Show the field ONLY if:<br>[smoker_frequency_v2] = '1' or [smoker_frequency_v2] = '2'	How easy was it to get your typical tobacco products in the past 30 days?	radio<br>1 Very difficult<br>2 Somewhat difficult<br>3 Somewhat easy<br>4 Very easy<br>5 Not Applicable
276	[audit7_freq_v2]	Section Header: *Alcohol Use. Please answer the following questions regarding alcohol use.*<br>How often do you have a drink containing alcohol?	radio, Required<br>1 Never<br>2 Monthly or less<br>3 2-4 times a month<br>4 2-3 times a week<br>5 4 or more times a week
277	[audit7alcohol_quantity_v2]<br>Show the field ONLY if:<br>[audit7_freq_v2] = '2' or [audit7_freq_v2] = '3' or [audit7_freq_v2] = '4' or [audit7_freq_v2] = '5'	How many standard drinks containing alcohol do you have on a typical day?	radio, Required<br>0 0<br>1 1 to 2<br>2 3 to 4<br>3 5 to 6<br>4 7 to 9<br>5 10 or more
278	[audit7alcohol_intake_v2]<br>Show the field ONLY if:<br>[audit7_freq_v2] = '2' or [audit7_freq_v2] = '3' or [audit7_freq_v2] = '4' or [audit7_freq_v2] = '5'	How often do you have six or more drinks on one occasion	radio, Required<br>1 Never<br>2 Less than monthly<br>3 Monthly<br>4 Weekly<br>5 Daily or almost daily
279	[audit7gender_intake_v2]<br>Show the field ONLY if:<br>[audit7_freq_v2] = '2' or [audit7_freq_v2] = '3' or [audit7_freq_v2] = '4' or [audit7_freq_v2] = '5'	During the past 30 days, on how many days did you drink at least 4 or more alcoholic drinks (if you are a woman) OR 5 or more alcoholic drinks (if you are a man)?	radio, Required<br>1 Never<br>2 1-3 times this month<br>3 1-2 times a week<br>4 3 or more times a week

280	[cocaine_past30_v2]	Section Header: *This next section asks about your use of drugs. I am going to ask you some questions about your experience of using different substances across your lifetime and in the past 6 months. These substances can be smoked, swallowed, snorted, inhaled, injected or taken in the form of pills. Some substances listed may be prescribed by a doctor (like amphetamines, sedatives, pain medications). For this interview, we will NOT record medications that are used AS PRESCRIBED by your doctor. However, if you have taken such medications for reasons other than prescription, or taken them more frequently or at higher doses than prescribed, please let me know. While we are also interested in knowing about your use of various illicit drugs, please be assured that information on such use will be treated strictly confidential.*<br>Have you used cocaine in the past 30 days?	yesno, Required<br>1 Yes<br>0 No
281	[cocaine_nodays_v2]<br>Show the field ONLY if:<br>[cocaine_past30_v2] = '1'	In the past 30 days, how many days did you use Cocaine, or Crack?	text (number, Min: 1, Max: 30), Required
282	[amphetamines_past30_v2]	Have you used amphetamines in the past 30 days?	yesno, Required<br>1 Yes<br>0 No
283	[amphetamines_nodays_v2]<br>Show the field ONLY if:<br>[amphetamines_past30_v2] = '1'	In the last 30 days, how many days have you used amphetamines?	text (number, Min: 1, Max: 30), Required
284	[inhalants_past30_v2]	Have you used any inhalants in the last 30 days?	yesno, Required<br>1 Yes<br>0 No
285	[inhalants_nodays_v2]<br>Show the field ONLY if:<br>[inhalants_past30_v2] = '1'	In the last 30 days, how many days did you use inhalants?	text (number, Min: 1, Max: 30), Required
286	[sedative_past30_v2]	Have you used any sedatives or sleeping pills in the last 30 days?	yesno, Required<br>1 Yes<br>0 No
287	[sedatives_nodays_v2]<br>Show the field ONLY if:<br>[sedative_past30_v2] = '1'	In the last 30 days, how many days have you used sleeping pills or sedatives?	text (number, Min: 1, Max: 30), Required
288	[hallucinogens_past30_v2]	Have you used any hallucinogens in the last 30 days?	yesno, Required<br>1 Yes<br>0 No
289	[hallucinogens_nodays_v2]<br>Show the field ONLY if:<br>[hallucinogens_past30_v2] = '1'	In the last 30 days, how many days did you use hallucinogens?	text (number, Min: 1, Max: 30), Required
290	[opiods_past30_v2]	Have you used any opioids in the last 30 days?	yesno, Required<br>1 Yes<br>0 No
291	[opioids_nodays_v2]<br>Show the field ONLY if:<br>[opiods_past30_v2] = '1'	In the last 30 days, how many days did you use opioids?	text (number, Min: 1, Max: 30), Required
292	[otherdrugs_past30_v2]	Have you used any other drugs in the last 30 days?	yesno, Required<br>1 Yes<br>0 No
293	[otherdrugs_nodays_v2]<br>Show the field ONLY if:<br>[otherdrugs_past30_v2] = '1'	In the last 30 days, how many days did you use any other drugs?	text (number, Min: 1, Max: 30), Required

294	[gad7_nervous_v2]	Section Header: *Over the last two weeks, how often have you been bothered by the following problems?*<br>Feeling nervous, anxious, or on edge	radio (Matrix), Required<br>1 Not at all<br>2 Several days<br>3 More than half the days<br>4 Nearly every day
295	[gad7_worried_v2]	Not being able to stop or control worrying	radio (Matrix), Required<br>1 Not at all<br>2 Several days<br>3 More than half the days<br>4 Nearly every day
296	[gad7_consumed_v2]	Worrying too much about different things	radio (Matrix), Required<br>1 Not at all<br>2 Several days<br>3 More than half the days<br>4 Nearly every day
297	[gad7_troubled_v2]	Trouble relaxing	radio (Matrix), Required<br>1 Not at all<br>2 Several days<br>3 More than half the days<br>4 Nearly every day
298	[gad7_restless_v2]	Being so restless that it is hard to sit still	radio (Matrix), Required<br>1 Not at all<br>2 Several days<br>3 More than half the days<br>4 Nearly every day
299	[gad7_annoyance_v2]	Being easily annoyed or irritable	radio (Matrix), Required<br>1 Not at all<br>2 Several days<br>3 More than half the days<br>4 Nearly every day
300	[gad7_afraid_v2]	Feeling afraid as if something might happen	radio (Matrix), Required<br>1 Not at all<br>2 Several days<br>3 More than half the days<br>4 Nearly every day
301	[cesd_bothered_v2]	Section Header: *Here is a list of ways you might have felt or behaved. Please tell me how often you have felt this way during the past week.*<br>I was bothered by things that usually don't bother me	radio (Matrix), Required<br>1 Rarely or none of the time (Less than 1 day)<br>2 Some or a little of the time (1-2 days)<br>3 Occasionally or a moderate amount of the time (3-4 days)<br>4 Most or all of the time (5-7 days)
302	[cesd_focused_v2]	I had trouble keeping my mind on what I was doing	radio (Matrix), Required<br>1 Rarely or none of the time (Less than 1 day)<br>2 Some or a little of the time (1-2 days)<br>3 Occasionally or a moderate amount of the time (3-4 days)<br>4 Most or all of the time (5-7 days)

303	[cesd_depression_v2]	I felt depressed	radio (Matrix), Required<br>1 Rarely or none of the time (Less than 1 day)<br>2 Some or a little of the time (1-2 days)<br>3 Occasionally or a moderate amount of the time (3-4 days)<br>4 Most or all of the time (5-7 days)
304	[cesd_effort_v2]	I felt that everything I did was an effort	radio (Matrix), Required<br>1 Rarely or none of the time (Less than 1 day)<br>2 Some or a little of the time (1-2 days)<br>3 Occasionally or a moderate amount of the time (3-4 days)<br>4 Most or all of the time (5-7 days)
305	[cesd_hope_v2]	I felt hopeful about the future	radio (Matrix), Required<br>1 Rarely or none of the time (Less than 1 day)<br>2 Some or a little of the time (1-2 days)<br>3 Occasionally or a moderate amount of the time (3-4 days)<br>4 Most or all of the time (5-7 days)
306	[cesd_fear_v2]	I felt fearful	radio (Matrix), Required<br>1 Rarely or none of the time (Less than 1 day)<br>2 Some or a little of the time (1-2 days)<br>3 Occasionally or a moderate amount of the time (3-4 days)<br>4 Most or all of the time (5-7 days)
307	[cesd_restlessness_v2]	My sleep was restless	radio (Matrix), Required<br>1 Rarely or none of the time (Less than 1 day)<br>2 Some or a little of the time (1-2 days)<br>3 Occasionally or a moderate amount of the time (3-4 days)<br>4 Most or all of the time (5-7 days)
308	[cesd_happy_v2]	I was happy	radio (Matrix), Required<br>1 Rarely or none of the time (Less than 1 day)<br>2 Some or a little of the time (1-2 days)<br>3 Occasionally or a moderate amount of the time (3-4 days)<br>4 Most or all of the time (5-7 days)
309	[cesd_lonely_v2]	I felt lonely	radio (Matrix), Required<br>1 Rarely or none of the time (Less than 1 day)<br>2 Some or a little of the time (1-2 days)<br>3 Occasionally or a moderate amount of the time (3-4 days)<br>4 Most or all of the time (5-7 days)
310	[cesd_stagnant_v2]	I could not "get going"	radio (Matrix), Required<br>1 Rarely or none of the time (Less than 1 day)<br>2 Some or a little of the time (1-2 days)<br>3 Occasionally or a moderate amount of the time (3-4 days)<br>4 Most or all of the time (5-7 days)

311	[ptsd_experience_v2]	Section Header: *Sometimes things happen to people that are unusually or especially frightening, horrible, or traumatic. For example: a serious accident or fire, a physical or sexual assault or abuse, an earthquake or flood, a war, seeing someone be killed or seriously injured, having a loved one die through homicide or suicide.*<br>Have you ever experienced this kind of event?	radio (Matrix), Required<br>1 Yes<br>2 No
312	[ptsd_nightmares_v2]<br>Show the field ONLY if:<br>[ptsd_experience_v2]='1'	In the past month, had nightmares about the event(s) or thought about the event(s) when you did not want to?	radio (Matrix), Required<br>1 Yes<br>2 No
313	[ptsd_avoidance_v2]<br>Show the field ONLY if:<br>[ptsd_experience_v2]='1'	In the past month, tried hard not to think about the event(s) or went out of your way to avoid situations that reminded you of the event(s)?	radio (Matrix), Required<br>1 Yes<br>2 No
314	[ptsd_startled_v2]<br>Show the field ONLY if:<br>[ptsd_experience_v2]='1'	In the past month, been constantly on guard, watchful, or easily startled?	radio (Matrix), Required<br>1 Yes<br>2 No
315	[ptsd_numb_v2]<br>Show the field ONLY if:<br>[ptsd_experience_v2]='1'	In the past month, felt numb or detached from others, activities, or your surroundings?	radio (Matrix), Required<br>1 Yes<br>2 No
316	[ptsd_guilt_v2]<br>Show the field ONLY if:<br>[ptsd_experience_v2]='1'	In the past month, felt guilty or unable to stop blaming yourself or others for the event(s) or any problems the event(s) may have caused?	radio (Matrix), Required<br>1 Yes<br>2 No
317	[k7_tired_v2]	Section Header: *Kessler Psychological Distress Scale. This 10 item questionnaire is intended to yield a global measure of distress based on questions about anxiety and depressive symptoms that a person has experienced in the last 30 days.*<br>During the last 30 days, about how often did you feel tired out for no good reason?	radio (Matrix), Required<br>1 None of the time<br>2 A little of the time<br>3 Some of the time<br>4 Most of the time<br>5 All of the time
318	[k7_nerves_v2]	During the last 30 days, about how often did you feel nervous?	radio (Matrix), Required<br>1 None of the time<br>2 A little of the time<br>3 Some of the time<br>4 Most of the time<br>5 All of the time
319	[k7_calamity_v2]	During the last 30 days, about how often did you feel so nervous that nothing could calm you down?	radio (Matrix), Required<br>1 None of the time<br>2 A little of the time<br>3 Some of the time<br>4 Most of the time<br>5 All of the time
320	[k7_hopeless_v2]	During the last 30 days, about how often did you feel hopeless?	radio (Matrix), Required<br>1 None of the time<br>2 A little of the time<br>3 Some of the time<br>4 Most of the time<br>5 All of the time

321	[k7_fidgety_v2]	During the last 30 days about how often did you feel restless or fidgety?	radio (Matrix), Required<br>1 None of the time<br>2 A little of the time<br>3 Some of the time<br>4 Most of the time<br>5 All of the time
322	[k7_rest_v2]	During the last 30 days, about how often did you feel so restless you could not sit still?	radio (Matrix), Required<br>1 None of the time<br>2 A little of the time<br>3 Some of the time<br>4 Most of the time<br>5 All of the time
323	[k7_depressive_v2]	During the last 30 days, about how often did you feel depressed?	radio (Matrix), Required<br>1 None of the time<br>2 A little of the time<br>3 Some of the time<br>4 Most of the time<br>5 All of the time
324	[k7_effort_v2]	During the last 30 days, about how often did you feel that everything was an effort?	radio (Matrix), Required<br>1 None of the time<br>2 A little of the time<br>3 Some of the time<br>4 Most of the time<br>5 All of the time
325	[k7_cheer_v2]	During the last 30 days, about how often did you feel so sad that nothing could cheer you up?	radio (Matrix), Required<br>1 None of the time<br>2 A little of the time<br>3 Some of the time<br>4 Most of the time<br>5 All of the time
326	[k7_worth_v2]	During the last 30 days, about how often did you feel worthless?	radio (Matrix), Required<br>1 None of the time<br>2 A little of the time<br>3 Some of the time<br>4 Most of the time<br>5 All of the time
327	[ulss_money_v2]	Section Header: *The Urban Life Stressors Scale is a 21-item instrument to measure community-level stressors as potential sources of psychological and emotional stress experienced by persons living in medium to large cities in 3 domains (economic stability, social & community context, and neighborhood & physical environment). In your day-to-day life, how much stress did you experience from the following:*<br>Money or Finances	radio (Matrix), Required<br>1 no stress<br>2 little stress<br>3 some stress<br>4 a lot of stress<br>5 extreme stress
328	[ulss_job_v2]	Job Satisfaction	radio (Matrix), Required<br>1 no stress<br>2 little stress<br>3 some stress<br>4 a lot of stress<br>5 extreme stress

329	[ulss_parent_v2]	Raising Children/Being a Parent	radio (Matrix), Required<br>1 no stress<br>2 little stress<br>3 some stress<br>4 a lot of stress<br>5 extreme stress
330	[ulss_death_v2]	Death, Injury, or Illness of someone close	radio (Matrix), Required<br>1 no stress<br>2 little stress<br>3 some stress<br>4 a lot of stress<br>5 extreme stress
331	[ulss_housing_v2]	Housing/Living situation	radio (Matrix), Required<br>1 no stress<br>2 little stress<br>3 some stress<br>4 a lot of stress<br>5 extreme stress
332	[ulss_health_v2]	Physical Health	radio (Matrix), Required<br>1 no stress<br>2 little stress<br>3 some stress<br>4 a lot of stress<br>5 extreme stress
333	[ulss_neighborhood_v2]	Neighborhood environment	radio (Matrix), Required<br>1 no stress<br>2 little stress<br>3 some stress<br>4 a lot of stress<br>5 extreme stress
334	[ulss_transit_v2]	Transportation	radio (Matrix), Required<br>1 no stress<br>2 little stress<br>3 some stress<br>4 a lot of stress<br>5 extreme stress
335	[ulss_education_v2]	Education	radio (Matrix), Required<br>1 no stress<br>2 little stress<br>3 some stress<br>4 a lot of stress<br>5 extreme stress
336	[ulss_marriage_v2]	Marriage or Romantic Relationships	radio (Matrix), Required<br>1 no stress<br>2 little stress<br>3 some stress<br>4 a lot of stress<br>5 extreme stress

337	[ulss_famprob_v2]	Other Family Problems	radio (Matrix), Required<br>1 no stress<br>2 little stress<br>3 some stress<br>4 a lot of stress<br>5 extreme stress
338	[ulss_pubsvcs_v2]	Using Public Services	radio (Matrix), Required<br>1 no stress<br>2 little stress<br>3 some stress<br>4 a lot of stress<br>5 extreme stress
339	[ulss_crime_v2]	Crime and Violence	radio (Matrix), Required<br>1 no stress<br>2 little stress<br>3 some stress<br>4 a lot of stress<br>5 extreme stress
340	[ulss_gang_v2]	Gang Activity	radio (Matrix), Required<br>1 no stress<br>2 little stress<br>3 some stress<br>4 a lot of stress<br>5 extreme stress
341	[ulss_racism_v2]	Experiences involving Racism or Discrimination	radio (Matrix), Required<br>1 no stress<br>2 little stress<br>3 some stress<br>4 a lot of stress<br>5 extreme stress
342	[ulss_social_v2]	Social Life or Social Activities	radio (Matrix), Required<br>1 no stress<br>2 little stress<br>3 some stress<br>4 a lot of stress<br>5 extreme stress
343	[ulss_drugs_v2]	Drugs or Alcohol	radio (Matrix), Required<br>1 no stress<br>2 little stress<br>3 some stress<br>4 a lot of stress<br>5 extreme stress
344	[ulss_comm_v2]	Communication or Cultural Conflicts	radio (Matrix), Required<br>1 no stress<br>2 little stress<br>3 some stress<br>4 a lot of stress<br>5 extreme stress

345	[ulss_famviol_v2]	Family Violence	radio (Matrix), Required<br>1 no stress<br>2 little stress<br>3 some stress<br>4 a lot of stress<br>5 extreme stress
346	[ulss_racerelation_v2]	Relations with Racial groups, other than your own	radio (Matrix), Required<br>1 no stress<br>2 little stress<br>3 some stress<br>4 a lot of stress<br>5 extreme stress
347	[ulss_police_v2]	Relations with the police	radio (Matrix), Required<br>1 no stress<br>2 little stress<br>3 some stress<br>4 a lot of stress<br>5 extreme stress
348	[subsistence_needs_v2]	Section Header: *Subsistence needs. The following questions address your ability to afford or access necessities such as food, clothing, and medical care.*<br>Since the last visit, have you or a family member who you live with been unable to get any of the following when it was really needed? Check all that apply.	checkbox, Required<br>1 subsistence_needs_v2___1 Food<br>2 subsistence_needs_v2___2 Utilities<br>3 subsistence_needs_v2___3 Medicine or any health care (medical, dental, mental health, vision)<br>4 subsistence_needs_v2___4 Phone<br>5 subsistence_needs_v2___5 Clothing<br>6 subsistence_needs_v2___6 Child care<br>7 subsistence_needs_v2___7 Other<br>8 subsistence_needs_v2___8 Does not have any subsistence needs
349	[other_subsistence_v2]<br>Show the field ONLY if: [subsistence_needs_v2(7)] = '1'	If other, please specify	text, Required
350	[maltreatment_treatment_v2]	Section Header: *Maltreatment. (Everyday discrimination scale). In your day to day life, how often have any of the following things happened to you? Please tell me whether you feel that the statement is true almost every day, true at least once a week, true a few times a month, true a few times a year, true less than once a year, or never true for you*<br>You are treated with less courtesy than other people.	radio (Matrix)<br>1 Never<br>2 Less than once a year<br>3 A few times a year<br>4 A few times a month<br>5 At least once a week<br>6 Almost every day<br>7 Don't know or refused

351	[maltreatment_servicev2]	You receive poorer service than other people at restaurants and stores.	radio (Matrix)<br>1 Never<br>2 Less than once a year<br>3 A few times a year<br>4 A few times a month<br>5 At least once a week<br>6 Almost every day<br>7 Don't know or refused
352	[maltreatment_smart_v2]	People act as if they think you aren't smart.	radio (Matrix)<br>1 Never<br>2 Less than once a year<br>3 A few times a year<br>4 A few times a month<br>5 At least once a week<br>6 Almost every day<br>7 Don't know or refused
353	[maltreatment_perception_v2]	People act as if they are afraid of you.	radio (Matrix)<br>1 Never<br>2 Less than once a year<br>3 A few times a year<br>4 A few times a month<br>5 At least once a week<br>6 Almost every day<br>7 Don't know or refused
354	[maltreatment_threat_v2]	You are threatened or harrassed	radio (Matrix)<br>1 Never<br>2 Less than once a year<br>3 A few times a year<br>4 A few times a month<br>5 At least once a week<br>6 Almost every day<br>7 Don't know or refused
355	[covid_housing_v2]	Section Header: *Many things have changed during the COVID-19/coronavirus pandemic. What are some of the changes you have experienced?*<br>I moved from the street to a shelter	radio (Matrix), Required<br>1 yes<br>2 no<br>3 don't know/refused
356	[covid_waitlisthotel_v2]	I am on the waitlist for a hotel/motel	radio (Matrix), Required<br>1 yes<br>2 no<br>3 don't know/refused
357	[covid_increaseinc_v2]	My monthly income went up	radio (Matrix), Required<br>1 yes<br>2 no<br>3 don't know/refused
358	[covid_decreaseinc_v2]	My monthly income went down	radio (Matrix), Required<br>1 yes<br>2 no<br>3 don't know/refused

359	[covid_dx_v2]	I was diagnosed with COVID-19	radio (Matrix), Required<br>1 yes<br>2 no<br>3 don't know/refused
360	[covid_illnessothers_v2]	Someone I know got sick from COVID-19	radio (Matrix), Required<br>1 yes<br>2 no<br>3 don't know/refused
361	[covid_deathothers_v2]	Someone I know died from COVID-19	radio (Matrix), Required<br>1 yes<br>2 no<br>3 don't know/refused
362	[covid_vaccination_v2]	I was vaccinated for COVID-19	radio (Matrix), Required<br>1 yes<br>2 no<br>3 don't know/refused
363	[gender_v2]	Section Header: *Demographics*<br>What terms best express how you describe your gender identity?	radio, Required<br>1 Female<br>2 Male<br>4 Non-Binary<br>3 Transgender<br>5 None of these describe me and I'd like to consider additional options<br>6 Prefer not to answer
364	[gender_cat_v2]<br>Show the field ONLY if: [gender_v2] = '5'	Are any of these a closer description to your gender identity?	radio, Required<br>1 Trans man/Transgender Man/FTM<br>2 Trans woman/Transgender/MTF<br>3 Genderqueer<br>4 Genderfluid<br>5 Gender variant<br>6 Questioning or unsure of your gender identity<br>7 None of these describe me and I want to specify
365	[gender_specificity_v2]<br>Show the field ONLY if: [gender_cat_v2] = '7'	Please specify your gender identification	text, Required
366	[ethnicity_v2]	Ethnicity	radio, Required<br>0 Hispanic or Latino<br>1 NOT Hispanic or Latino<br>2 Unknown / Not Reported
367	[race_v2]	Race	radio, Required<br>0 American Indian/Alaska Native<br>1 Asian<br>2 Native Hawaiian or Other Pacific Islander<br>3 Black or African American<br>4 White<br>5 More Than One Race<br>6 Unknown / Not Reported

368	[education_v2]	Section Header: *Education*<br>What is your highest level of education	radio<br>1 Less than high school<br>2 High school or GED<br>3 Some college<br>4 College or professional training
369	[income_v2]	What is your current MONTHLY household income? Please include salary, SSI, Disability, GA, other public benefits, pensions, interests, etc. for both you and your partner (if applicable	text (number), Required
370	[employment_v2]	Section Header: *Employment Status*<br>Are you currently employed?	yesno, Required<br>1 Yes<br>0 No
371	[recent_lodging_v2]	Section Header: *Housing*<br>Please share where you slept last night?	radio, Required<br>1 In an emergency shelter or safe haven<br>2 In transitional housing<br>3 In an institution (including hospital, jail, prison, juvenile detention facility, long-term care facility, or nursing home)?<br>4 In a place not meant for human habitation (including in a car, unsheltered on the street or under a bridge, etc.)<br>5 In housing you shared with others, but did not own or rent<br>6 In housing that you rented<br>7 In a hotel or motel<br>8 In a short term SRO<br>9 In a shelter in place hotel<br>10 In a permanent supportive housing
372	[lengthofstay_years_v2]	How long have you stayed in the place you stayed at last night (years)?	text (number), Required
373	[lenghtofstay_days_v2]	How long have you stayed in the place you stayed at last night (in days)?	text (number), Required
374	[homeless_continuously_v2]	Have you been continuously homeless in the past 12 months?	radio, Required<br>1 yes<br>2 no<br>3 don't know/refused
375	[episodic_homelessness_v2]	How many episodes of homelessness have you had in the past 3 years?	text (number, Min: 1), Required
376	[rct_participant_survey_followup_complete]	Section Header: *Form Status*<br>Complete?	dropdown<br>0 Incomplete<br>1 Unverified<br>2 Complete
Instrument: **Covid-19 Screening** (covid19_screening)			
377	[study_idcovid]	Section Header: *COVID-19 Screening*<br>Study ID	text, Required
378	[c_screener_descriptive_v2]	In light of the current pandemic, please answer the following questions. If "yes" to any of the following questions, reschedule interview.	descriptive
379	[c_coviddiagnosis_past30_v2]	In the past 30 days, have you or someone you stay with been diagnosed with COVID-19?	yesno, Required<br>1 Yes<br>0 No

380	[c_closecontact_past14_v2]	In the past 14 days, have you had unprotected close contact with someone diagnosed with COVID-19? Close contact is defined as being within 6ft for 15 minutes or longer, and unprotected means without face covering.	yesno, Required<br>1 Yes<br>0 No
381	[c_symptoms_past14_v2]	In the past 14 days, have you had any of these symptoms not explained by a pre-existing condition (e.g., allergies)?	descriptive
382	[c_fever_v2]	Fever, chills, shivering/shakes (temperature > 100�C)	yesno, Required<br>1 Yes<br>0 No
383	[c_sorethroat_v2]	Sore throat	yesno, Required<br>1 Yes<br>0 No
384	[c_runnynose_v2]	Runny or stuffy nose	yesno, Required<br>1 Yes<br>0 No
385	[c_shortnessofbreath_v2]	Difficulty breathing or shortness of breath	yesno, Required<br>1 Yes<br>0 No
386	[c_muscleaches_v2]	Unexplained muscle aches	yesno, Required<br>1 Yes<br>0 No
387	[c_fatigue_v2]	Feeling unusually weak or fatigued	yesno, Required<br>1 Yes<br>0 No
388	[c_lossofsmell_v2]	Loss of sense of taste or smell	yesno, Required<br>1 Yes<br>0 No
389	[c_diarrhea_v2]	Diarrhea	yesno, Required<br>1 Yes<br>0 No
390	[c_eyeredness_v2]	Eye redness with or without discharge ("pink eye")	yesno, Required<br>1 Yes<br>0 No
391	[ci_date_v2]	Section Header: *Basic Information*<br>Date	text (date_mdy), Required
392	[ci_first_name_v2]	First name	text, Required, Identifier
393	[ci_last_name_v2]	Last name	text, Required
394	[ci_site_v2]	Site	text, Required
395	[ci_week_v2]	Week #	text, Required
396	[covid19_screening_complete]	Section Header: *Form Status*<br>Complete?	dropdown<br>0 Incomplete<br>1 Unverified<br>2 Complete
Instrument: **Smoking Abstinence Assessment** (smoking_abstinence_assessment)			
397	[study_idabstinence]	Study ID	text, Required
398	[missed_visit]	Is this a missed visit?	radio<br>1 Not Completed<br>0 Completed<br><br>Custom alignment: RH

399	[ci_date]<br><br>Show the field ONLY if:<br>[missed_visit]='0'	Section Header: *Basic Information*<br><br>Date	text (date_mdy), Required
400	[ci_first_name]<br><br>Show the field ONLY if:<br>[missed_visit]='0'	First name	text, Required, Identifier
401	[ci_last_name]<br><br>Show the field ONLY if:<br>[missed_visit]='0'	Last name	text, Required
402	[ci_site]<br><br>Show the field ONLY if:<br>[missed_visit]='0'	Site	text, Required
403	[ci_week]<br><br>Show the field ONLY if:<br>[missed_visit]='0'	Day	text, Required
404	[co_reading2]<br><br>Show the field ONLY if:<br>[missed_visit]='0'	Carbon Monoxide Reading	text (number), Required
405	[currentcigs_instructions]<br><br>Show the field ONLY if:<br>[missed_visit]='0'	Section Header: *Current use of cigarettes*<br><br>The next set of questions is about your cigarette smoking within the past week.	descriptive
406	[ci_past7]<br><br>Show the field ONLY if:<br>[missed_visit]='0'	Did you smoke any cigarettes in the past 7 days?	yesno<br>1 Yes<br>0 No
407	[ci_past7_cigdays]<br><br>Show the field ONLY if:<br>[ci_past7] = '1' and [missed_visit]='0'	Out of the past 7 days, on how many days did you smoke cigarettes?	text (number, Min: 0)
408	[ci_past7_cigsperday]<br><br>Show the field ONLY if:<br>[ci_past7] = '1' and [missed_visit]='0'	In the past 7 days, on the days that you did smoke, about how many cigarettes did you usually smoke per day?	text (number, Min: 0), Required
409	[ci_past7_quit]<br><br>Show the field ONLY if:<br>[missed_visit]='0'	Within the last week, have you stopped smoking for one day or longer because you were trying to stop smoking?	yesno, Required<br>1 Yes<br>0 No
410	[ci_past7_quithowlong]<br><br>Show the field ONLY if:<br>[ci_past7_quit] = '1' and [missed_visit]='0'	How long were you able to go without smoking during your last quit attempt (number of days) in the past 12 months?	text (number, Min: 1, Max: 365)

411	[ci_past7_products]<br>Show the field ONLY if:<br>[ci_past7_quit] = '1' and [missed_visit]='0'	What products, methods or resources did you use to help you stop smoking within the last week (check all that apply, or "did not try to stop smoking within the last week")?	checkbox<br>1 ci_past7_products___1 Quit "cold turkey"<br>2 ci_past7_products___2 Gradually cut down<br>3 ci_past7_products___3 Smoking cessation class<br>4 ci_past7_products___4 Advice from a healthcare professional (doctor, nurse, psychologist, etc.)<br>5 ci_past7_products___5 Advice from shelter staff<br>6 ci_past7_products___6 Called a telephone hotline<br>7 ci_past7_products___7 Hypnosis<br>8 ci_past7_products___8 Acupuncture<br>9 ci_past7_products___9 Nicotine gum<br>10 ci_past7_products___10 Nicotine patch<br>11 ci_past7_products___11 Nicotine spray<br>12 ci_past7_products___12 Nicotine lozenge<br>13 ci_past7_products___13 Nicotine inhaler<br>14 ci_past7_products___14 Zyban/Wellbutrin for smoking cessation<br>15 ci_past7_products___15 Chantix/Varenicline for smoking cessation<br>16 ci_past7_products___16 E-cigarettes to help cut down or stop using regular cigarettes<br>17 ci_past7_products___17 Smokeless tobacco<br>18 ci_past7_products___18 Other<br>0 ci_past7_products___0 Did not try to stop smoking within the last week
412	[ci_past7_products_other]<br>Show the field ONLY if:<br>[ci_past7_products(18)] = '1' and [missed_visit]='0'	If "other", please explain:	text
413	[ci_support_instructions]<br>Show the field ONLY if:<br>[missed_visit]='0'	Section Header: *Smoking cessation support*<br>The next set of questions is about any smoking cessation support or guidance you may have received in the last week.	descriptive
414	[ci_encounters_any]<br>Show the field ONLY if:<br>[missed_visit]='0'	Since the last visit, did you have any encounters with your PCP or other clinic staff about smoking?	yesno, Required<br>1 Yes<br>0 No
415	[ci_encounters_howmany]<br>Show the field ONLY if:<br>[ci_encounters_any] = '1' and [missed_visit]='0'	How many of these encounters did you have since the last visit?	text
416	[ci_medications_received]<br>Show the field ONLY if:<br>[missed_visit]='0'	Since the last visit, did you receive smoking cessation medications from your Primary Care Provider?	yesno, Required<br>1 Yes<br>0 No
417	[ci_medications_use]<br>Show the field ONLY if:<br>[missed_visit]='0'	Since the last visit, did you use any smoking cessation medications provided to you by your Primary Care Provider?	yesno, Required<br>1 Yes<br>0 No

418	[ci_medications_whichones]<br><br>Show the field ONLY if:<br>[ci_medications_use] = '1' and [missed_visit]='0'	Within the past week, what type of smoking cessation medication did you use? Check all that apply.	checkbox, Required<br>1 ci_medications_whichones___1 Patch<br>2 ci_medications_whichones___2 Gum<br>3 ci_medications_whichones___3 Lozenge<br>4 ci_medications_whichones___4 Chantix<br>5 ci_medications_whichones___5 Bupropion<br>6 ci_medications_whichones___6 Other
419	[ci_medications_other]<br><br>Show the field ONLY if:<br>[ci_medications_whichones(6)] = '1' and [missed_visit]='0'	If "other", please explain	text
420	[ci_medications_howmany]<br><br>Show the field ONLY if:<br>[ci_medications_use] = '1' and [missed_visit]='0'	Out of the past 7 days, on how many days did you use these medications?	text
421	[ci_medications_whynot]<br><br>Show the field ONLY if:<br>[ci_medications_use] = '0'	If you did not use the medications every day, why not? Check all that apply.	checkbox, Required<br>1 ci_medications_whynot___1 Bad taste<br>2 ci_medications_whynot___2 Side effects<br>3 ci_medications_whynot___3 Cost<br>4 ci_medications_whynot___4 Didn't fill prescription<br>5 ci_medications_whynot___5 Medications were stolen or lost<br>6 ci_medications_whynot___6 Encountered insurance problems<br>7 ci_medications_whynot___7 Didn't feel like they were working<br>8 ci_medications_whynot___8 Don't believe in NRT<br>9 ci_medications_whynot___9 Used medications every day<br>0 ci_medications_whynot___0 Other
422	[ci_medications_whynotother]<br><br>Show the field ONLY if:<br>[ci_medications_whynot(0)] = '1' and [missed_visit]='0'	If "other", please explain	text
423	[abstinence]<br><br>Show the field ONLY if:<br>[missed_visit]='0'	Did the participant demonstrate smoking abstinence at this visit?	yesno<br>1 Yes<br>0 No
424	[smoking_abstinence_assessment_complete]	Section Header: *Form Status*<br>Complete?	dropdown<br>0 Incomplete<br>1 Unverified<br>2 Complete
Instrument: **Payment** (payment)			
425	[study_idpayment]	Study ID	text, Required
426	[payment_header]	Group: [initial_data_arm_1][random_grp] Visit Abstinence Streak Payment Visit Earned Week 1 Day 1 [week_1_day_1_arm_1][abstinence] [week_1_day_1_arm_1][streak] [week_1_day_1_arm_1][payment] [week_1_day_1_arm_1][missed_visit] [week_1_day_1_arm_1][earned] Week 1 Day 2 [week_1_day_2_arm_1][abstinence] [week_1_day_2_arm_1][streak] [week_1_day_2_arm_1][payment] [week_1_day_2_arm_1][missed_visit]	descriptive

		[week_1_day_2_arm_1][earned] Week 1 Day 3 [week_1_day_3_arm_1][abstinence] [week_1_day_3_arm_1][streak] [week_1_day_3_arm_1][payment] [week_1_day_3_arm_1][missed_visit] [week_1_day_3_arm_1][earned] Week 1 Day 4 [week_1_day_4_arm_1][abstinence] [week_1_day_4_arm_1][streak] [week_1_day_4_arm_1][payment] [week_1_day_4_arm_1][missed_visit] [week_1_day_4_arm_1][earned] Week 1 Day 5 [week_1_day_5_arm_1][abstinence] [week_1_day_5_arm_1][streak] [week_1_day_5_arm_1][payment] [week_1_day_5_arm_1][missed_visit] [week_1_day_5_arm_1][earned] Week 1 Day 6 [week_1_day_6_arm_1][abstinence] [week_1_day_6_arm_1][streak] [week_1_day_6_arm_1][payment] [week_1_day_6_arm_1][missed_visit] [week_1_day_6_arm_1][earned] Week 1 Day 7 [week_1_day_7_arm_1][abstinence] [week_1_day_7_arm_1][streak] [week_1_day_7_arm_1][payment] [week_1_day_7_arm_1][missed_visit] [week_1_day_7_arm_1][earned] Week 2 Visit 1 [week_2_followup_vi_arm_1][abstinence] [week_2_followup_vi_arm_1][streak] [week_2_followup_vi_arm_1][payment] [week_2_followup_vi_arm_1][missed_visit] [week_2_followup_vi_arm_1][earned] Week 2 Visit 2 [week_2_visit_2_arm_1][abstinence] [week_2_visit_2_arm_1][streak] [week_2_visit_2_arm_1][payment] [week_2_visit_2_arm_1][missed_visit] [week_2_visit_2_arm_1][earned] Week 3 Visit 1 [week_3_visit_1_arm_1][abstinence] [week_3_visit_1_arm_1][streak] [week_3_visit_1_arm_1][payment] [week_3_visit_1_arm_1][missed_visit] [week_3_visit_1_arm_1][earned] Week 3 Visit 2 [week_3_visit_2_arm_1][abstinence] [week_3_visit_2_arm_1][streak] [week_3_visit_2_arm_1][payment] [week_3_visit_2_arm_1][missed_visit] [week_3_visit_2_arm_1][earned] Week 4 Visit 1 [week_4_followup_vi_arm_1][abstinence] [week_4_followup_vi_arm_1][streak] [week_4_followup_vi_arm_1][payment] [week_4_followup_vi_arm_1][missed_visit] [week_4_followup_vi_arm_1][earned] Week 4 Visit 2 [week_4_visit_2_arm_1][abstinence] [week_4_visit_2_arm_1][streak] [week_4_visit_2_arm_1][payment] [week_4_visit_2_arm_1][missed_visit] [week_4_visit_2_arm_1][earned] Week 5 [week_5_visit_1_arm_1][abstinence] [week_5_visit_1_arm_1][streak] [week_5_visit_1_arm_1][payment] [week_5_visit_1_arm_1][missed_visit] [week_5_visit_1_arm_1][earned] Week 6 [week_6_visit_1_arm_1][abstinence] [week_6_visit_1_arm_1][streak] [week_6_visit_1_arm_1][payment] [week_6_visit_1_arm_1][missed_visit] [week_6_visit_1_arm_1][earned] Week 7 [week_7_visit_1_arm_1][abstinence] [week_7_visit_1_arm_1][streak] [week_7_visit_1_arm_1][payment] [week_7_visit_1_arm_1][missed_visit] [week_7_visit_1_arm_1][earned] Week 8 [week_8_visit_1_arm_1][abstinence] [week_8_visit_1_arm_1][streak] [week_8_visit_1_arm_1][payment] [week_8_visit_1_arm_1][missed_visit] [week_8_visit_1_arm_1][earned] Week 9 [week_9_visit_1_arm_1][abstinence] [week_9_visit_1_arm_1][streak] [week_9_visit_1_arm_1][payment] [week_9_visit_1_arm_1][missed_visit] [week_9_visit_1_arm_1][earned] Week 10 [week_10_visit_1_arm_1][abstinence] [week_10_visit_1_arm_1][streak] [week_10_visit_1_arm_1][payment] [week_10_visit_1_arm_1][missed_visit] [week_10_visit_1_arm_1][earned] Week 11 [week_11_visit_1_arm_1][abstinence] [week_11_visit_1_arm_1][streak] [week_11_visit_1_arm_1][payment] [week_11_visit_1_arm_1][missed_visit] [week_11_visit_1_arm_1][earned] Week 12 [week_12_followup_v_arm_1][abstinence] [week_12_followup_v_arm_1][streak] [week_12_followup_v_arm_1][payment] [week_12_followup_v_arm_1][missed_visit] [week_12_followup_v_arm_1][earned] Week 13 [week_13_visit_1_arm_1][abstinence] [week_13_visit_1_arm_1][streak] [week_13_visit_1_arm_1][payment]	

		[week_13_visit_1_arm_1][missed_visit] [week_13_visit_1_arm_1][earned] Week 16 [week_16_visit_1_arm_1][abstinence] [week_16_visit_1_arm_1][streak] [week_16_visit_1_arm_1][payment] [week_16_visit_1_arm_1][missed_visit] [week_16_visit_1_arm_1][earned] Week 20 [week_20_visit_1_arm_1][abstinence] [week_20_visit_1_arm_1][streak] [week_20_visit_1_arm_1][payment] [week_20_visit_1_arm_1][missed_visit] [week_20_visit_1_arm_1][earned] Week 24 [week_24_followup_v_arm_1][abstinence] [week_24_followup_v_arm_1][streak] [week_24_followup_v_arm_1][payment] [week_24_followup_v_arm_1][missed_visit] [week_24_followup_v_arm_1][earned]	
427	[payday]	Date	text (date_mdy)
428	[visits_completed]	Visits Completed	calc<br>Calculation: if([missed_visit]='0',1,0)+if([previous-event-name][visits_completed]<>'',[previous-event-name][visits_completed],0)
429	[previous_streak]	Previous Streak	calc<br>Calculation: if([previous-event-name][streak]<>'', [previous-event-name][streak],0)
430	[streak]	Streak:	calc<br>Calculation: if([missed_visit]='0',[abstinence]+[abstinence]*[previous-event-name][streak],if([missed_visit]='1',0,''))
431	[adj_ex]	Payment Adjustment Example: Abstinent and attended streak >2 after having missed a visit, add an increase in payment to equal the previous maximum payment. �Visit 1, attended and abstinent: $13 �Visit 2, attended and abstinent: $13.50, total $26.50 �Visit 3, attended and abstinent: $14, total $40.40 �Visit 4, attended and abstinent: $14.50, total $54.9 �Visit 5, attended and abstinent: $15, total $69.9 Visit 6, attended and abstinent: $15.50 , total $85.4 Visit 7, missed Visit 8, attended and abstinent: $13, total $98.4 Visit 9, attended and abstinent: $13.50 , total $111.9 Visit 10, attended and abstinent: reset to $15.50 , total $127.4 - there is a $2 dollar increase here in the amount as an added bonus to attending visits and stopping smoking.	descriptive
432	[pay_adjust]	Payment Adjustment:<br>If a participant misses a visit, the Carried-over Adjustment needs to be recalibrated.	text<br>Field Annotation: @DEFAULT='0'
433	[previous_tot_adjust]	Previous Total Adjustment	calc<br>Calculation: if([previous-event-name][tot_adjust]<>'', [previous-event-name][tot_adjust],0)
434	[tot_adjust]	Carried-over adjustment:	calc<br>Calculation: if([previous-event-name][tot_adjust]<>'', [previous-event-name][tot_adjust]+[pay_adjust], [pay_adjust])
435	[payment]	Payment Due:<br>missed visit: [missed_visit]<br>random grp: [initial_data_arm_1][random_grp]<br>abstinence: [abstinence]<br>previous streak: [previous_streak]<br>Total Adjustment: [tot_adjust]	calc<br>Calculation: if([missed_visit]='0' and [initial_data_arm_1][random_grp]='1' ,sum([abstinence]*13, 0.50*[abstinence]*[previous_streak],[tot_adjust]) ,if([missed_visit]='0' and [initial_data_arm_1][random_grp]='2',5 ,if([missed_visit]='1',0,'')))
436	[test]	TEST	calc<br>Calculation: if([missed_visit]='0' and [initial_data_arm_1][random_grp]='1', 1,0)
437	[earned]	Amount Earned including current visit:	calc<br>Calculation: if([missed_visit]='0',[payment]+[previous-event-name][earned],if([missed_visit]='1',[previous-event-name][earned],''))

438	[paid]	Completed this visit	radio<br>1 Completed<br>0 Not Completed<br><br>Custom alignment: RH<br>Field Annotation: @HIDDEN-FORM
439	[payment_complete]	Section Header: *Form Status*<br>Complete?	dropdown<br>0 Incomplete<br>1 Unverified<br>2 Complete
