# Supplementary material for: Contingency management to promote smoking cessation in people experiencing homelessness: Leveraging the electronic health record in a pilot, pragmatic randomized controlled trial
Source: PLoS One. 2022 Dec 16;17(12):e0278870. doi: 10.1371/journal.pone.0278870 (PMC9757562; doi:10.1371/journal.pone.0278870)
Supplement: S3 File — (PDF) [file pone.0278870.s004.pdf]

Data Dictionary Codebook

09/06/2022 8:56pm

	#	Variable / Field Name	Field Label<br>*Field Note*	Field Attributes (Field Type, Validation, Choices, Calculations, etc.)
Instrument: **Followup Questionnaire** (followup_questionnaire)				
	1	[record_id]	Study ID	text
	2	[participant_id]	Participant ID	text, Required, Identifier
	3	[today_date]	Today's Date	text (date_mdy, Min: 2022-03-01), Required
	4	[first_name_9f15d2]	First Name	text, Required, Identifier
	5	[last_name_b9f995]	Last Name	text, Required, Identifier
	6	[clinic_name]	Clinic Name	dropdown, Required<br>1 Tom Waddell Urban Health Clinic<br>2 Richard H. Fine People's Clinic<br>3 Positive Health Program Clinic
	7	[co_reading]	CO Reading	text (number, Min: 000, Max: 100), Required
	8	[checkin_month]	Section Header: *Follow-up Questions*<br>Check-in Month	dropdown, Required<br>1 7-Month Check-in<br>2 8-Month Check-in<br>3 9-Month Check-in<br>4 10-Month Check-in<br>5 11-Month Check-in
	9	[checkin_answer]	Check-in Completed?	yesno, Required<br>1 Yes<br>0 No
	10	[incentive_given]<br>Show the field ONLY if:<br>[checkin_answer] = '1'	Incentive given?	yesno, Required<br>1 Yes<br>0 No
	11	[followup_questionnaire_complete]	Section Header: *Form Status*<br>Complete?	dropdown<br>0 Incomplete<br>1 Unverified<br>2 Complete
