# Supplementary material for: Contingency management to promote smoking cessation in people experiencing homelessness: Leveraging the electronic health record in a pilot, pragmatic randomized controlled trial
Source: PLoS One. 2022 Dec 16;17(12):e0278870. doi: 10.1371/journal.pone.0278870 (PMC9757562; doi:10.1371/journal.pone.0278870)
Supplement: S4 File — (PDF) [file pone.0278870.s005.pdf]

Smoking Abstinence Assessment

Participant ID ______________________

Study ID ______________________

Is this a missed visit? ◯ Not Completed ◯ Completed

Basic Information

Date ______________________

First name ______________________

Last name ______________________

Site ______________________

Day ______________________

Carbon Monoxide Reading ______________________

Current use of cigarettes

The next set of questions is about your cigarette smoking within the past week.

Did you smoke any cigarettes in the past 7 days?
◯ Yes
◯ No

Out of the past 7 days, on how many days did you smoke cigarettes? ______________________

In the past 7 days, on the days that you did smoke, about how many cigarettes did you usually smoke per day? ______________________

Within the last week, have you stopped smoking for one day or longer because you were trying to stop smoking?
◯ Yes
◯ No

How long were you able to go without smoking during your last quit attempt (number of days) in the past 12 months? ______________________

What products, methods or resources did you use to help you stop smoking within the last week (check all that apply, or "did not try to stop smoking within the last week")?

- ☐ Quit "cold turkey"
- ☐ Gradually cut down
- ☐ Smoking cessation class
- ☐ Advice from a healthcare professional (doctor, nurse, psychologist, etc.)
- ☐ Advice from shelter staff
- ☐ Called a telephone hotline
- ☐ Hypnosis
- ☐ Acupuncture
- ☐ Nicotine gum
- ☐ Nicotine patch
- ☐ Nicotine spray
- ☐ Nicotine lozenge
- ☐ Nicotine inhaler
- ☐ Zyban/Wellbutrin for smoking cessation
- ☐ Chantix/Varenicline for smoking cessation
- ☐ E-cigarettes to help cut down or stop using regular cigarettes
- ☐ Smokeless tobacco
- ☐ Other
- ☐ Did not try to stop smoking within the last week

If "other", please explain: ______________________________

Smoking cessation support

The next set of questions is about any smoking cessation support or guidance you may have received in the last week.

Since the last visit, did you have any encounters with your PCP or other clinic staff about smoking?

- ◯ Yes
- ◯ No

How many of these encounters did you have since the last visit? ______________________________

Since the last visit, did you receive smoking cessation medications from your Primary Care Provider?

- ◯ Yes
- ◯ No

Since the last visit, did you use any smoking cessation medications provided to you by your Primary Care Provider?

- ◯ Yes
- ◯ No

Within the past week, what type of smoking cessation medication did you use? Check all that apply.

- ☐ Patch
- ☐ Gum
- ☐ Lozenge
- ☐ Chantix
- ☐ Bupropion
- ☐ Other

If "other", please explain ______________________________

Out of the past 7 days, on how many days did you use these medications? ______________________________

If you did not use the medications every day, why not? Check all that apply.	☐ Bad taste<br>☐ Side effects<br>☐ Cost<br>☐ Didn't fill prescription<br>☐ Medications were stolen or lost<br>☐ Encountered insurance problems<br>☐ Didn't feel like they were working<br>☐ Don't believe in NRT<br>☐ Used medications every day<br>☐ Other
If "other", please explain	__________________________________
Did the participant demonstrate smoking abstinence at this visit?	◯ Yes<br>◯ No
